# Biophysical and functional characterization of the *Escherichia coli* Dr fimbrial envelope reveals its importance for pathogenesis and lithogenesis

**DOI:** 10.1371/journal.ppat.1014588

**Published:** 2026-09-16

**Authors:** Michalina Nagórka, Beata Zalewska-Piątek, Adam Iwanicki, Paweł Wityk, Slawomir Jakiela, Weronika Switlik, Piotr Bruździak, Miłosz Wieczór, Vanessa Liévin-Le Moal, Joanna Raczak-Gutknecht, Przemysław Gnatowski, Mateusz Ficek, Angelika Łepek, Magdalena Narajczyk, Rafał Piątek

**Affiliations:** 1 Department of Biotechnology and Microbiology, Faculty of Chemistry, Gdańsk University of Technology, Gdańsk, Poland; 2 Division of Molecular Bacteriology, Intercollegiate Faculty of Biotechnology UG&MUG, Medical University of Gdańsk, Gdańsk, Poland; 3 Division of Biopharmacy and Pharmacokinetics, Faculty of Pharmacy, Medical University of Gdańsk, Gdańsk, Poland; 4 Department of Physics and Biophysics, Institute of Biology, Warsaw University of Life Sciences, Warsaw, Poland; 5 Centre for Advanced Materials and Technologies, Warsaw University of Technology, Warsaw, Poland; 6 Department of Physical Chemistry, Faculty of Chemistry, Gdańsk University of Technology, Gdańsk, Poland; 7 Molecular Modeling and Bioinformatics, IRB Barcelona, Barcelona, Spain; 8 University Paris-Saclay, INSERM, UMR-S 996 Inflammation, Microbiome and Immunosurveillance, Orsay, France; 9 Paris Center for Microbiome Medicine (PaCeMM) FHU, Paris, France; 10 Department of Polymer Technology, Faculty of Chemistry, Gdańsk University of Technology, Gdańsk, Poland; 11 Department of Electronics, Telecommunications and Informatics, Gdańsk University of Technology, Gdańsk, Poland; 12 Faculty of Applied Physics and Mathematics, Institute of Nanotechnology and Materials Engineering, Gdańsk University of Technology, Gdańsk, Poland; 13 Bioimaging Laboratory, Faculty of Biology, University of Gdańsk, Gdańsk, Poland; INSERM U1220, FRANCE

## Abstract

Uropathogenic *Escherichia coli* strains rely on specialized surface appendages to colonize the urinary tract, including Dr fimbriae, which are strongly associated with recurrent infection and long-term persistence. Although traditionally described as receptor-binding fibers, their broader contribution to bacterial surface architecture and physicochemical behavior has remained unclear. Here, combining quantitative proteomics, computational modeling, rheology, surface physicochemistry, and flow-based adhesion assays, we show that Dr fimbriae collectively form a dense, highly hydrated amphipathic envelope surrounding the bacterial cell. Rather than functioning as isolated filaments, they generate a dynamic supramolecular layer approximately 200–300 nm thick that markedly alters whole-cell properties. Mechanistic analyses performed in a simplified laboratory model were validated in the clinical UPEC strain IH11128, demonstrating that the principal physicochemical and adhesive properties of the Dr envelope are preserved despite phase-variable fimbrial expression. The Dr envelope strongly enhanced adhesion to abiotic surfaces, particularly hydrophilic glass, where Dr-positive bacteria resisted nanonewton-range capillary detachment forces, indicating stable multipoint anchoring. The envelope also promoted selective binding to calcium oxalate (CaOx), the principal mineral component of kidney stones, under urinary conditions. In the clinical isolate, CaOx binding occurred almost exclusively within the Dr-positive subpopulation and was largely abolished by anti-Dr antibodies, demonstrating that mineral recognition depends specifically on Dr fimbriae. Beyond adhesion, the fimbrial mesh functioned as a size-selective molecular filter. Dr-positive bacteria showed markedly reduced susceptibility to large bacteriophages, whereas infection by smaller phages and susceptibility to low-molecular-weight antibiotics remained unchanged, indicating steric exclusion of large biological particles without restricting diffusion of small solutes. Together, these findings establish the Dr envelope as a multifunctional supramolecular surface architecture that governs bacterial surface physicochemistry, promotes stable urinary tract colonization, mediates CaOx recognition, and expands the adaptive capabilities of UPEC within the urinary tract.

## Author summary

Urinary tract infections, primarily caused by *Escherichia coli*, are among the most common bacterial infections, and recurrent disease is frequently associated with kidney stone formation. Some uropathogenic strains produce Dr fimbriae, surface fibers previously viewed mainly as specialized structures that help bacteria attach to host cells. Here we show that their role is much broader. We found that Dr fimbriae collectively assemble into a dense, hydrated envelope surrounding the bacterial cell, fundamentally changing the physical and chemical properties of its surface. Using a simplified laboratory model and validating the key findings in a clinical UPEC strain, we demonstrate that this envelope strengthens bacterial attachment to surfaces exposed to mechanical stress, promotes selective binding to CaOx crystals, the principal component of most kidney stones, and acts as a molecular filter that limits access of large bacteriophages while remaining permeable to small molecules. These findings identify the Dr envelope as a previously unrecognized surface architecture that may enhance bacterial persistence in the urinary tract and contribute to kidney stone-associated infections.

## Introduction

Urinary tract infections (UTIs) are among the most common bacterial infections worldwide, with approximately 400 million cases reported each year [1]. Uropathogenic *Escherichia coli* (UPEC) strains are responsible for 80–90% of community-acquired and recurrent UTIs [2]. Most UTIs represent autoinfections caused by UPEC strains originating from the intestinal microbiota, where these bacteria typically do not induce disease symptoms [3]. For UPEC, passage from the gut to the bladder represents a major environmental transition defined not only by the physicochemical properties of urine, including pH, osmolarity, concentrations of urea, ammonium, and acidic mucopolysaccharides, but also by nutrient-limited conditions with different carbon sources and few competing microorganisms. In addition, bacteria colonizing the urinary tract must cope with its distinct physiological characteristics, including continuous urine flow and periodic bladder emptying. UPEC strains are able to grow efficiently in the urinary tract environment during active human urinary tract infection [4,5]. Importantly, UPEC strains also display enhanced growth in human urine (HU) *in vitro*, achieving an approximately threefold shorter generation time and fourfold higher final optical density compared with reference non-uropathogenic *E. coli* strains [6]. These observations suggest that UPEC strains harbor specific adaptations that enable survival, growth, and persistence in an environment shaped by the chemical composition and hydrodynamics of urine in the bladder and kidneys.

Among UPEC isolates, the most prevalent pandemic multilocus sequence type (ST) is ST131, which is strongly associated with resistance to multiple classes of antibiotics, including fluoroquinolones, third-generation cephalosporins, polymyxins, and carbapenems [7–10]. Functional genomic and metabolomic analyses of the UPEC ST131 strain EC958 identified 24 genes required for efficient growth in urine, including genes involved in the metabolism of small peptides, amino acids and nucleotides, as well as pathways associated with the stringent response, lipopolysaccharide biosynthesis, fluoride resistance, and the utilization of L-lactate as a major carbon source in HU [6]. Interestingly, approximately 35% of ST131 strains encode adhesins belonging to the Afa/Dr family characteristic of UPEC strains causing recurrent cystitis and pyelonephritis, particularly in pregnant women [11–14]. Dr fimbriae, encoded by the *dra* operon, were among the first adhesins of the Afa/Dr family to be identified [15]. Fimbriae (also called pili) structures are assembled via the chaperone–usher pathway conserved in Gram-negative bacteria [16,17]. Dr fimbriae are linear homopolymers composed of the major structural subunit DraE, with the minor adhesin DraD located at the distal end of the fiber [18–20]. The DraB chaperone catalyzes the folding of DraE in the periplasm, whereas the outer membrane usher DraC mediates the assembly of the mature fimbrial structure [17]. Dr fimbriae function as polyadhesins because each DraE subunit is capable of recognizing the Dr blood group antigen of human decay-accelerating factor (DAF), type IV collagen, and members of the carcinoembryonic antigen (CEA) family [21–24]. Unlike heteropolymeric fimbriae, Dr fimbriae do not form a quaternary helical structure, and their diameter of approximately 2 nm corresponds to that of a single DraE subunit [16]. On the bacterial surface they form a loose network of curled fibrils, giving the appearance of an amorphous capsule in transmission electron microscopy (TEM) [15]. Dr-expressing bacteria bind to host cells according to a slip-bond mechanism, in which the number of attached bacteria decreases with increasing shear stress. Nevertheless, Dr-fimbriated bacteria remain capable of effective adhesion across the range of shear forces generated by urine flow in the urinary tract [25].

Dr fimbriae belong to the FGL subgroup of chaperone–usher (CU) adhesins, which are characterized by the presence of an extended F1–G1 loop in the periplasmic chaperone and typically assemble into thin, flexible fibers [26]. In electron microscopy, they appear as poorly resolved fibrils or capsule-like surface layers surrounding the bacterial cell. In *Yersinia* *pestis*, the F1 “capsular antigen”, a major virulence factor, is composed of polymerized Caf1A subunits [27], while the pH6 antigen, “thin fibrillar structures” composed of PsaA, contributes to adhesion during the early stages of infection [28,29]. *Yersinia enterocolitica* uses “flexible fibrillae” composed of MyfA subunits to attach to epithelial intestinal cells during yersiniosis [29,30]. Similarly, enterotoxigenic *E. coli* (ETEC) adhere to intestinal epithelial cells via “very thin fibrillar structures” forming the colonization factor CS6 composed of CssA and CssB subunits [31]. Aggregative adherence fimbriae (AAF/I–III) of enteroaggregative *E. coli* (EAEC), comprising AggA, AafA and Agg3A subunits, respectively, form diffuse “flexible fibrillar fimbriae” responsible for the characteristic adherence phenotype in persistent diarrheal disease [32,33]. Finally, beads-on-a-string “thin flexible fibers” consisting mainly of SafA/SafD contribute to intestinal colonization by *Salmonella enterica* during salmonellosis [34].

In contrast, UPEC-encoded type 1 and P fimbriae assembled via the FGS (F1–G1 short) chaperone–usher pathway exhibit a markedly different morphology. In TEM images, type 1 and P fimbriae appear as distinct adhesive organelles measuring 0.2–2.0 µm in length and 5–7 nm in diameter [35]. They are highly structured heteropolymeric organelles consisting of a long helical rod formed by the FimA or PapA proteins, respectively, which is terminated by a short flexible fibrillum carrying a single adhesive subunit, FimH or PapG. Up to 500 type 1 fimbriae, each composed of 500–3000 FimA subunits, may be present on the bacterial surface [36]. Type 1- or P-fimbriated bacteria adhere to host cells via a catch-bond mechanism, in which maximal attachment occurs at a specific non-zero level of shear stress [37]. Below this value the adhesive subunit undergoes a conformational rearrangement that results in dissociation from the receptor molecule [38]. This mechanism protects bacteria from being removed from the bladder together with exfoliated epithelial cells during urination [39–41].

The function of bacterial adhesive organelles is not limited to specific interactions with host receptors. As the outermost structures of the bacterial cell envelope, they contribute to determining its physicochemical properties critical for successful colonization of a given niche. Extensive surface appendages such as Dr fimbriae or type 1 fimbriae can also influence such activities as motility, autoaggregation, and biofilm formation. However, only a limited number of studies have addressed the influence of type 1 and P fimbriae on cellular properties beyond receptor binding. For example, the presence of type 1 fimbriae reduces the negative zeta potential of the bacterial surface, potentially facilitating adhesion to host cells and abiotic surfaces that also typically carry a negative charge [42,43]. Type 1-fimbriated bacteria also exhibit increased surface hydrophobicity as measured by microbial adhesion to hydrocarbons (MATH) and microsphere adhesion to cells (MAC) assays [42–44]. Type 1 fimbriae influence colony morphology of *E. coli* K-12 strains, producing small, compact, convex colonies [45], and abolish bacterial aggregation mediated by surface proteins such as Ag43, AIDA, TibA and EhaA, which require close cell-to-cell contact to form stable intercellular junctions; protruding fimbrial fibers mechanically interfere with this process [46–49]. Finally, type 1 fimbriae promote efficient biofilm formation in *E. coli* and *Klebsiella pneumoniae* [50,51]. Interestingly, both receptor binding and biofilm formation mediated by type 1 fimbriae can be impaired by the presence of large polysaccharide capsules comparable in size to the length of the fimbriae [51].

In this work, we investigate how the Dr fimbrial envelope influences the physicochemical and biological properties of uropathogenic *E. coli*. To define the intrinsic properties of this surface architecture, we first employed a simplified experimental model in which a plasmid encoding Dr fimbriae was introduced into a laboratory *E. coli* strain lacking type 1 fimbriae, enabling direct comparison of otherwise isogenic Dr^+^ and Dr^−^ variants. This genetically defined system allowed comprehensive structural, physicochemical, and functional characterization of the Dr envelope and the development of a quantitative mechanistic model describing its organization and its consequences for bacterial surface behavior over the physiologically relevant urinary pH range. Using this model, we show that the Dr envelope profoundly alters bacterial surface properties, promotes stable adhesion to abiotic surfaces, mediates selective interactions with CaOx, and functions as a size-selective barrier limiting bacteriophage access to the bacterial surface. We then examined whether these principal properties are preserved in the clinical UPEC strain IH11128 by comparison with its isogenic Dr-deficient mutant DR14. Together, these complementary approaches establish the Dr fimbrial envelope as a multifunctional surface architecture whose biological significance extends well beyond receptor-specific adhesion.

## Results

### Relative abundance of Dr fimbriae in the model strain AAEC191A Dr^+^ and the clinical strain IH11128

To validate AAEC191A Dr^+^ as a model for investigating the physicochemical properties of the Dr envelope, the relative abundance of Dr fimbriae was first compared with that of the clinical strain IH11128. Because plasmid pCC90, responsible for Dr fimbriae production in the model strain, carries the *dra* operon without the regulatory region responsible for phase variation [52], this comparison was necessary to assess the physiological relevance of the model.

Overnight cultures of AAEC191A Dr^+^ and IH11128 grown in liquid M63 medium supplemented with 1% casamino acids and 0.2% glycerol were analyzed after normalization to identical optical density (OD_600_). Western blot analysis of isolated fimbrial fractions showed that IH11128 produced DraE, corresponding to the Dr fimbrial monomer, at 20  ± 5% of the level observed in AAEC191A Dr^+^ (S1A Fig). Immunofluorescence analysis of the same cultures showed that all AAEC191A Dr^+^ cells displayed Dr fimbriae-associated fluorescence, whereas only 16 ± 4% of IH11128 cells were fluorescent (S1B Fig). After correction for the fraction of fimbriae-producing cells, Dr fimbriae production in AAEC191A Dr^+^ was found to be comparable to that estimated for the fimbriae-positive subpopulation of IH11128.

### Production of Dr envelope alters the macroscopic properties of bacterial cultures

**Fig 1:**
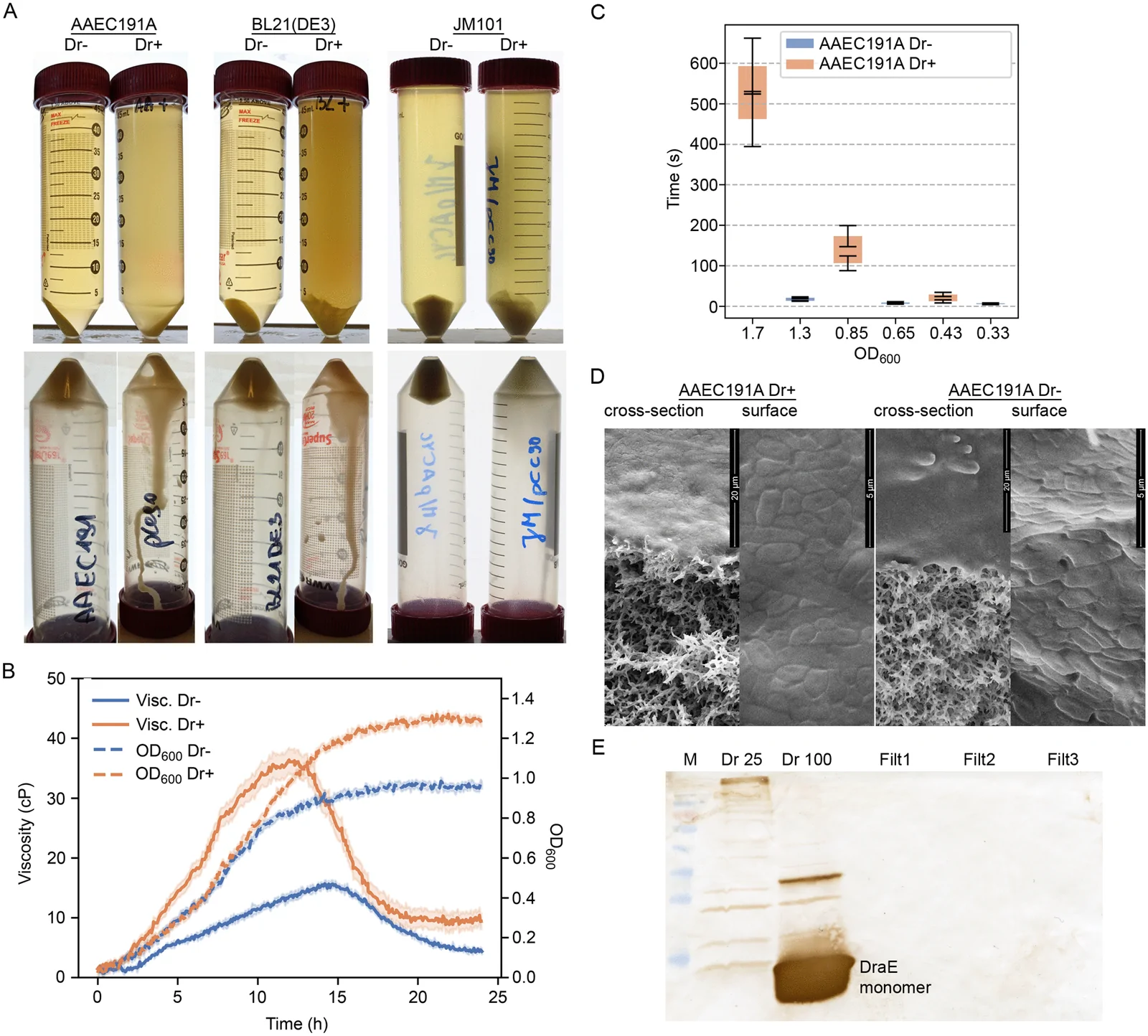
Macroscopic manifestations of Dr fimbrial envelope formation in *Escherichia coli.* (A) Representative macroscopic appearance of overnight cultures of *E. coli* AAEC191A, BL21(DE3) and JM101 carrying either the control plasmid pACYC177 (Dr^−^) or the Dr fimbriae expression plasmid pCC90 (Dr^+^) after centrifugation for 15 min at 1240 × g (upper panel), followed by decanting of the supernatant and 5 min incubation in an inverted position (lower panel), demonstrating decreased cohesion of Dr^+^ bacterial pellets. (B) Apparent viscosity changes monitored as the relationship between optical density (OD_600_) and time for AAEC191A Dr^+^ and Dr^−^ cultures measured in a microfluidic system using 2.16 µl droplets flowing through channels with a cross-sectional area of 600 × 600 µm. The experiment was repeated five times; averaged values are shown, and twice the mean standard deviation is represented by the width of each curve. (C) Filtration time of AAEC191A Dr^+^ and Dr^−^ suspensions through a 0.45 µm cellulose filter in 100 mM potassium nitrate as a function of OD_600_. Dr^+^ suspensions with 1.3-fold higher OD_600_ were used to obtain comparable cell numbers to Dr^−^ suspensions. The lower point indicates the appearance of the first filter area free of suspension, whereas the upper point indicates complete disappearance of liquid from the filter surface. Data represent mean ± SD (n = 3), statistical significance was evaluated using non-parametric Kruskal-Wallis test, showing a significant effect for Dr^+^ filtration (p < 0.001), whereas no significant differences were observed for Dr^−^ filtration (p > 0.05). (D) Representative scanning electron microscopy images of bacterial layers formed after filtration of Dr^+^ and Dr^−^ suspensions with OD_600_ values of 1.7 and 1.3, respectively. (E) Western blot analysis of fimbrial fractions isolated from Dr^+^ cells used in the filtration assay, with detection of DraE protein. Lane Dr25: fimbrial fraction incubated in Laemmli buffer at 25 °C before electrophoresis; lane Dr100: the same fraction incubated at 100 °C; lanes Filt1, Filt2, and Filt3: 100-fold concentrated filtrates obtained after filtration of AAEC191A Dr^+^ suspensions with OD_600_ values of 1.7, 0.85, and 0.43, respectively.

The presence of Dr fimbriae was readily visible in bacterial cultures, as reflected by the clear macroscopic differences between Dr^+^ and Dr^−^ strains. The influence of Dr fimbriae on bacterial surface properties was evident during centrifugation of overnight cultures. Control Dr^−^ strains (AAEC191A, BL21(DE3), and JM101) grown to OD_600_ ≈ 5 produced a clear supernatant after centrifugation, whereas the corresponding Dr^+^ cultures remained visibly turbid (OD_600_ ≈ 0.5). In addition, pellets formed by Dr^−^ strains were compact and stable, whereas pellets obtained from Dr^+^ strains were loose and rapidly dispersed after supernatant removal (Fig 1A).

To quantify this effect, culture viscosity was measured using a non-invasive microfluidic method allowing simultaneous determination of viscosity and optical density in a moving microdroplet. Continuous measurements performed for AAEC191A Dr^−^ and AAEC191A Dr^+^ cultures grown in Luria-Bertani (LB) broth for 24 h showed similar viscosities during early growth (1–2 cP), followed by a marked increase in the Dr^+^ culture after ~3 h (Fig 1B). Viscosity reached ~22 cP at 8 h and ~35–38 cP at 12 h in the Dr^+^ culture, compared with ~8 cP and ~12–15 cP, respectively, in the Dr^−^ control. From ~6 h onward, AAEC191A Dr^+^ cultures also showed consistently higher OD values, with a maximal difference of ~0.3 OD units at ~15 h (Fig 1B). However, direct cell counting at OD_600_ = 0.65 showed 522,746 ± 3,400 cells μl^−1^ in the Dr^−^ culture and 393,250 ± 3,200 cells μl^−1^ in the Dr^+^ culture (n = 3, unpaired Student’s t-test, p < 0.001), indicating stronger light scattering by Dr^+^ cells.

The altered physical behavior of Dr^+^ suspensions was further confirmed in a vacuum filtration assay. Suspensions of AAEC191A Dr^−^ cells in 100 mM KNO_3_ (OD_600_ 0.325, 0.650, and 1.3) passed through a cellulose membrane within 5–13 ± 2 s, whereas corresponding Dr^+^ suspensions (OD_600_ 0.43, 0.85, and 1.7) required substantially longer filtration times (Fig 1C). The first dry point on the membrane appeared after 12 ± 4, 106 ± 18, and 462 ± 68 s, respectively, whereas complete disappearance of the liquid required 29 ± 5, 173 ± 26, and 593 ± 69 s. Comparable results were obtained in PBS at pH 5.7, 7.0, and 8.0, indicating no significant effect of pH on filtration behavior (S2 Fig). Filtrate analysis confirmed the absence of bacterial cells or released fimbriae, while both strains formed confluent layers on the membrane surface (Figs 1D,E and S3). Thus, prolonged filtration of Dr^+^ suspensions was associated with the presence of Dr fimbriae rather than release of extracellular material.

### TEM and LC-MS analysis of Dr envelope encoded by the model *E. coli* AAEC191A Dr^+^

Macroscopic observations and rheological measurements indicate that the Dr fimbrial envelope profoundly alters the physical behavior and surface properties of bacterial populations. To investigate the structural basis of these effects, we used transmission electron microscopy (TEM) together with a computational model informed by liquid chromatography–mass spectrometry (LC-MS) analysis. This framework provides a basis for interpreting the physicochemical properties and biological activities of Dr-fimbriated bacteria described in the following sections.

Available micrographs of Dr-fimbriated bacteria typically show only small regions of the cell surface near the membrane where the fimbriae are anchored [15]. Consequently, these images do not allow estimation of the overall volume of the Dr envelope, which is critical for understanding its contribution to bacterial surface properties. Similar to other proteinaceous adhesive systems, Dr fimbriae are highly hydrophilic and readily undergo structural disruption during TEM preparation and imaging. In TEM of the AAEC191A Dr^+^ strain, negative staining conditions were identified that stabilized the envelope, allowing visualization of its overall dimensions. Under these conditions, bacterial cells were surrounded by a pronounced electron-dense layer, corresponding to an envelope ~200–300 nm thick. Envelope density prevented resolution of individual filaments, but allowed reliable estimation of its relative size compared to the cell (S4A Fig). Bundled fimbrial structures, likely preparation artifacts, appeared only in partially disrupted envelopes (S4B Fig). TEM of AAEC191A Dr^−^ cells showed neither the electron-dense layer nor fibrillar bundles (S4C Fig).

#### LC–MS quantification of Dra proteins involved in Dr fimbriae assembly.

To estimate the number of fimbrial subunits in the Dr envelope, we quantified *dra* operon-encoded proteins using LC–MS. Overnight cultures of AAEC191A Dr^+^ grown in LB medium were analyzed for the major fimbrial subunit DraE, the outer membrane usher DraC, the periplasmic chaperone DraB, and the proposed apical subunit DraD. The mean number of proteins per cell (± SD) was: DraE – 242,000 ± 16,098; DraC – 1,630 ± 476; DraB – 168,000 ± 9,944; DraD – 60,300 ± 4,435. Because this total DraE includes cytoplasmic, periplasmic, and assembled fractions, we next determined the amount incorporated into surface fimbriae. The Dr envelope was isolated by incubating cells in PBS at 65 °C for 3 h, enabling quantitative release of surface fimbriae (S5A Fig). SDS-PAGE confirmed the exclusive presence of DraE, appearing as polymers at 25 °C and monomers after heat denaturation at 100 °C. Size-exclusion chromatography showed that DraE eluted in the column void volume, consistent with its polymeric nature (S5B Fig). LC–MS analysis of the purified fimbrial fraction revealed ~222,000 ± 18,300 DraE molecules per cell incorporated into the Dr envelope. This closely matches the total DraE content, indicating that most DraE in stationary-phase cells is assembled into fimbriae (unpaired two-tailed Student’s t-test, p > 0.05, n = 3). No DraC or DraB was detected in the fimbrial fraction, confirming the specificity of the isolation. DraD was also undetectable, suggesting very low abundance in the analyzed fimbrial fractions.

### Computational model of the Dr fimbrial envelope produced by the model strain AAEC191A Dr^+^

The experimentally determined stoichiometry of DraE and DraC proteins provided the basis for constructing a structural model of the Dr envelope. Assuming standard *E. coli* dimensions (radius ~500 nm and total length ~2000 nm) and that all DraC usher proteins participate in fimbrial assembly, the measured DraC copy number corresponds to approximately one fimbrial polymer per ~63 × 63 nm patch of the bacterial surface. Based on the DraE-to-DraC ratio, each polymer was estimated to contain approximately 140–150 DraE subunits.

**Fig 2:**
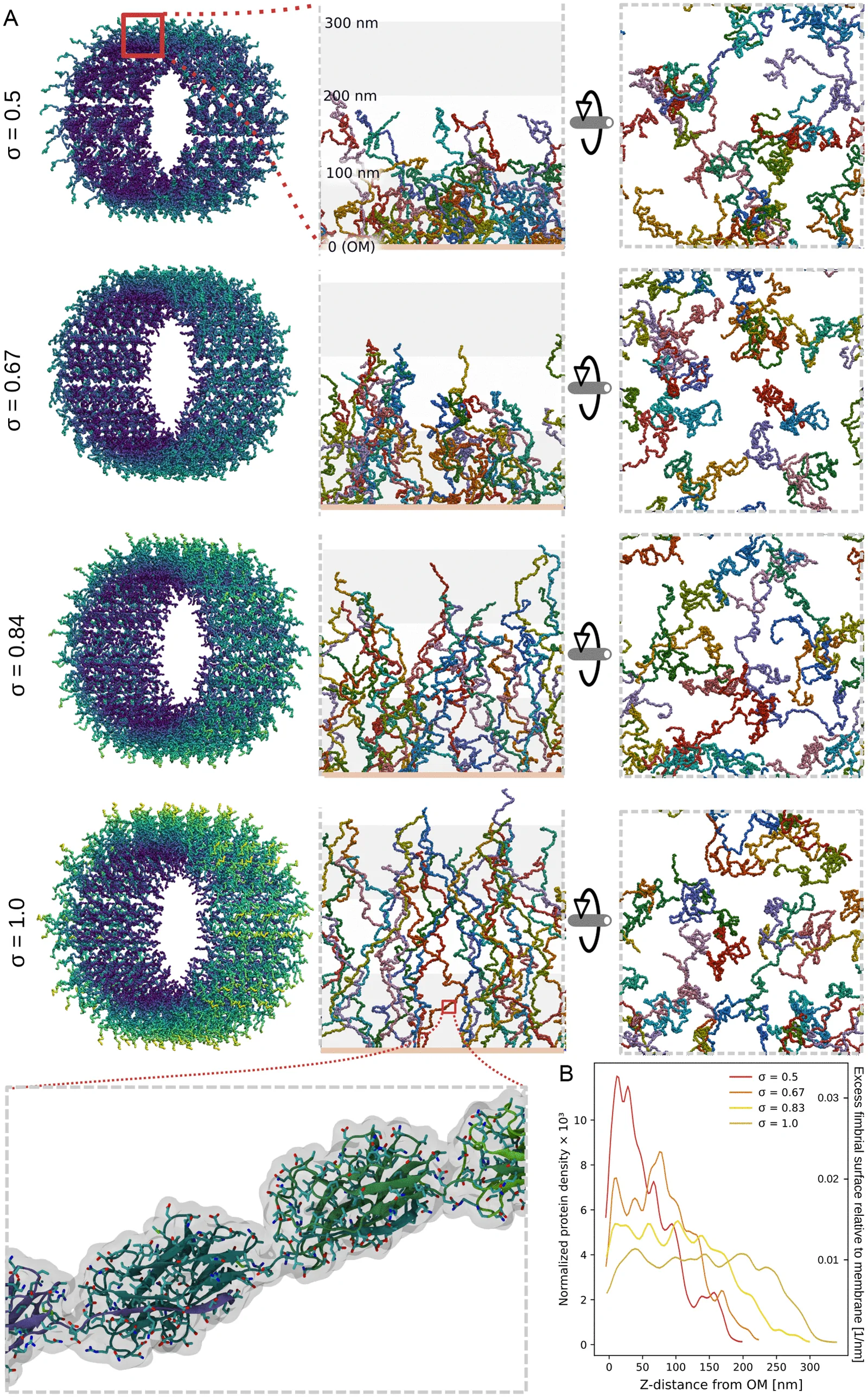
Model and structural properties of the Dr bacterial envelope. (A) Cylindrical sections of the envelope built upon a bacterial cell, assuming a standard *E. coli* radius of 500 nm, at different values of the σ parameter controlling the tendency of the fimbriae to grow and extend outwards; the color scale ranges radially from 500- to 900-nm distance from the cylinder axis (0–400 nm from the outer membrane). At σ = 1, all downward moves (i.e., adding a DraE unit whose center of mass is closer to the outer membrane than the previous one) are rejected; at σ = 0.5, a downward move would be rejected with a 50% chance. In central and rightmost panels, the corresponding side and top views of the 5x5 patch are shown, with individual fimbriae colored differently to show the degree of mixing and tangling. An atomistic structure is shown in the inset to highlight the scale of the assembly. (B) Protein density profiles as a function of the distance away from the bacterial outer membrane for different values of σ; the densities are normalized to integrate to 1. Excess fimbrial surface per nm is calculated as the fraction of the original outer membrane surface provided by DraE proteins contained in a 1-nm thick slice of the fimbrial envelope; this integrates to 2.7, the ratio of total fimbrial surface to the outer membrane surface area.

To generate realistic polymer geometry, molecular dynamics simulations were first performed for short DraE heptamers in explicit solvent. Donor-strand complemented DraE dimers extracted from these simulations were then used as building blocks to assemble ensembles of ~150-subunit fimbrial polymers (with very low model sensitivity to the assumed variance in polymer length, see S6 Fig). These polymers were built as XY-periodic 5 × 5 surface patches using Monte Carlo sampling to reproduce plausible spatial organization and steric constraints between neighboring fimbriae. The resulting patches were then geometrically transformed into cylindrical segments corresponding to the surface curvature of a bacterial cell, yielding a realistic representation of the Dr envelope (Fig 2A; S1 Video).

To explore how the spatial organization of fimbrial polymers may vary within the envelope, an “effective solubility” parameter σ was introduced into the model, with values of 0.5, 0.67, 0.84, and 1.0 (Fig 2A). This parameter controls the tendency of fimbrial polymers to extend away from the outer membrane and thereby imposes different packing dynamics of DraE subunits as a function of distance from the cell surface (Fig 2A,B). Physically, σ reflects both the electrostatic repulsion between negatively charged DraE subunits and the hydrophilic tendency to swell and avoid close contact between fimbriae, which together determine the degree to which fimbrial polymers extend into the surrounding medium. For σ = 0.5, the peak density of fimbrial proteins occurs within ca. 50 nm from the membrane surface, locally creating a thick mesh whose local adsorption-capable surface area more than doubles that of the outer membrane. Under these conditions, fimbrial polymers are densely packed, forming a compact envelope primarily localized within ~100 nm of the membrane surface. In contrast, for σ = 1.0 the density of DraE proteins becomes essentially constant over a thickness of 250 nm, reduced to one third to half of the thick mesh described above. In this configuration, the Dr envelope adopts a more extended, porous and relatively uniform structure. Intermediate σ values (0.67 and 0.84) produce correspondingly intermediate packing regimes. To verify that the obtained models accurately represent real structures, we compared the projected protein densities corresponding to different values of σ with two representative density slices of a TEM micrograph and found excellent agreement with the model corresponding to σ = 0.67 (S7 Fig). In this configuration, the Dr layer increases the effective cell diameter by approximately 40–60% relative to Dr-negative cells. Nevertheless, we speculate that alternative configurations might correspond to dynamic states of the Dr fimbrial envelope arising under varying physiological or environmental conditions experienced by the bacterial cell that might modulate effective forces between individual fimbriae.

### Physicochemical properties of the Dr fimbrial envelope

The Dr envelope of our AAEC191A Dr^+^ model consists of approximately 220,000–240,000 DraE protein subunits. Assuming an average accessible surface area (ASA) of 77 nm^2^ per DraE within the polymer, this corresponds to a total envelope surface area of ~18.5 µm^2^, compared to ~6 µm^2^ for a non-fimbriated cell. This surface is formed by flexible fimbrial fibers ~2 nm in diameter and up to ~600 nm long. Consequently, the chemical properties of the envelope surface are largely determined by DraE.

**Fig 3:**
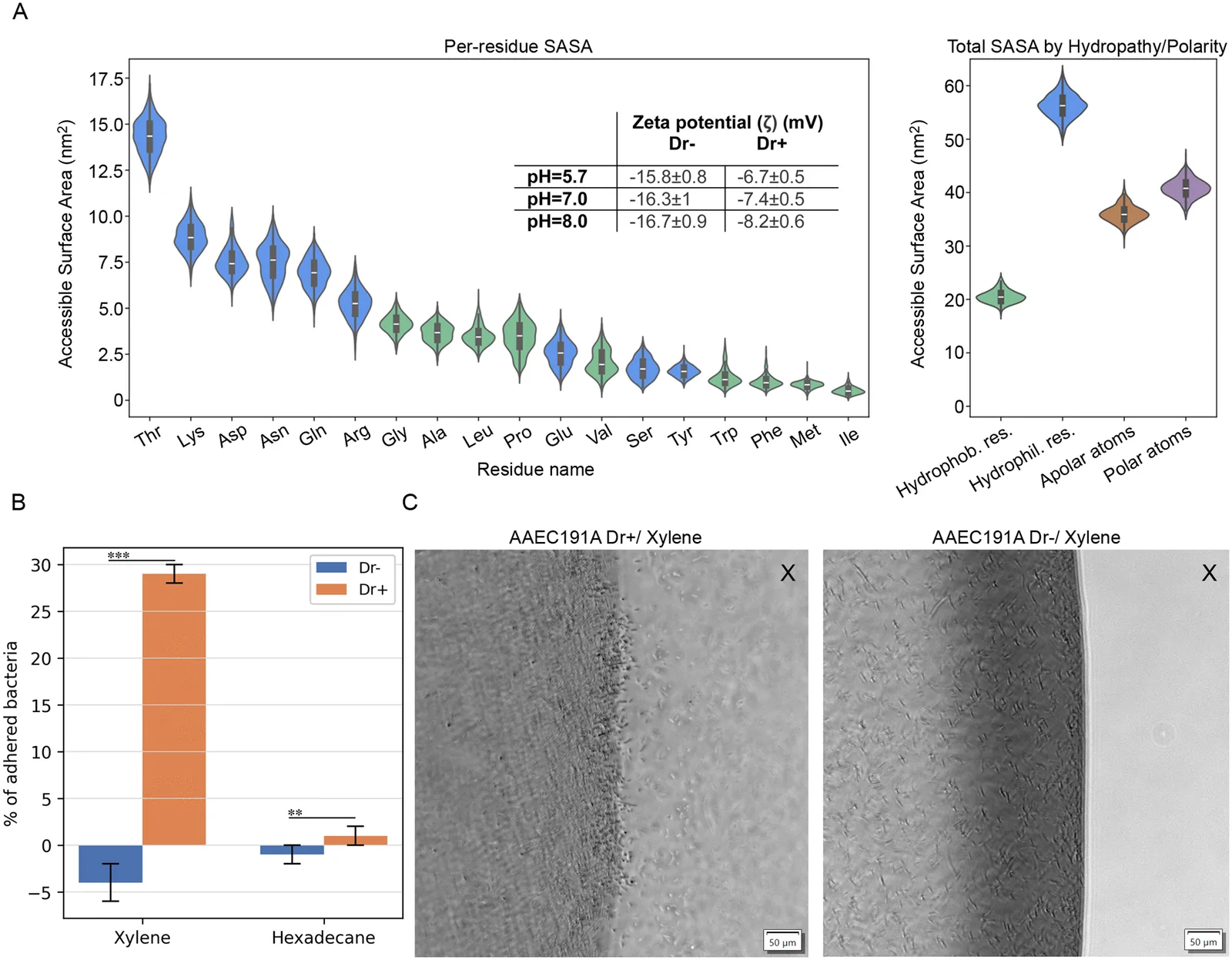
Dr fimbrial envelope enhances hydrophobic surface properties of *Escherichia coli.* (A) Surface hydrophobicity and hydrophilicity distribution of the DraE subunit calculated from solvent-accessible surface area (SASA) within the polymeric Dr fimbrial structure at atomistic resolution. Residues were classified as hydrophobic or hydrophilic as indicated in the left panel. Atoms were classified as polar or apolar using an absolute atomic charge cut-off of 0.25. The total solvent-accessible surface area per DraE subunit was 76.76 nm^2^. The inset shows zeta potential values of *E. coli* AAEC191A Dr^+^ and Dr^−^ cells measured in phosphate-buffered saline (PBS) at pH 5.7, 7.0, and 8.0. Data are presented as mean ± SD (n = 3). Statistical significance was evaluated using Welch’s corrected Student’s t-test. For all Dr^+^/Dr^−^ comparisons at each pH value, *** indicates p < 0.001. (B) Microbial adhesion to hydrocarbons (MATH) assay performed with AAEC191A Dr^+^ and Dr^−^ suspensions using xylene or hexadecane as the organic phase. The percentage of bacteria transferred from the aqueous phase to the organic phase was calculated relative to OD_570_ values of control suspensions without organic solvent. Bacterial suspensions were normalized to equivalent cell numbers in PBS at pH 7.0. Data represent mean ± SD (n = 3), statistical significance was evaluated using non-parametric Kruskal-Wallis test (** p < 0.01, *** p < 0.001). (C) Representative phase-contrast images of the interface between xylene (marked X) and the aqueous phase containing AAEC191A Dr^+^ or Dr^−^ cells. The same bacterial suspensions as in panel (B) were used. Images were acquired 15 min after phase equilibration.

Analysis of the solvent-accessible surface area of DraE at the level of individual amino acid residues shows similar fractions of apolar (absolute atomic charge |Q| < 0.25) and polar atoms (|Q| > 0.25) (Fig 3A). This distribution reflects the high abundance of surface-exposed Thr and Lys residues, whose side chains contain extended apolar fragments. As a result, the Dr envelope is amphipathic, with a large and chemically heterogeneous surface. Understanding its physicochemical properties therefore requires multiple complementary approaches, each revealing different aspects of its surface organization and contribution to bacterial behavior.

#### Hydrophobicity of the model Dr-fimbriated bacteria assessed by MATH and contact angle measurements.

In the microbial adhesion to hydrocarbons (MATH) assay, bacteria remain suspended in the aqueous phase, preserving the structure and function of surface organelles. When xylene was used as the organic phase, the percentage of adhesion to hydrocarbons was −4.69 ± 1.2% for AAEC191A Dr^−^ and 29.27 ± 0.45% for AAEC191A Dr^+^, indicating substantially higher surface hydrophobicity in Dr-fimbriated bacteria (Fig 3B). Phase-contrast microscopy showed that Dr^+^ cells accumulated at the xylene–PBS interface and partitioned into the organic phase (Fig 3C). In contrast, AAEC191A Dr^−^ cells did not show visible accumulation at the interface, and no bacteria were observed within the organic phase. Using hexadecane, neither strain showed measurable adhesion.

In the contact angle (CA) method, the surface hydrophobicity of bacteria is determined using a semi-dried bacterial layer. Water CAs below 25° indicate hydrophilic surfaces, while angles above 25° indicate hydrophobic surfaces. CA measurements also allow quantitative evaluation through the free energy of interaction between bacterial surfaces in water (ΔG_BWB_), where ΔG_BWB_ < 0 denotes hydrophobic and ΔG_BWB_ > 0 hydrophilic surfaces. Measured water CAs were 31.2 ± 1.99° for Dr^−^ and 28.0 ± 1.44° for Dr^+^, with corresponding ΔG_BWB_ values of 28.9 and 20.5 mJ m^−2^ (S1 Table in S1 File), showing the same qualitative trend.

The two methods differed: the MATH assay revealed markedly higher hydrophobicity of Dr-fimbriated bacteria, while CA measurements showed only minor differences. This indicates that the observed increase in hydrophobicity is detectable only when the three-dimensional structure of the Dr fimbrial envelope is preserved.

#### Surface charge of the model Dr-fimbriated bacteria assessed by zeta potential measurements.

Zeta potential of AAEC191A Dr^−^ and Dr^+^ strains was measured in PBS at pH 5.7, 7.0, and 8.0, matching conditions used in bacterial adhesion assays. Both strains showed only minor changes across the pH range. For Dr^+^ bacteria, zeta potentials were −6.7, −7.4, and −8.2 mV at pH 5.7, 7.0, and 8.0, respectively, whereas Dr^−^ cells exhibited more negative values of −15.8, −16.3, and −16.7 mV (Fig 3A-inset). Thus, the Dr envelope reduces the negative zeta potential by ~50–60% compared with non-fimbriated cells. Notably, the purified Dr fimbrial fraction had a zeta potential of ~−8.5 mV, similar to intact Dr^+^ cells, indicating that the Dr envelope largely determines the electrostatic properties of the bacterial surface. The less negative zeta potential of Dr^+^ cells may reduce electrostatic repulsion, promoting interactions with other cells and abiotic surfaces.

#### Probing the ultrastructure of the model Dr fimbrial envelope by glutaraldehyde crosslinking.

To investigate the ultrastructural organization and packing density of the Dr fimbrial envelope, we assessed intercellular crosslinking of Dr^+^ bacteria with glutaraldehyde, which reacts with ε-amino groups of lysine to form covalent bridges. According to our model, each AAEC191A Dr^+^ cell contains ~240,000 DraE subunits assembled into ~1,600 fimbriae, with each DraE carrying eight lysines, providing nearly two million potential crosslinking sites. This exceptionally high density of reactive groups is expected to favor intermolecular crosslinking between neighboring cells. To test this, bacterial suspensions were incubated with a gradient of glutaraldehyde concentrations (0.01–2%) and the formation of aggregates was monitored.

**Fig 4:**
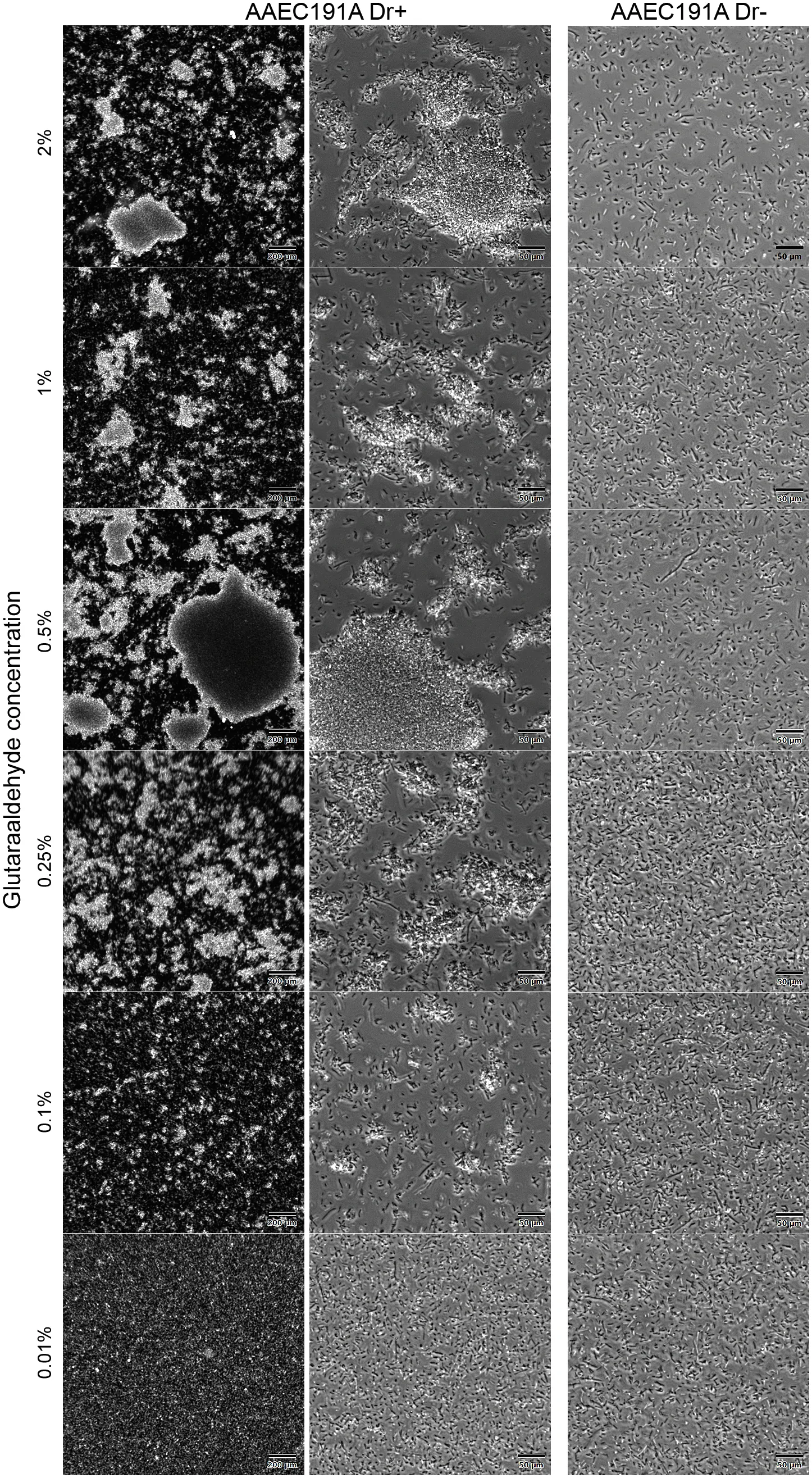
Glutaraldehyde-induced crosslinking reveals the extended reactive architecture of the Dr fimbrial envelope. Representative phase-contrast images of *E. coli* AAEC191A Dr^+^ and Dr^−^ cells suspended in PBS (pH 7.5, OD_600_ = 1.0) and incubated for 15 min at 4 °C with increasing concentrations of glutaraldehyde (0.01%, 0.1%, 0.25%, 0.5%, 1%, and 2%). In the Dr^+^ column, the left image series shows the overall extent and size distribution of bacterial aggregates formed at each glutaraldehyde concentration (scale bar, 200 µm), whereas the right image series presents higher-magnification views of aggregate ultrastructure and local packing density (scale bar, 50 µm). In the Dr^−^ column, only high-magnification images are shown (scale bar, 50 µm), as no detectable aggregate formation occurred at any glutaraldehyde concentration tested.

Dr^+^ cells formed aggregates across a broad concentration range, down to 0.1%, with maximal aggregation at 0.5%. Higher concentrations (1–2%) reduced aggregate formation, likely due to intrafimbrial or interfimbrial crosslinking within individual cells. Aggregate sizes ranged from small clusters to massive structures up to ~1200 µm, with dense packing observed by phase-contrast microscopy (Fig 4). Aggregate growth was enhanced by the increasing total surface area, providing more sites for intermolecular crosslinking, a “surface amplification” effect. In contrast, Dr^−^ cells showed no detectable aggregation (Fig 4).

These findings highlight that the Dr fimbrial envelope forms an extended, highly reactive surface layer whose three-dimensional architecture is critical for efficient intercellular crosslinking, revealing the remarkable spatial reach and connectivity of the fimbrial network surrounding the bacterial cell.

### Adhesion of the model Dr-fimbriated bacteria to abiotic surfaces under static conditions

**Fig 5:**
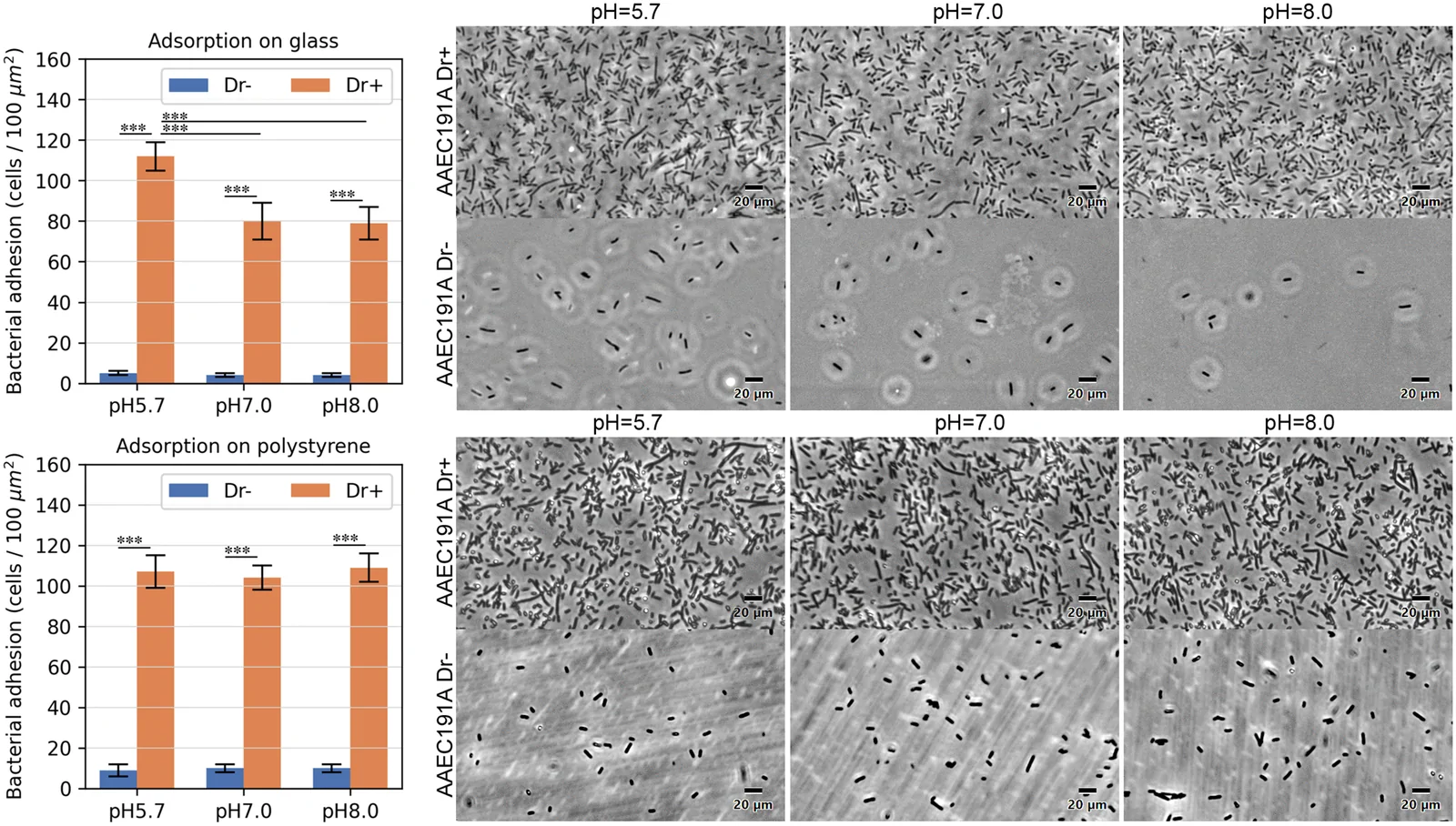
Dr fimbrial envelope promotes strong adhesion of *Escherichia coli* to hydrophilic and hydrophobic abiotic surfaces under static conditions. Adhesion of AAEC191A Dr^+^ and Dr^−^ cells suspended in phosphate-buffered saline (PBS) at pH 5.7, 7.0, or 8.0 (OD_600_ = 0.7) to glass and polystyrene surfaces under static conditions. The graphs show the number of adherent bacteria quantified after 15 min incubation followed by gentle washing with the corresponding buffer. Adhesion is expressed as the number of surface-associated cells per 100 µm^2^. Data represent mean ± SD (n = 3), statistical significance was evaluated using non-parametric Kruskal-Wallis test (*** p < 0.001). Representative phase-contrast images show bacterial adhesion patterns on glass and polystyrene. Scale bars, 20 µm.

Given that the Dr fimbrial envelope forms an amphipathic and extended surface layer capable of nonspecific interactions, we examined adhesion of AAEC191A Dr^+^ bacteria to abiotic surfaces under static conditions. Glass and polystyrene, with water CAs of ~25° and ~85°, served as hydrophilic and hydrophobic substrates. Bacterial suspensions (OD_600_ = 0.7) in PBS at pH 5.7, 7.0, or 8.0 were deposited onto these surfaces, incubated, and non-adherent cells were gently washed off. Surface-associated cells were quantified by phase-contrast microscopy. Dr^+^ bacteria adhered strongly to both surfaces, with ~80–110 cells/100 µm^2^ on glass and ~105 cells/100 µm^2^ on polystyrene. Adhesion to polystyrene was pH-independent, whereas adhesion to glass decreased by ~30% at pH 7.0 and 8.0 compared to pH 5.7. In contrast, Dr^−^ cells showed minimal adhesion, ~ 5 cells/100 µm^2^ on glass and ~8 cells/100 µm^2^ on polystyrene, independent of pH (Fig 5).

### Stability of the model Dr-fimbriated bacterial adhesion to glass and polystyrene under receding-meniscus capillary forces

To estimate the strength of bacterial adhesion to abiotic surfaces, we examined the susceptibility of surface-attached cells to detachment by a receding liquid meniscus. Suspensions of AAEC191A Dr^+^ and Dr^−^ bacteria in PBS (pH 7.0, OD_600_ = 0.5) were deposited onto glass and polystyrene surfaces and subsequently covered with a coverslip, forming a liquid layer approximately 10 ± 2 µm thick. Controlled evaporation of water at the edge of the coverslip induced a gradual recession of the liquid meniscus across the surface. Bacteria adhered to the substrate were thus exposed to forces generated by the moving meniscus, which effectively acted as a mechanical scraper removing weakly attached cells.

**Fig 6:**
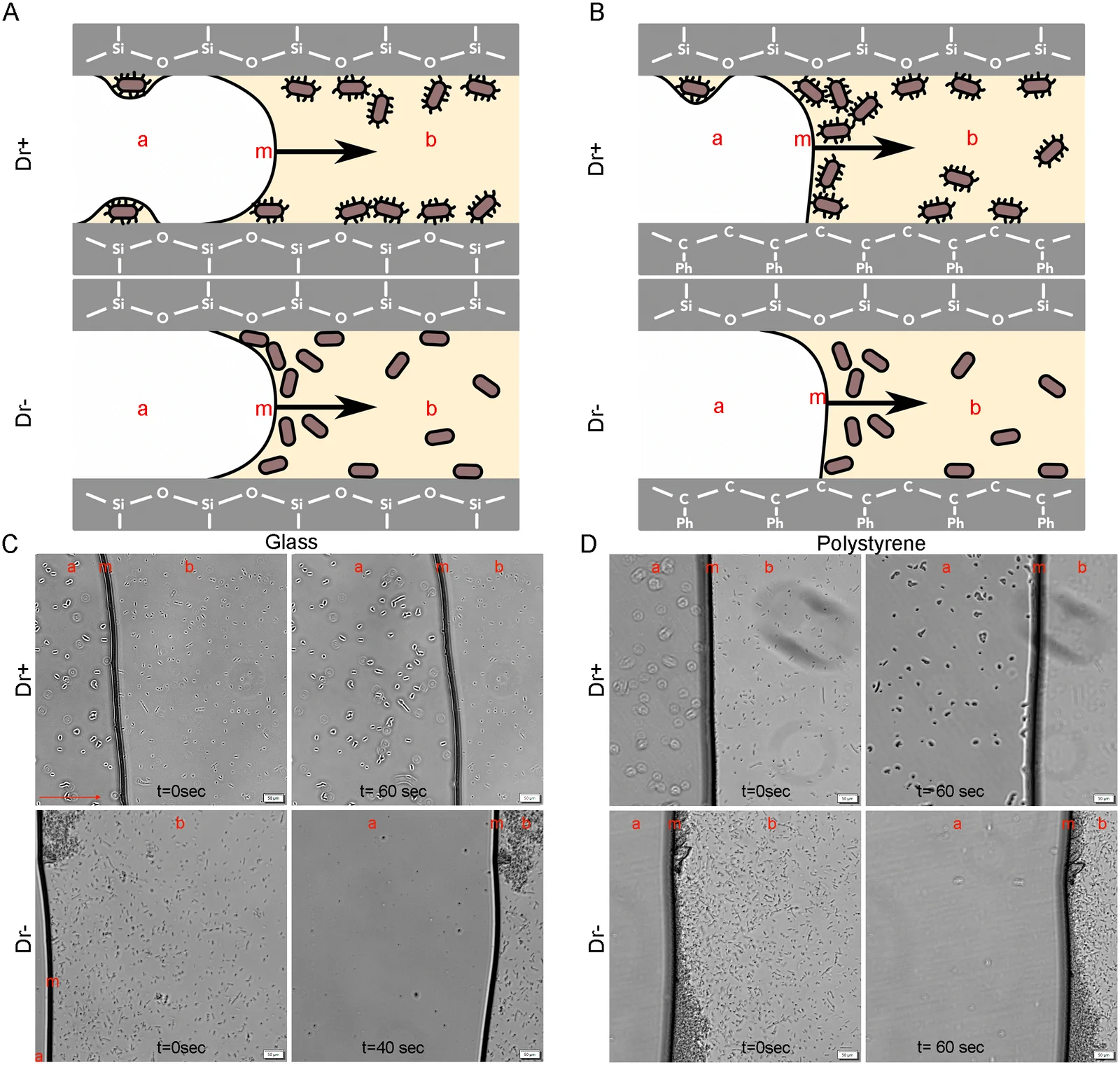
Dr fimbrial envelope stabilizes bacterial adhesion to glass during exposure to receding-meniscus forces. (A, B) Schematic representation of bacterial behavior during passage of a receding liquid meniscus on glass (A) and untreated polystyrene (B) surfaces. Region (a) indicates the area exposed to air after meniscus passage, region (b) the liquid-filled region ahead of the moving meniscus, and (m) the meniscus position. Black arrows indicate the direction of meniscus movement. (C, D) Representative phase-contrast images showing meniscus-driven displacement of *E. coli* AAEC191A Dr^+^ and Dr^−^ cells on glass (C) and untreated polystyrene (D). Bacterial suspensions in phosphate-buffered saline (PBS, pH 7.0, OD_600_ = 0.5) were confined beneath a coverslip, forming a liquid layer of 10 ± 2 µm thickness. Images were recorded during evaporation-induced meniscus recession at 25 °C. Time after onset of recording is indicated in each panel. Scale bars, 50 µm. Meniscus motion was recorded at 5 frames per second for 1 min.

On glass, the meniscus detached 98 ± 2% of Dr^−^ cells, moving at ~14 ± 2 µm s^−1^. In contrast, 67 ± 3% of Dr^+^ cells remained attached, and meniscus motion became discontinuous, slowing to ~3.9 ± 2 µm s^−1^, with pronounced slowing observed where surface-bound bacteria were present (Fig 6A,C and S2 Video). Different behavior was observed on the hydrophobic polystyrene surface. Dr^−^ bacteria were completely removed by the receding meniscus, which moved at a velocity of approximately 7 ± 1 µm s^−1^. For Dr^+^ cells, the capillary force also detached essentially 100% of bacteria from the polystyrene surface, with the meniscus moving uniformly at approximately 3.4 ± 1.5 µm s^−1^. However, detached Dr^+^ bacteria were immediately transported within the meniscus and accumulated at the wetting arm contacting the upper glass coverslip, where they subsequently adhered with high efficiency. As a consequence, a pronounced accumulation of bacteria was observed on the glass surface behind the receding meniscus in regions where the liquid film had already evaporated (Fig 6B,D and S3 Video).

Similar results were obtained for bacterial suspensions prepared in PBS buffers at pH 5.7 and 8.0. Together, these observations indicate that adhesion of Dr-fimbriated bacteria to the hydrophilic glass surface is substantially more stable under the applied experimental conditions than adhesion to the hydrophobic polystyrene surface. Using a physicochemical framework describing capillary forces generated during liquid recession in thin liquid films, the characteristic force acting on a bacterium with an effective radius of approximately 0.5 µm can be estimated to be on the order of several tens of nanonewtons, depending on the local geometry of the receding meniscus (see S2 Rawgel). The observation that a substantial fraction of Dr^+^ bacteria remained attached to the glass surface after passage of the meniscus indicates that the adhesion strength of Dr-fimbriated cells to glass is comparable to this force scale. The pronounced reduction in meniscus velocity observed on surfaces populated with Dr^+^ bacteria is consistent with enhanced pinning of the three-phase contact line by strongly adhered cells, which increases the energy required for the receding interface to advance across the surface.

### Adhesion of the model Dr-fimbriated bacteria to glass and polystyrene under laminar shear flow

**Fig 7:**
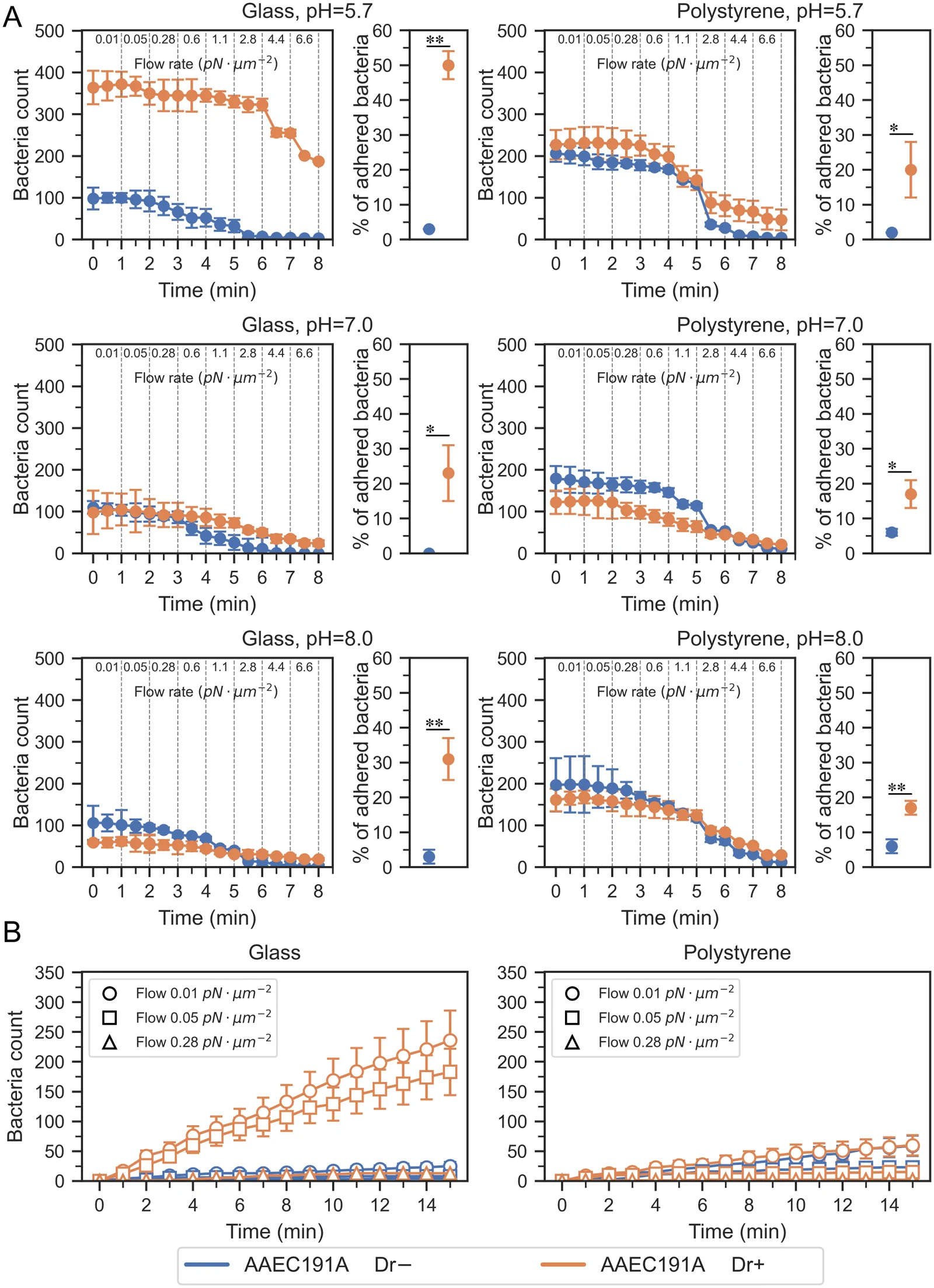
Influence of the Dr fimbrial envelope on bacterial adhesion to glass and polystyrene under laminar shear flow at different pH values. (A) Cell number-normalized suspensions of *E. coli* AAEC191A Dr^−^ and Dr^+^ bacteria in PBS at pH 5.7, 7.0, or 8.0 were introduced into flow chambers assembled on glass or untreated polystyrene surfaces and incubated for 10 min under static conditions to allow initial adhesion. Flow was then applied in sequential 1-min intervals generating increasing shear stress (0.01–6.6 pN µm^−2^). The graphs show the number of adherent bacteria during stepwise increase of shear stress. The right panels indicate the percentage of initially attached bacteria remaining on the surface at the end of the flow experiment. (B) Accumulation of cell number-normalized Dr^−^ and Dr^+^ bacterial suspensions in PBS at pH 5.7 during 15 min of continuous flow at constant shear stress values of 0.01, 0.05, or 0.28 pN µm^−2^ on glass and untreated polystyrene surfaces. Error bars in (A) and (B) represent 95% confidence intervals calculated from three independent biological experiments. Statistical significance was evaluated using non-parametric Kruskal-Wallis test (* p < 0.05, ** p < 0.01).

The capillary forces generated by a receding meniscus, reaching tens of nanonewtons, far exceed the hydrodynamic drag forces acting on bacteria under laminar flow, which in physiological environments such as the urinary tract are typically only several to tens of piconewtons. To assess adhesion under more physiologically relevant shear, AAEC191A Dr^+^ and Dr^−^ strains were analyzed on glass and polystyrene in a flow chamber under laminar shear stress from 0.01 to 6.6 pN µm^−2^. Bacteria suspended in PBS at pH 5.7, 7.0, or 8.0 were first allowed to attach under no-flow conditions, and then subjected to gradually increasing shear while the number of surface-adhered cells was monitored (Fig 7A; see also Video 6 and Video 7 in the Zenodo repository [53]).

On glass at pH 5.7, Dr^+^ bacteria adhered efficiently during the no-flow phase (364 ± 40 cells), with slight additional accumulation at low shear (0.01–0.05 pN µm^−2^). Detachment was minimal up to 2.8 pN µm^−2^, with 50 ± 4% remaining at 6.6 pN µm^−2^. In contrast, Dr^−^ cells adhered poorly (98 ± 26), with substantial detachment already at 0.28 pN µm^−2^ and only 3% remaining at 6.6 pN µm^−2^. At pH 7.0 and 8.0, initial Dr^+^ adhesion was lower (98 ± 58 and 59 ± 3 cells), with no increase in bacterial number at 0.01 pN µm^−2^, detachment began around 1.1 pN µm^−2^, and 23 ± 8% and 31 ± 6% of cells remained at 6.6 pN µm^−2^. For the non-fimbriated strain, the initial number of adhered bacteria was similar to that of Dr^+^ cells at these pH values, but detachment began already at 0.05 pN µm^−2^, leaving only 0 ± 0% and 3 ± 2% of cells at the end of the experiment for pH 7.0 and 8.0, respectively. On polystyrene, initial attachment was similar for both strains across pH 5.7–8.0. Despite this, Dr^+^ bacteria resisted detachment more effectively: at 6.6 pN µm^−2^, 20 ± 8%, 17 ± 4%, and 17 ± 2% of Dr^+^ cells remained at pH 5.7, 7.0, and 8.0, respectively, compared with only 2 ± 0%, 6 ± 1%, and 6 ± 2% for Dr^−^ cells.

Time-dependent accumulation under continuous flow at low shear (0.01, 0.05, 0.28 pN µm^−2^, pH 5.7) showed that Dr^+^ cells on glass accumulated to 236 ± 50, 183 ± 39, and 13 ± 4 cells, whereas Dr^−^ cells reached only 25 ± 7, 8 ± 3, and 4 ± 2. On polystyrene, accumulation was observed only at the lowest shear (0.01 pN µm^−2^), with 60 ± 17 Dr^+^ and 59 ± 17 Dr^−^ cells (Fig 7B; see also Video 8 and Video 9 in the Zenodo repository [53]).

Overall, the Dr fimbrial envelope markedly enhances adhesion stability under laminar flow, particularly on hydrophilic glass, where cells also accumulate under low shear. Adhesion to glass shows clear pH dependence, whereas polystyrene binding remains largely unaffected.

### Adhesion of the model Dr-fimbriated bacteria to kidney stone minerals: hydroxyapatite and calcium oxalate

The Dr fimbrial envelope forms a strongly hydrophilic surface due to the high density of polar residues exposed on DraE subunits, which is expected to promote interactions with polar mineral phases present in urinary tract stones. To model potential interactions of uropathogenic *E. coli* with kidney stone components, adhesion of AAEC191A Dr^+^ and Dr^−^ strains to hydroxyapatite (HA) and CaOx was examined.

**Fig 8:**
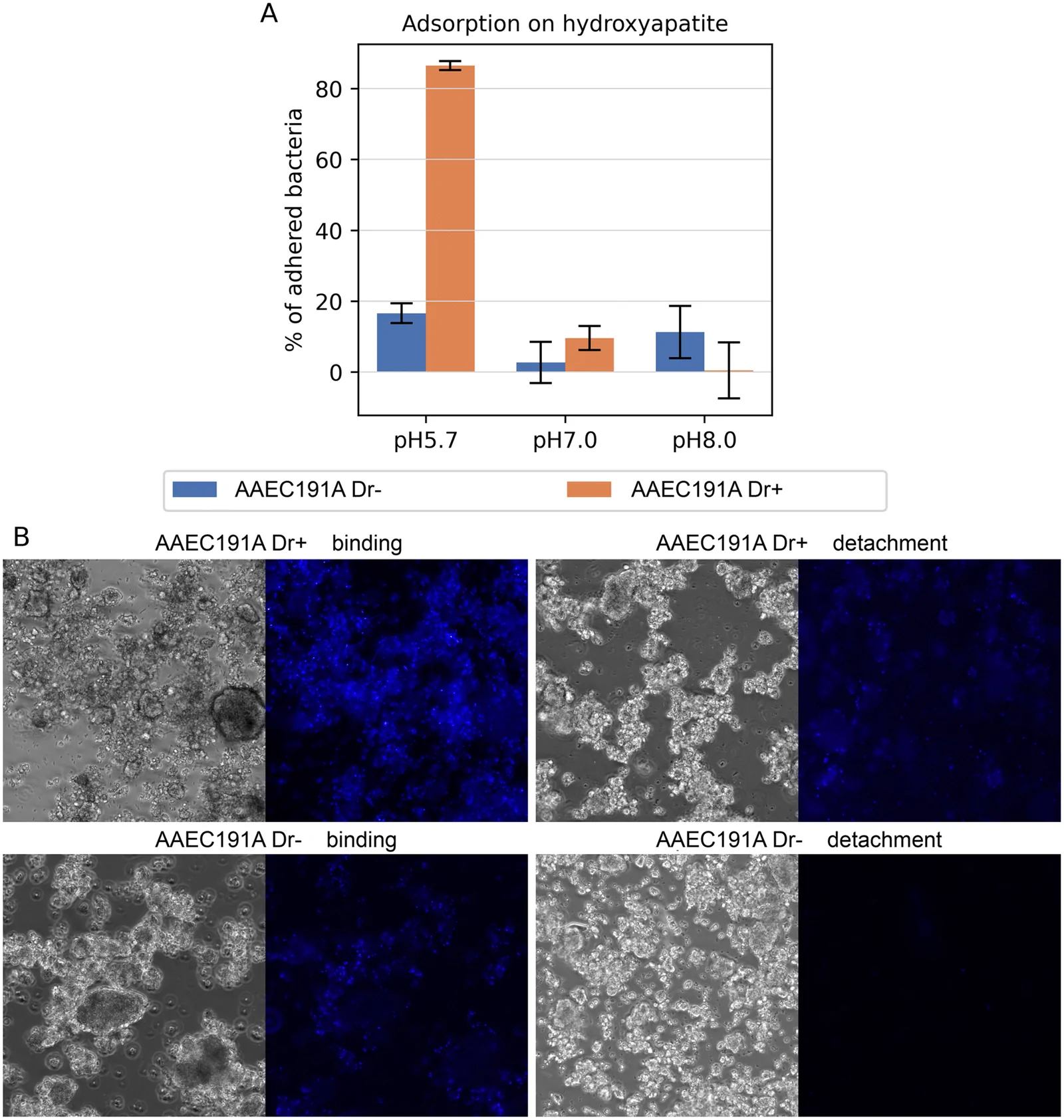
Influence of the Dr fimbrial envelope on bacterial adhesion to hydroxyapatite as a function of pH. (A) Percentage of *E. coli* AAEC191A Dr^−^ and Dr^+^ bacteria transferred from suspension to HA, calculated from the decrease in OD_600_ relative to control suspensions without HA. Bacterial suspensions normalized for cell number were prepared in PBS at pH 5.7, 7.0, or 8.0. Data represent mean ± SD (n = 3), statistical analysis was performed using the non-parametric Kruskal-Wallis test. For both strains, differences between adherence values on HA at all analyzed pH conditions were highly significant (p < 0.001), except for the Dr^−^ strain between pH 5.7 and pH 8.0, where p < 0.01 was obtained. (B) Representative phase-contrast images of HA crystals and corresponding fluorescence images of DAPI-stained bacteria attached to the crystal surface at pH 5.7. Binding shows samples after DAPI staining followed by a single gentle wash to remove excess fluorophore. Detachment shows bacteria remaining associated with HA after three washing cycles combined with vigorous pipetting to assess binding strength. Corresponding images obtained at pH 7.0 and 8.0 are shown in S8 Fig.

**Fig 9:**
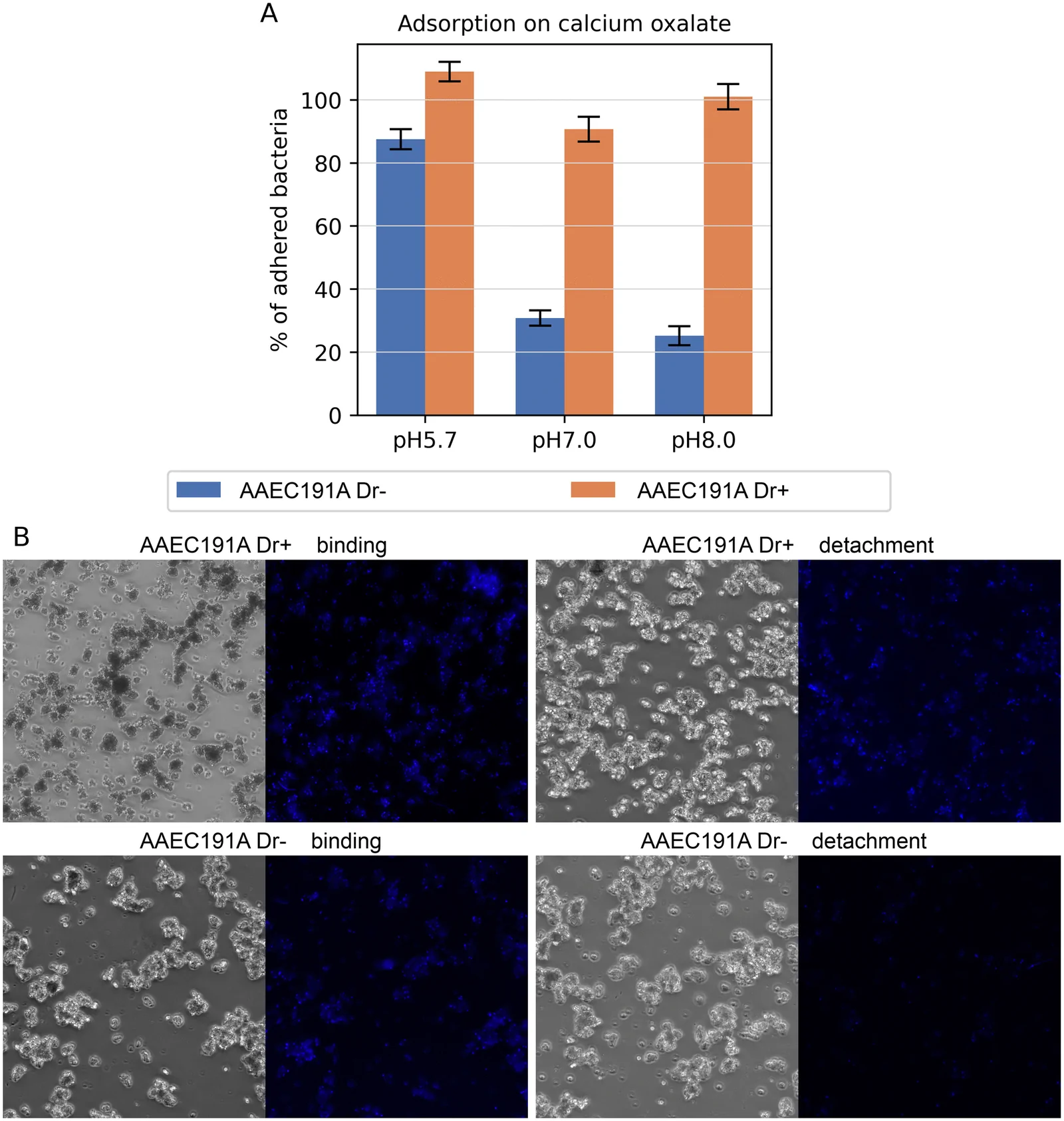
Influence of the Dr fimbrial envelope on bacterial adhesion to calcium oxalate as a function of pH. (A) Percentage of *E. coli* AAEC191A Dr^−^ and Dr^+^ bacteria transferred from suspension to CaOx monohydrate, calculated from the decrease in OD_600_ relative to control suspensions without CaOx. Bacterial suspensions normalized for cell number were prepared in PBS at pH 5.7, 7.0, or 8.0. Data represent mean ± SD (n = 3), statistical analysis was performed using the non-parametric Kruskal-Wallis test. For both strains, adherence on CaOx differed significantly across all analyzed pH conditions (p < 0.001). (B) Representative phase-contrast images of CaOx crystals and corresponding fluorescence images of DAPI-stained bacteria attached to the crystal surface at pH 5.7. Binding shows samples after DAPI staining followed by a single gentle wash to remove excess fluorophore. Detachment shows bacteria remaining associated with CaOx after three washing cycles combined with vigorous pipetting to assess binding strength. Corresponding images obtained at pH 7.0 and 8.0 are shown in S9 Fig.

Adhesion was quantified as the percentage decrease in OD_600_ of the initial bacterial suspension after mixing with a defined amount of HA or CaOx (Figs 8A, 9A), and qualitatively visualized by DAPI staining of bacteria bound to mineral sediments (Figs 8B, 9B, and S8, S9). To assess binding strength, sediments with attached bacteria were washed three times with fresh buffer by intensive pipetting and reanalyzed microscopically. At pH 5.7, 87 ± 2% of Dr-fimbriated bacteria were transferred from suspension to HA, consistent with intense fluorescence of DAPI-stained cells attached to HA crystals (Fig 8). At pH 7.0 and 8.0, adhesion decreased sharply to 10 ± 4% and 1 ± 8%, respectively (Fig 8A). In contrast, the non-fimbriated strain showed only weak HA association across the tested pH range, with 17 ± 3%, 3 ± 6%, and 11 ± 8% binding at pH 5.7, 7.0, and 8.0, respectively. Washing revealed progressive loss of permanently associated Dr^+^ cells with increasing pH, whereas almost no Dr^−^ cells remained attached after washing (Figs 8B and S8).

A different pattern was observed for CaOx. Dr-fimbriated bacteria showed nearly complete binding at all tested pH values (Fig 9A), although washing revealed a marked reduction in binding strength with increasing pH, with almost no Dr^+^ cells remaining attached at pH 8.0 (Figs 9B and S9). The non-fimbriated strain also interacted with CaOx, but less efficiently, with binding levels of 88 ± 3%, 31 ± 3%, and 25 ± 3% at pH 5.7, 7.0, and 8.0, respectively. In all cases, washing nearly completely removed Dr^−^ bacteria from CaOx sediments (Figs 9B and S9).

Overall, the Dr fimbrial envelope markedly enhances bacterial association with mineral phases relevant to kidney stone formation, particularly under mildly acidic conditions.

### Growth of the model Dr-fimbriated and non-fimbriated bacteria in artificial urine containing hydroxyapatite and calcium oxalate

Given the strong interaction of Dr-fimbriated bacteria with HA and CaOx observed in the adhesion assays, we next examined whether the presence of these minerals influences bacterial growth in artificial urine (AU).

The experiment was performed in AU adjusted to pH 5.7. At pH 7.0 and 8.0, intensive spontaneous precipitation of various crystalline phases, mainly carbonates and phosphates, was observed, which made it difficult to attribute the observed effects of bacterial growth to specific mineral types. In contrast, at pH 5.7, also within the physiological range of human urine, the spontaneous formation of crystals was strongly reduced. This allowed the establishment of a controlled model of kidney stone components by supplementing AU with defined crystals of CaOx or HA. As a potential carbon source, AU was supplemented with 0.2% L-lactate, a metabolite commonly present in the urinary tract [6]. During 24 h of cultivation under these conditions, the optical density of the bacterial cultures increased from an initial OD_600_ of 0.025 to approximately 0.1.

After 2 h of cultivation, approximately 80% of Dr-fimbriated bacteria were associated with CaOx crystals. The binding exhibited a highly specific spatial pattern dominated by interactions occurring through the bacterial cell pole, resulting in a characteristic radial arrangement of bacteria surrounding the central CaOx crystal. The number of attached Dr^+^ cells was sufficiently large that their cumulative motility caused visible oscillations and occasional displacement of CaOx crystals along the bottom surface of the cultivation chamber (S4 Video). In contrast, the Dr^−^ strain did not display any specific interactions with CaOx and remained uniformly distributed throughout the medium. Interestingly, Dr-fimbriated bacteria did not exhibit detectable interactions with HA crystals under these conditions, similarly to the non-fimbriated strain (S5 Video).

**Fig 10:**
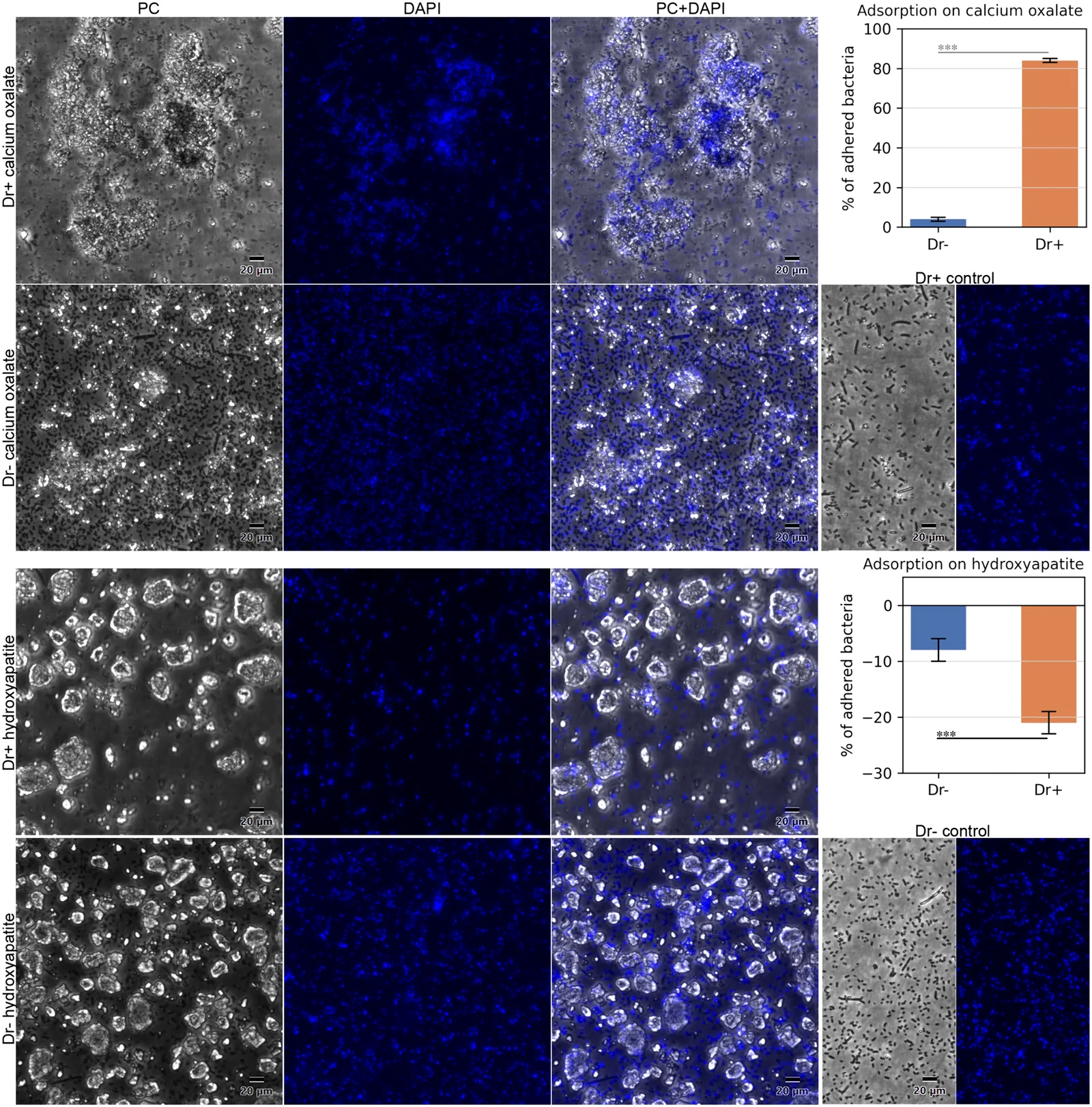
Preferential accumulation of Dr^+^ bacteria on calcium oxalate during growth in artificial urine. Representative phase-contrast (PC), fluorescence (DAPI), and merged (PC+DAPI) images showing *E. coli* AAEC191A Dr^+^ and Dr^−^ bacteria after 24 h growth in AU (pH 5.7), with control samples consisting of AU alone (without HA or CaOx), and experimental samples containing HA or CaOx at a final concentration of 0.05% (w/v). Overnight cultures were diluted in AU to OD_600_ = 0.025 and incubated statically at 37 °C in 24-well polystyrene plates. After incubation, bacteria associated with crystal surfaces were stained with DAPI, then planktonic cells were gently removed and crystals remaining at the bottom of the wells were rinsed once with AU. Phase-contrast images show crystal morphology, fluorescence images show DAPI-labeled bacteria, and merged images present the spatial relationship between bacteria and crystal surfaces. Scale bars, 20 µm. The graphs on the right show the percentage of bacteria transferred from suspension to HA or CaOx during incubation in AU under agitation, calculated from the decrease in OD_600_ relative to control suspensions without crystals. Data represent mean ± SD, statistical significance was evaluated using non-parametric Kruskal-Wallis test (*** p < 0.001).

After 24 h of cultivation, bacteria were stained with DAPI, then planktonic cells were removed by washing, and those associated with the polystyrene bottom surface and with CaOx or HA crystals were analyzed using fluorescence and phase-contrast microscopy. In cultures supplemented with CaOx, very large aggregates of Dr-fimbriated bacteria associated with the crystal surfaces were observed (Figs 10 and S10). In contrast, the control Dr^−^ strain did not display an increased level of association with CaOx compared with the number of cells attached to the polystyrene surface of the culture vessel. Furthermore, for both strains grown in AU in the presence or absence of CaOx or HA crystals, no bacterial clusters indicative of biofilm formation were detected on the bottoms of the culture wells (Fig 10).

Notably, even after 24 h of cultivation, no increased association of Dr-fimbriated bacteria with HA crystals was observed relative to the background level, similarly to the behavior of the Dr^−^ strain (Figs 10 and S11). The absence of detectable binding of Dr-fimbriated bacteria to HA crystals in AU at pH 5.7 was unexpected in the context of the strong interaction observed previously in PBS at the same pH (Fig 8). To verify this observation, an additional experiment was performed in which Dr^+^ and Dr^−^ bacteria suspended in AU (pH 5.7) were mixed with HA or CaOx crystals under agitation, and the decrease in OD_600_ of the supernatant was monitored as a measure of bacterial binding to the mineral phase. In mixtures containing CaOx, approximately 82 ± 3% of Dr-fimbriated bacteria and about 7 ± 2% of non-fimbriated bacteria were removed from suspension due to binding to the crystals. In contrast, no detectable binding of either bacterial strain to HA was observed in the analogous experiment (Fig 10). These results indicate that the lack of interaction between bacteria and HA in AU, compared with PBS at the same pH, is likely caused by components present in AU. Chemical analysis showed that citrate ions present in AU cause gradual decomposition of HA crystals (S12 Fig).

Together, these results confirm the strong and selective interaction of Dr-fimbriated bacteria with CaOx and indicate that components of AU can profoundly modify interactions with HA.

### Phage susceptibility reveals size-dependent shielding by the model Dr fimbrial envelope

Autoaggregation of bacterial cells is typically mediated by specific proteins exposed on the cell surface and leads to visible sedimentation under stationary conditions. The *E. coli* BL21(DE3) and JM101 strains exhibited strong autoaggregation, resulting in a 50% reduction in optical density after approximately 2 and 6 h, respectively (S13 Fig). In contrast, AAEC191A showed no detectable sedimentation, and its optical density remained unchanged even after 24 h. Introduction of Dr fimbriae into BL21(DE3) and JM101 completely abolished autoaggregation, causing both strains to behave similarly to AAEC191A (S13 Fig). These observations indicate that the Dr envelope physically masks surface determinants responsible for cell–cell interactions and suggest that, beyond promoting adhesion to biotic and abiotic surfaces, it may also function as a steric barrier limiting access of large external particles to the bacterial surface.

To test this possibility, susceptibility to bacteriophage infection was examined using three lytic *E. coli* phages differing in particle size and receptor specificity: P1 vir, λ vir and T7. Among them, P1 vir represented the largest particle (250–300 nm total length, 65–80 nm capsid diameter) [54], λ vir displayed intermediate dimensions (150–170 nm total length, head diameter 55–60 nm, flexible tail 135–150 nm) [55], whereas T7 was the smallest (60–70 nm diameter) [56]. In addition, λ vir recognizes the LamB maltose porin [57], while P1 vir and T7 utilize lipopolysaccharide as surface receptors [56,58].

**Fig 11:**
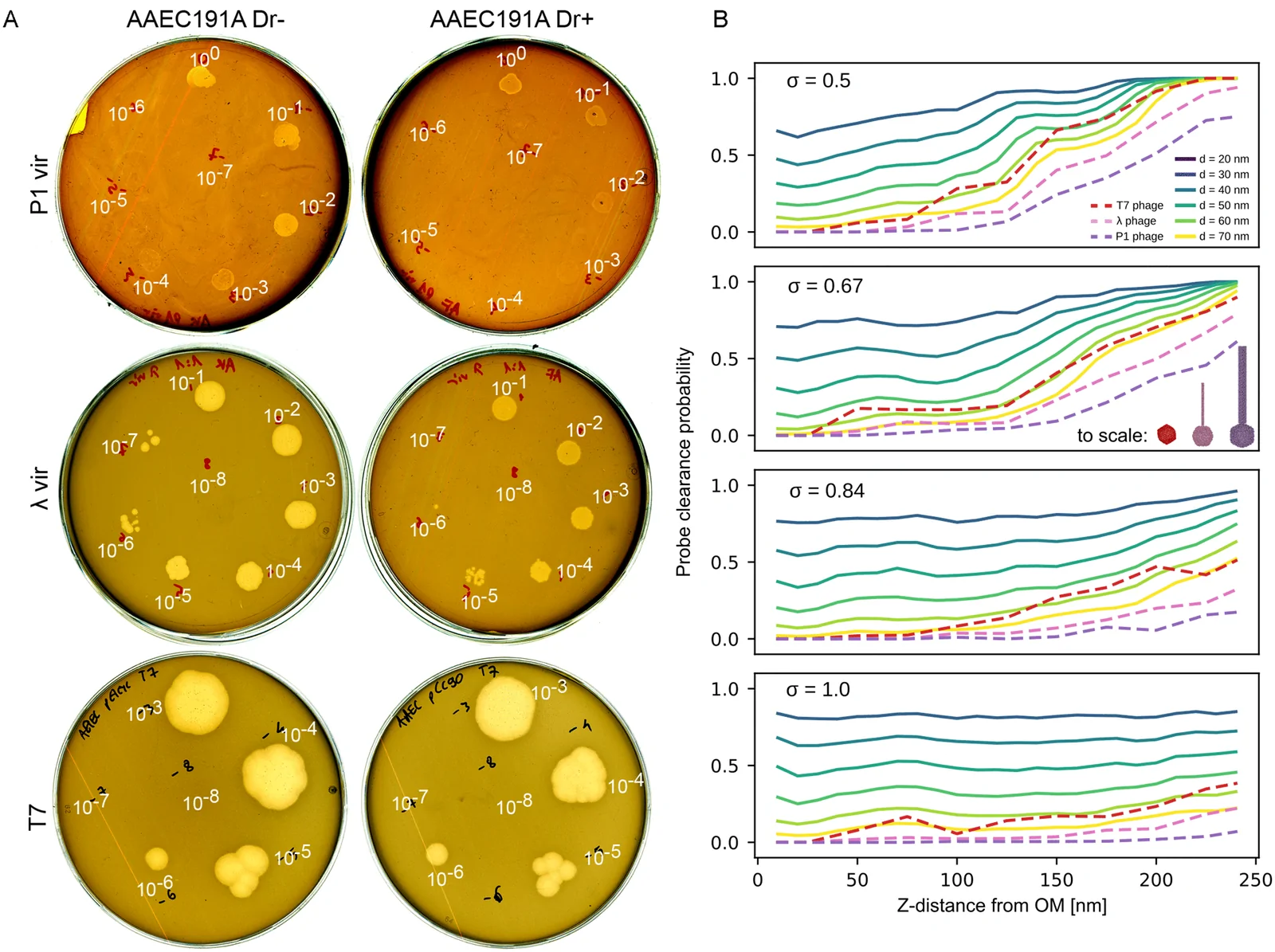
Dr fimbrial envelope limits bacteriophage access to the bacterial surface. (A) Representative plaque assays showing lysis induced by P1 vir, λ vir and T7 on lawns of *E. coli* AAEC191A Dr^+^ and Dr^−^ bacteria. Ten-fold serial dilutions of bacteriophage lysates (10^0^–10^−8^) were spotted onto LB top agar containing bacterial cells. Images show contrast-enhanced versions highlighting plaque boundaries. (B) Permeability of the fimbrial layer to model large-scale assemblies. The plots show probe clearance probability, defined as the probability to avoid clashes with the fimbrial mesh, as a function of the Z-distance from the outer membrane (OM). Probabilities were calculated for spherical probes of different diameter, as well as for 3 models of phages (T7, λ, P1), and were averaged over different probe placement and probe rotation (for non-spherical probes). Plots are shown for 4 different mesh geometries, corresponding to different values of the sigma parameter.

Efficiency of plating assays performed on AAEC191A and JM101 revealed a strong protective effect of the Dr envelope against infection by P1 vir and λ vir. The strongest reduction was observed for P1 vir, where the efficiency of plating (EOP) ratio calculated for Dr-positive relative to Dr-negative cells reached 0.02 ± 0.01 in JM101 and 0.116 ± 0.05 in AAEC191A. For λ vir, the corresponding values were 0.23 ± 0.05 and 0.10 ± 0.008, respectively. In contrast, T7 infection remained unaffected, with EOP ratios close to unity (1.1 ± 0.24 for JM101 and 1.04 ± 0.05 for AAEC191A), indicating that only larger phage particles were efficiently excluded by the Dr envelope (Figs 11A and S14, S2 Table in S1 File).

To determine whether reduced plaque formation resulted from impaired adsorption, phage binding assays were performed for the AAEC191A strain pair. The most pronounced difference was observed for P1 vir: 79.5 ± 9.2% of phage particles remained unbound in the Dr-positive strain, compared with only 2.2 ± 0.3% in the Dr-negative variant. For λ vir, the corresponding fractions of unadsorbed particles were 69 ± 8.5% and 26 ± 1.4%, respectively. In contrast, adsorption of T7 remained nearly identical in both variants, with approximately 30% of particles remaining unbound (S3 Table in S1 File). These results demonstrate that the Dr envelope strongly limits adsorption of larger phages, whereas access of T7 to the cell surface remains unaffected. Equivalent adsorption measurements were attempted for JM101 strains; however, the obtained values showed very high variability and were therefore not considered reliable for quantitative interpretation.

Because λ vir utilizes LamB as its receptor, LC–MS analysis of overnight culture pellets of AAEC191A Dr^+^ and AAEC191A Dr^−^  was performed to verify whether Dr fimbriae production alters LamB abundance (S15 Fig and S6 Table in S1 File). No differences in maltose outer membrane channel protein level were detected, indicating that reduced susceptibility to λ vir results from steric shielding rather than receptor downregulation.

As an additional control, M13 bacteriophage infection was examined using *E. coli* JM101, which constitutively produces F pili and is therefore naturally susceptible to M13 infection. Because M13 enters bacterial cells through F pili rather than outer membrane receptors [59], its infection pathway should remain independent of envelope-mediated shielding. Consistently, both Dr-positive and Dr-negative variants of JM101 exhibited identical susceptibility, with an EOP ratio of 1.03 ± 0.06 (S14 Fig and S2 Table in S1 File).

The structural model of the Dr envelope predicts that steric clashes with the fimbrial mesh will yield the observed sieve-like behavior for viral-size particles (larger than 20–30 nm) but remain irrelevant for low-molecular-weight compounds (Fig 11B). To verify this, susceptibility of AAEC191A Dr-positive and Dr-negative strains to five antibiotics was tested using minimum inhibitory concentration (MIC) and disc diffusion assays. Neither MIC values determined in LB broth nor inhibition zone diameters on solid medium differed significantly between both variants, indicating unrestricted penetration of small antimicrobial molecules through the Dr envelope (S16 Fig).

Together, these observations demonstrate that the Dr envelope functions as a size-selective molecular filter, efficiently restricting access of large biological particles while remaining permeable to small diffusible compounds.

### Validation of Dr capsule-dependent surface properties in the clinical UPEC strain IH11128

Because AAEC191A represents a simplified model for investigating Dr capsule-dependent surface properties, we next asked whether the principal phenotypes identified in this model could also be detected in the clinical UPEC strain IH11128. For this purpose, IH11128 was compared with its isogenic Dr-deficient transposon mutant DR14.

#### Physicochemical properties of the Dr capsule are retained in the clinical strain.

**Fig 12:**
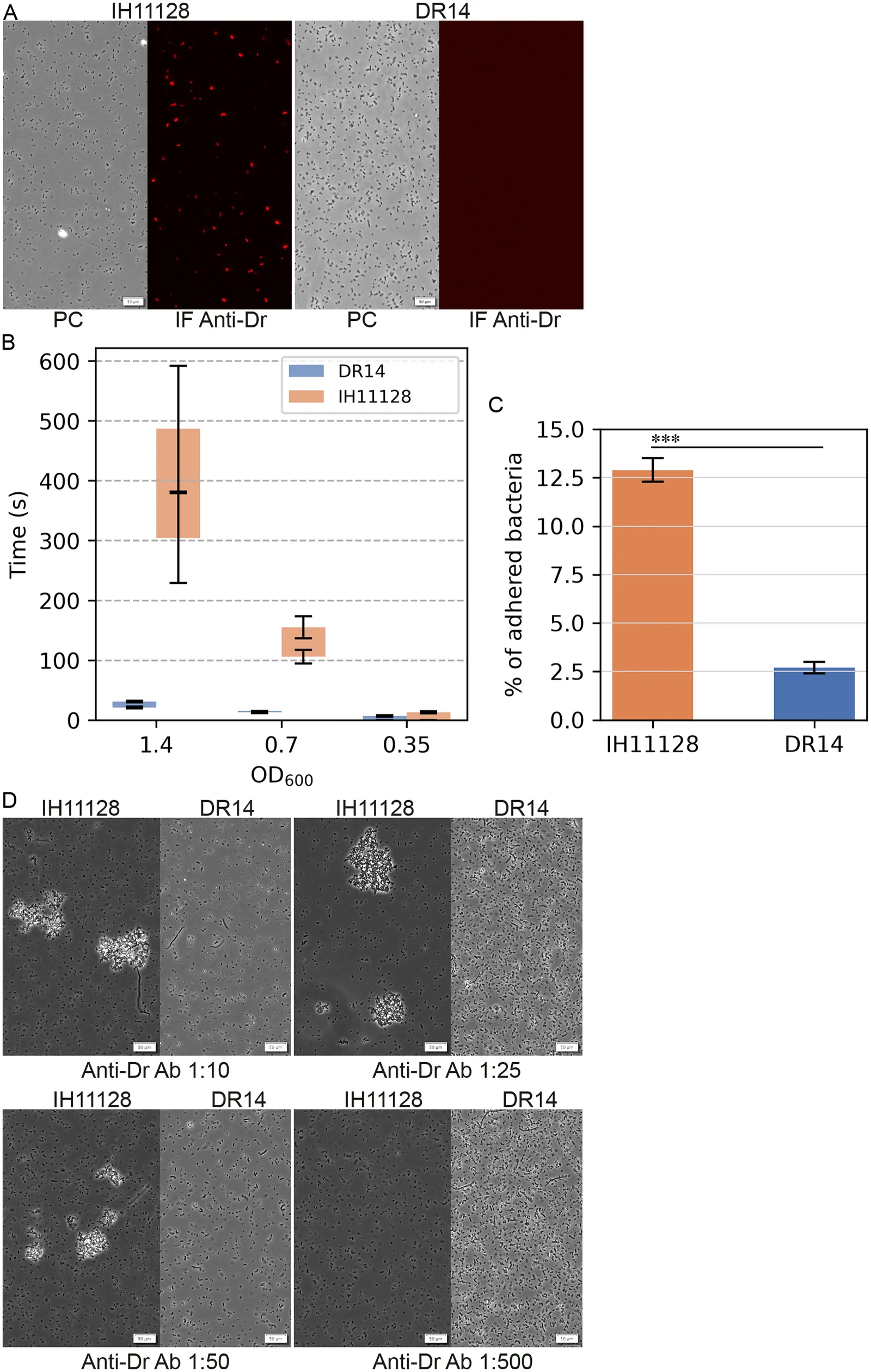
Validation of Dr-dependent surface properties in the clinical UPEC strain IH11128. (A) Representative phase-contrast (PC) and fluorescence (IF Anti-Dr) images of IH11128 and the isogenic Dr-deficient mutant DR14 following immunofluorescent labeling with rabbit polyclonal anti-Dr antibodies and TRITC-conjugated secondary antibodies. Fluorescence identifies the Dr-positive subpopulation present in the phase-variable IH11128 culture, whereas no specific labeling is observed for DR14. Scale bars, 20 µm. (B) Filtration time of IH11128 and DR14 suspensions through a 0.45 µm cellulose filter in 100 mM potassium nitrate as a function of OD_600_ (0.35, 0.70, and 1.40). The lower point indicates the appearance of the first filter area free of suspension, whereas the upper point indicates complete disappearance of liquid from the filter surface. Data represent mean ± SD (*n* = 3). Statistical significance was evaluated using the non-parametric Kruskal–Wallis test, showing a significant effect for IH11128 filtration (*p* < 0.001), whereas no significant differences were observed for DR14 filtration (*p* > 0.05). (C) Microbial adhesion to hydrocarbons (MATH) assay performed with IH11128 and DR14 suspensions using xylene as the organic phase. The percentage of bacteria transferred from the aqueous phase to the organic phase was calculated relative to OD_570_ values of control suspensions without organic solvent. Bacterial suspensions were adjusted to OD_600_ = 1.3 in PBS (pH 7.0). Data represent mean ± SD (*n* = 3), statistical significance was evaluated using the non-parametric Kruskal–Wallis test (*** *p* < 0.001). (D) Phase-contrast images showing antibody-mediated immunoagglutination of IH11128 and DR14 following incubation with rabbit polyclonal anti-Dr antibodies at the indicated dilutions (1:10, 1:25, 1:50, and 1:500). Large bacterial aggregates were observed only for IH11128, whereas DR14 remained uniformly dispersed at all antibody dilutions. Scale bars, 20 µm.

Immunofluorescence analysis confirmed the heterogeneous expression of Dr fimbriae in IH11128. Approximately 20% of bacterial cells displayed surface-associated Dr fluorescence, whereas no fluorescent cells were detected in the DR14 mutant (Fig 12A).

Despite the relatively small Dr-positive subpopulation, IH11128 exhibited pronounced physicochemical differences from DR14. During vacuum filtration, IH11128 suspensions passed through cellulose membranes markedly more slowly than DR14 over the entire range of optical densities tested. At OD_600_ = 1.4, complete filtration required 487 ± 105 s for IH11128 compared with 31 ± 1 s for DR14, while at OD_600_ = 0.7 the corresponding values were 155 ± 18 s and 15 ± 0 s, respectively (Fig 12B). The MATH assay likewise demonstrated altered surface properties. IH11128 showed significantly greater partitioning into xylene (12.9 ± 0.6%) than DR14 (2.7 ± 0.3%), whereas neither strain partitioned detectably into hexadecane (Fig 12C). Although the increase was smaller than that observed for the constitutively expressing AAEC191A Dr^+^ model, it demonstrated that phase-variable production of Dr fimbriae measurably modifies the surface properties of the clinical strain.

Unlike the constitutively Dr-positive AAEC191A strain, glutaraldehyde treatment did not induce detectable aggregation of IH11128, most likely because only a minority of cells expressed Dr fimbriae. However, immunoagglutination with polyclonal anti-Dr antibodies readily produced large bacterial aggregates at serum dilutions of 1:10, 1:25, and 1:50, whereas no aggregation was observed for DR14 or with antibody diluted 1:500 (Fig 12D).

#### Dr-producing cells exhibit enhanced mechanical stability of adhesion.

**Fig 13:**
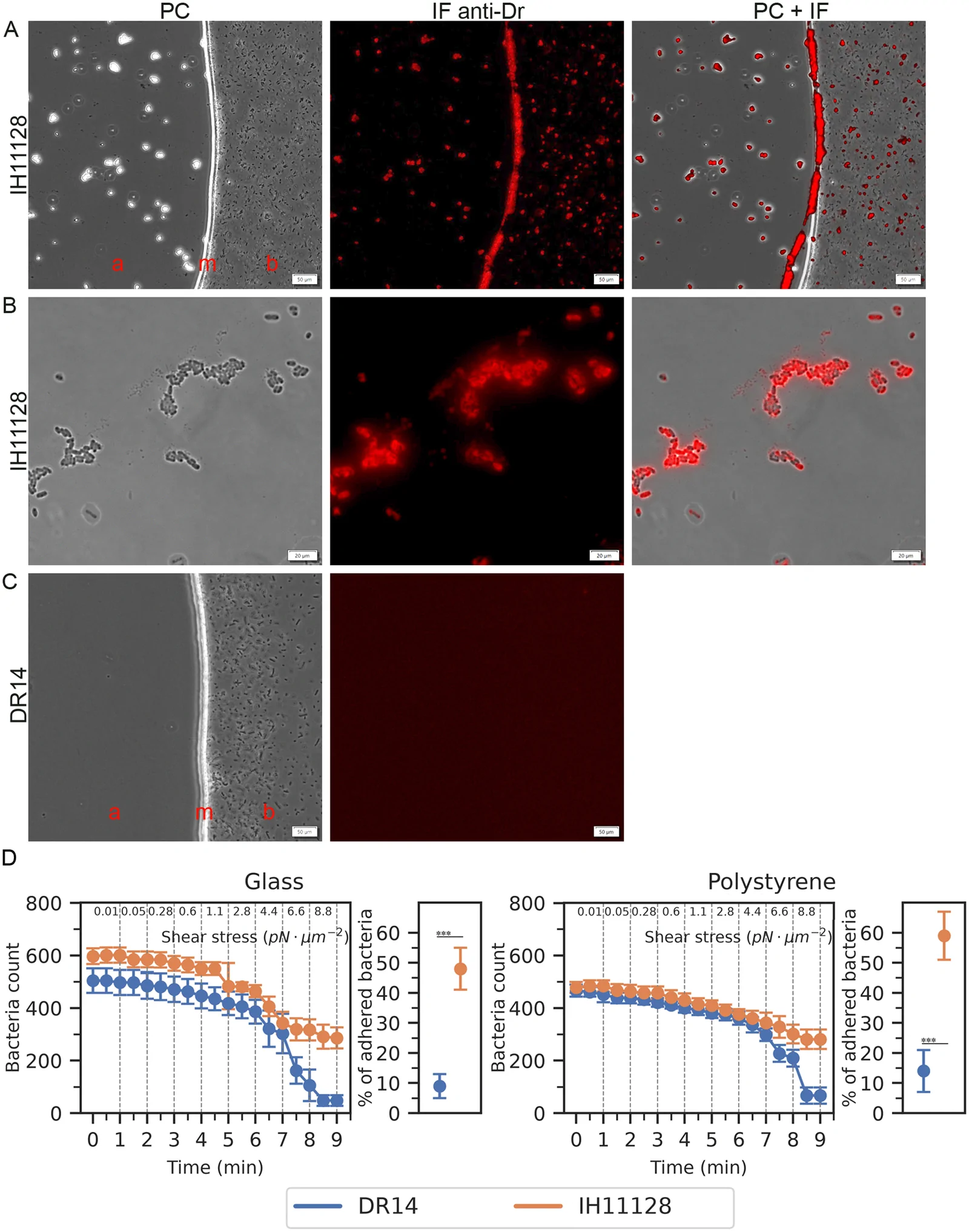
Validation of Dr-dependent adhesion under urinary conditions in the clinical UPEC strain IH11128. (A) Representative phase-contrast (PC), immunofluorescence (IF anti-Dr), and merged (PC + IF) images of the phase-variable clinical strain IH11128 during the meniscus retraction assay performed on glass in AU. The phase-contrast image shows the region exposed after meniscus withdrawal (a), the receding meniscus (m), and the bacterial suspension remaining ahead of the meniscus (b). Immunofluorescence identifies the Dr-positive subpopulation labeled with rabbit polyclonal anti-Dr antibodies and TRITC-conjugated secondary antibodies, while the merged image shows the spatial relationship between Dr-producing bacteria and the meniscus. Scale bars, 20 µm. (B) Representative phase-contrast, immunofluorescence, and merged images of IH11128 bacteria remaining attached to the glass surface after complete meniscus withdrawal. Immunofluorescence demonstrates that the retained bacteria belong almost exclusively to the Dr-positive subpopulation. Scale bars, 20 µm. (C) Representative phase-contrast and immunofluorescence images of the Dr-deficient mutant DR14 after meniscus withdrawal under identical conditions. No bacteria remained attached to the glass surface after passage of the meniscus, and no specific fluorescence signal was detected. Scale bars, 20 µm. (D) IH11128 and DR14 suspensions in AU (OD_600_ = 0.35) were introduced into flow chambers assembled on glass or untreated polystyrene surfaces and incubated for 10 min under static conditions to allow initial adhesion. Flow was then applied in sequential 1-min intervals generating increasing shear stresses of 0.01, 0.05, 0.28, 0.6, 1.1, 2.8, 4.4, 6.6, and 8.8 pN µm^−2^. The graphs show the number of adherent bacteria during stepwise increase of shear stress. The right panels indicate the percentage of initially attached bacteria remaining on the surface at the end of the flow experiment. Data represent mean ± SD. Statistical significance was evaluated using the non-parametric Kruskal–Wallis test (*** *p* < 0.001).

To determine whether the adhesion phenotypes observed in the AAEC191A model were retained in the clinical isolate, adhesion experiments were performed in AU. Following withdrawal of a receding liquid meniscus, only scattered IH11128 cells remained attached to the glass surface. Immunofluorescence demonstrated that virtually all retained bacteria belonged to the Dr-positive subpopulation, whereas Dr-expressing cells represented only a minor fraction of bacteria present before meniscus withdrawal (Fig 13A,B). In contrast, DR14 cells were removed almost completely from the surface and no attached bacteria remained after meniscus passage (Fig 13C). Laminar-flow experiments produced similar results. On polystyrene, IH11128 and DR14 displayed comparable initial adhesion (478 ± 21 and 466 ± 23 cells per field, respectively) and similar resistance to detachment up to 2.8 pN μm^−2^. At higher shear stresses, IH11128 remained significantly more resistant, with 59 ± 8% of initially attached bacteria remaining at 8.8 pN μm^−2^, compared with only 14 ± 7% for DR14 (Fig 13D). A comparable trend was observed on glass. Initial attachment reached 597 ± 30 bacteria per field for IH11128 and 504 ± 47 for DR14. Differences became evident above 4.4 pN μm^−2^, and at 8.8 pN μm^−2^ 48 ± 7% of IH11128 bacteria remained attached compared with only 9 ± 4% of DR14 cells (Fig 13D).

Together, these observations demonstrate that the mechanically stable adhesion phenotype identified in the AAEC191A model is also present in the clinical UPEC strain and is associated specifically with the Dr-producing bacterial subpopulation.

#### Dr fimbriae mediate selective association of clinical UPEC with calcium oxalate.

**Fig 14:**
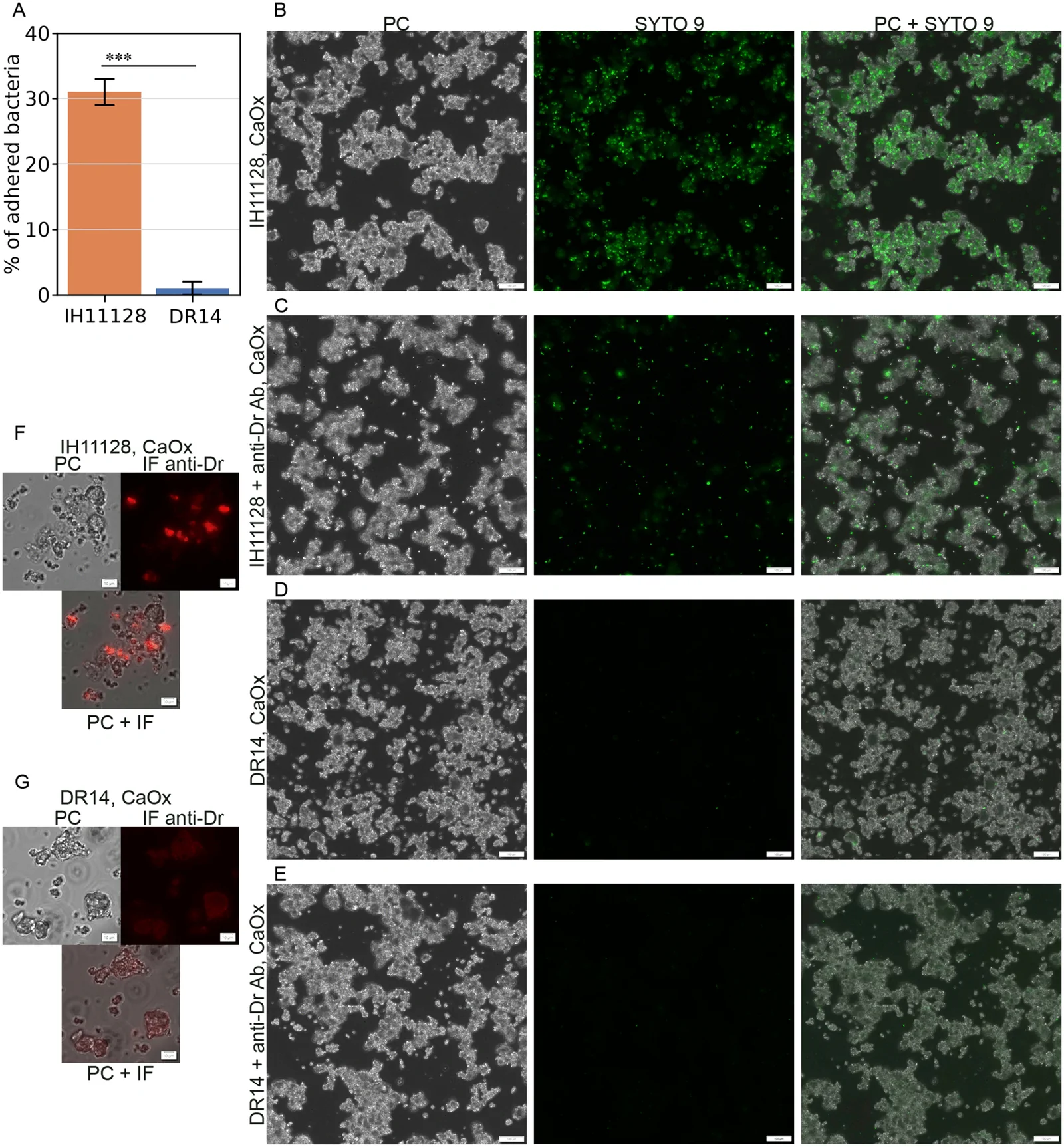
Dr fimbriae mediate specific binding of the clinical UPEC strain IH11128 to calcium oxalate crystals. (A) Percentage of IH11128 and DR14 bacteria transferred from suspension to CaOx during incubation in AU (pH 5.7) under agitation, calculated from the decrease in OD_600_ relative to control suspensions without crystals. Overnight cultures were resuspended in AU to OD_600_ = 0.8 and incubated with 1.7% (w/v) CaOx crystals for 40 min with gentle inversion followed by 30 min sedimentation. Data represent mean ± SD. Statistical significance was evaluated using the non-parametric Kruskal–Wallis test (*** *p* < 0.001). (B, D) Representative phase-contrast (PC), SYTO 9 fluorescence, and merged (PC+SYTO 9) images of IH11128 (B) and the Dr-deficient mutant DR14 (D) following incubation with CaOx crystals in AU. Phase-contrast images show crystal morphology, fluorescence images show SYTO 9-labeled bacteria, and merged images present the spatial relationship between bacteria and crystal surfaces. (C, E) Representative images of IH11128 (C) and DR14 (E) following pre-incubation with rabbit polyclonal anti-Dr antibodies (1:10 dilution) before incubation with CaOx. Imaging was performed as described for panels B and D. Scale bars in panels B–E, 100 µm. (F, G) Representative phase-contrast (PC), anti-Dr immunofluorescence (TRITC), and merged (PC+IF) images of IH11128 (F) and DR14 (G) incubated with CaOx crystals. Following incubation, samples were immunolabeled with rabbit polyclonal anti-Dr antibodies and TRITC-conjugated goat anti-rabbit secondary antibodies. TRITC fluorescence was detected exclusively on IH11128 bacteria associated with CaOx crystals, whereas no specific fluorescence signal was observed for DR14. Scale bars, 10 µm.

The interaction of IH11128 with CaOx was examined under the same AU conditions. Approximately 31 ± 2% of IH11128 bacteria became associated with CaOx crystals, whereas only 1 ± 1% of DR14 cells were removed from suspension (Fig 14A). Phase-contrast and SYTO 9 fluorescence microscopy confirmed extensive accumulation of IH11128 cells on crystal surfaces, while only occasional bacteria remained free in suspension (Fig 14B). Preincubation of IH11128 with polyclonal anti-Dr serum (1:10) reduced crystal-associated bacteria by approximately 90%, indicating that binding depends directly on Dr fimbriae (Fig 14C). In contrast, DR14 showed only sporadic interaction with CaOx, and anti-Dr antibodies produced no detectable effect (Fig 14D,E).

Immunofluorescence analysis further demonstrated that virtually all bacteria attached to CaOx crystals expressed Dr fimbriae (Fig 14F), whereas no fluorescence was detected for DR14 (Fig 14G). Considering that only approximately 20% of IH11128 cells expressed Dr fimbriae under these growth conditions, these observations indicate highly efficient and selective recruitment of the Dr-positive subpopulation to CaOx crystals.

#### Validation of the Dr molecular sieve model in the clinical background.

The bacteriophage experiments defined the scope of validation of the AAEC191A model. In the laboratory strain, the P1, λ, and T7 bacteriophage panel enabled calibration of the Dr envelope as a size-selective molecular sieve limiting access of bacteriophages to the bacterial surface. When the same bacteriophage panel was applied to IH11128 and DR14, however, no detectable lysis was observed for either strain (S17 Fig). This result is consistent with previous reports showing that the K capsule prevents infection by some phages through masking of their surface receptors [60,61]. Consequently, direct verification of the molecular sieve function of the Dr envelope was not possible in the IH11128/DR14 background. Validation of this aspect of the model will require bacteriophages capable of efficiently infecting the Dr-deficient strain DR14.

Collectively, these results demonstrate that the principal physicochemical, adhesive, and mineral-binding phenotypes identified using the AAEC191A model are reproduced in the clinical UPEC strain IH11128 despite phase-variable expression of the native *dra* operon, supporting the relevance of the model for investigating Dr capsule-dependent surface properties.

## Discussion

Our findings demonstrate that the Dr capsule represents a distinct supramolecular surface architecture that profoundly modifies the physicochemical properties of UPEC. The mechanistic framework proposed here was established using the AAEC191A model, which provides a simplified cell surface architecture lacking type 1 fimbriae, the K capsule, O-antigen-associated surface complexity, and other UPEC-specific surface structures that could mask the intrinsic properties of the Dr capsule. Because plasmid pCC90 lacks the regulatory region responsible for phase variation, AAEC191A Dr^+^ forms a homogeneous fimbria-positive population, allowing the physicochemical properties of the Dr capsule to be examined without interference from non-fimbriated cells. Under the experimental conditions used here, AAEC191A Dr^+^ produced Dr fimbriae at a level comparable to that of the Dr-positive subpopulation of the clinical strain IH11128. Validation experiments performed with IH11128 and its isogenic Dr-deficient mutant DR14 demonstrated that the principal physicochemical and functional properties identified in the AAEC191A model remained evident in the considerably more complex surface architecture of a clinical UPEC isolate. Together, these findings establish AAEC191A Dr^+^ as an appropriate experimental model for defining the intrinsic properties of the Dr capsule while confirming their biological relevance in a clinically relevant strain.

The structural analyses provide a coherent explanation for how individual Dr fimbriae become integrated into a continuous supramolecular surface layer. Transmission electron microscopy revealed a 200–300 nm electron-dense peripheral layer surrounding Dr-positive cells, while partial disruption of this structure exposed individual fimbrial bundles, demonstrating that the capsule is composed of densely packed fimbrial polymers rather than an amorphous extracellular matrix (S4 Fig) [62]. Quantitative proteomics further showed that stationary-phase AAEC191A Dr^+^ cells assemble approximately 2.2 × 10^5^ DraE subunits into surface polymers using only ~1.6 × 10^3^ DraC usher molecules. This stoichiometry implies the production of several thousand fimbrial polymers per bacterium, providing a structural basis for the formation of a continuous fimbrial capsule rather than isolated adhesive organelles. Computational modeling further indicates that the same number of Dr polymers may adopt different packing states depending on intermolecular repulsion and hydration, producing either a denser arrangement close to the bacterial surface or a more expanded hydrated shell while preserving the overall dimensions observed by TEM (Fig 2). Together, these observations indicate that the Dr capsule behaves as a dynamic hydrated fibrous network whose local organization can adapt to environmental conditions without losing its structural continuity.

The functional consequences of this organization were evident at the level of whole bacterial populations. Increased culture viscosity, loose sediment architecture, prolonged filtration times, and efficient glutaraldehyde-mediated crosslinking all indicate that neighboring bacteria interact through extended hydrated fimbrial interfaces rather than by direct contact between cell bodies (Figs 1, 4 and S13). Collectively, these findings support a model in which the Dr capsule becomes the dominant physicochemical interface of the bacterial surface, governing interactions with the surrounding environment. In clinical UPEC strains, however, this supramolecular organization is expected to operate within a considerably more complex surface architecture that includes additional structures such as the K capsule, type 1 fimbriae, and a complete O antigen. Accordingly, these components are likely to modulate the quantitative manifestation of Dr capsule-dependent phenotypes without altering the fundamental structural and physicochemical principles revealed by the AAEC191A model.

The proposed capsule architecture is unlikely to be unique to Dr-positive *E. coli*. Earlier ultrastructural studies have described remarkably similar surface layers in strains producing other members of the Dr adhesin family, including Afa-I, Afa-II, Afa-III, Afa-V, Dr-II, F1845, and Nfa [63–68]. A comparable supramolecular organization may also occur in members of the FGL subgroup of chaperone–usher adhesins [26], suggesting that the formation of hydrated fimbrial capsules could represent a more general structural principle among selected CU adhesin systems.

The physicochemical behavior of the Dr capsule indicates that this structure is amphipathic rather than conventionally hydrophilic or hydrophobic. Structural analysis of solvent-exposed DraE surfaces revealed a nearly balanced distribution of polar and apolar atoms, providing a molecular explanation for the seemingly paradoxical properties observed experimentally. Model Dr-positive bacteria displayed increased hydrocarbon affinity in the MATH assay while simultaneously exhibiting strong interactions with hydrophilic abiotic surfaces (Figs 3 and 5–9). In aqueous suspension, where fimbrial polymers remain fully hydrated and spatially extended, hydrophobic domains remain sufficiently exposed to promote partitioning into xylene. By contrast, contact-angle measurements performed on partially dehydrated bacterial layers detected only minor differences between Dr-positive and Dr-negative cells, indicating that collapse of the hydrated fimbrial architecture masks much of the physicochemical heterogeneity present in suspension. Together, these observations demonstrate that the physicochemical behavior of the Dr capsule results from the interplay between its amphipathic molecular composition and its highly hydrated supramolecular organization.

Electrokinetic measurements support the same conclusion. The zeta potential of model Dr-positive bacteria shifted consistently toward less negative values and closely approached that measured for isolated Dr fimbriae, indicating that the electrostatic properties of the bacterial surface become dominated by the fimbrial capsule (Fig 3A). Reduced long-range electrostatic repulsion is therefore likely to facilitate closer approach to abiotic surfaces, allowing short-range interactions mediated by exposed fimbrial residues to determine adhesion. Interestingly, previous studies have shown that other surface appendages, including P and type 1 fimbriae, also reduce the negative zeta potential of *E. coli* [43], suggesting that modulation of surface electrostatics may represent a more general consequence of fimbrial surface decoration.

Importantly, the clinical strain IH11128 confirmed the principal physicochemical predictions of the Dr capsule model despite the fact that only approximately 20% of the bacterial population expressed Dr fimbriae because of phase variation. Most strikingly, filtration times of IH11128 were comparable to those of the homogeneous AAEC191A Dr^+^ population, indicating that the presence of a relatively small Dr-positive subpopulation is sufficient to profoundly influence the rheological behavior of the entire bacterial suspension (Fig 12B). By contrast, the increase in hydrocarbon affinity measured by the MATH assay was substantially lower than in AAEC191A Dr^+^, closely reflecting the proportion of Dr-positive cells within the culture (Fig 12C). These observations suggest that different physicochemical properties of the Dr capsule are integrated at the population level in different ways, depending on the underlying mechanism. A similar conclusion emerges from the crosslinking experiments. Unlike the homogeneous AAEC191A Dr^+^ population, IH11128 did not form glutaraldehyde-induced aggregates, most likely because Dr-positive cells were predominantly surrounded by non-fimbriated bacteria, preventing efficient intermolecular crosslinking. However, specific immunoagglutination readily produced large bacterial aggregates, confirming that Dr-positive cells within the clinical population remained densely coated with Dr fimbriae (Fig 12D). Together, these findings indicate that the physicochemical properties of the Dr capsule are preserved in the clinical strain, whereas the magnitude of individual phenotypes depends on the proportion and spatial distribution of Dr-positive cells within the bacterial population.

This integrated physicochemical organization explains why Dr expression enhanced adhesion both to hydrophilic glass and hydrophobic polystyrene (Fig 5). However, the strength of these contacts differed markedly between substrates. On glass, model Dr-positive bacteria resisted capillary forces generated by a receding meniscus, with most cells remaining attached despite movement of the liquid front. Because capillary forces under these conditions reach the nanonewton range, resistance to detachment indicates stable multipoint anchoring of the hydrated envelope to the hydrophilic surface (Fig 6). This conclusion is further supported by the pronounced slowing of meniscus movement when the liquid front encountered attached Dr-positive cells, indicating strong pinning of the interface by surface-bound bacteria (S2 Video). The laminar-flow experiments support the same interpretation. Dr-positive bacteria accumulated efficiently on glass under low shear and retained adhesion under increasing flow, whereas attachment of Dr-negative cells remained weak and easily reversible (Fig 7). By contrast, on untreated polystyrene, although Dr expression increased initial attachment under static conditions, capillary forces removed essentially all cells.

Experiments performed in AU further confirmed these observations in the clinical UPEC background. Following withdrawal of the receding meniscus, virtually all bacteria remaining attached to the glass surface belonged to the Dr-positive subpopulation identified by immunofluorescence, whereas the Dr-deficient mutant DR14 was removed completely (Fig 13 A-C). Likewise, under laminar flow IH11128 and DR14 exhibited similar adhesion at low shear stresses, whereas clear differences emerged only under higher shear, where IH11128 consistently retained substantially more bacteria on both glass and polystyrene (Fig 13D). Thus, these findings indicate that increased resistance to detachment is a characteristic functional property of the Dr capsule that is preserved in both the simplified AAEC191A model and the clinical UPEC background.

The mineral interaction experiments further reveal that the Dr envelope modifies bacterial affinity toward clinically relevant urinary crystal phases. About 80% of human kidney stones are composed predominantly of CaOx and/or HA, making both phases directly relevant to urinary tract colonization [69,70]. The urinary pH values associated with these stones overlap closely with the experimental range examined here, particularly around pH 6.0–6.2, where the strongest Dr-dependent mineral interactions were observed [71,72].

AAEC191A Dr^+^ bacteria bound CaOx efficiently throughout the tested pH range of PBS, whereas HA binding occurred selectively at pH 5.7 and disappeared at neutral and alkaline pH (Figs 8 and 9). The strongest interaction of Dr-positive bacteria with both minerals was observed at pH 5.7, while at pH 7.0 and 8.0 binding progressively weakened. Non-fimbriated bacteria showed weaker and mechanically unstable attachment under identical conditions, indicating that the Dr envelope substantially increases mineral affinity under mildly acidic conditions.

The strong interaction with CaOx appears especially important because CaOx constitutes the dominant crystalline phase of most non-infectious kidney stones [73–75]. Increasing evidence indicates that calcium-based stones frequently contain viable bacteria, including *E. coli*, even when clinically classified as non-infectious. Microscopy, culture-based studies, and DNA analyses have revealed bacterial structures within non-struvite stone matrices [76–79]. Particularly important are recent observations showing bacterial imprints preserved within mineral architecture and demonstrating that bacterial biofilms are intrinsic components of calcium-based stones rather than superficial contaminants [80]. Experimental studies further indicate that bacterial cells and bacterial macromolecules actively promote CaOx nucleation, crystal growth, and aggregation [80–82]. In the stone nidus, regarded as the earliest stage of stone development, multiple organic lithogenic components have been identified, including proteins, lipids, erythrocyte membrane fragments, and bacterial material [83–85].

Our AU model further supports a potential role of the Dr envelope in kidney stone formation and stone-associated urinary tract infections. In AU adjusted to pH 5.7, AAEC191A Dr^+^ bacteria rapidly formed highly ordered pole-oriented associations with CaOx crystals within 2 h and generated large crystal-associated aggregates after 24 h that were absent in the Dr-negative control (Figs 10 and S10, S4 Video). In contrast, HA binding, which was very strong in PBS at the same pH, disappeared completely in AU (Figs 10 and S11, S5 Video). Dietary supplements containing potassium or magnesium citrate are widely used to prevent recurrent kidney stone formation by reducing calcium and oxalate supersaturation and directly inhibiting crystal growth [86–88]. Our results suggest an additional mechanism by which citrate may reduce bacterial interactions with urinary minerals. Citrate ions form soluble calcium complexes and progressively destabilize HA surfaces, thereby eliminating the exposed calcium-rich domains required for stable bacterial attachment (S12 Fig).

Importantly, the same CaOx-binding phenotype was independently confirmed in the clinical UPEC strain IH11128. Although only approximately 20% of the bacterial population expressed Dr fimbriae, nearly 30% of cells became associated with CaOx crystals under AU conditions, whereas the Dr-deficient mutant DR14 showed only background binding (Fig 14A). Immunofluorescence demonstrated that crystal-associated bacteria belonged almost exclusively to the Dr-positive subpopulation, and preincubation with anti-Dr antibodies reduced crystal binding by approximately 90% (Fig 14B,C). Together, these findings identify Dr fimbriae as the principal determinant of CaOx recognition in the clinical isolate.

The persistence of CaOx binding under urinary conditions may therefore have direct pathogenic implications. Studies in mouse models have shown that UPEC infection promotes CaOx deposition, whereas pre-existing CaOx deposits increase susceptibility to urinary infection [79]. Genome sequencing of UPEC isolates recovered from CaOx stones further revealed the presence of adhesins belonging to the Dr family [78], providing a clinical context for the mechanism proposed here. Our results suggest that Dr fimbriae may contribute to this process at two successive stages. First, DraE-mediated binding to decay-accelerating factor enables stable colonization of the renal epithelium under physiological shear conditions [25]. Subsequently, once CaOx crystals begin to form, the same fimbrial envelope provides an extended physicochemical interface that promotes bacterial capture by the mineral surface. The presented data indicate that the Dr envelope possesses several physicochemical properties that favor such interactions, including strong CaOx binding at urinary pH, a broad amphipathic hydrated surface, reduced negative zeta potential, and increased viscosity of bacterial suspensions. Conversely, established CaOx deposits may function as bacterial reservoirs that facilitate persistence and recurrence of urinary tract infection. Because Dr-positive UPEC are recognized etiological agents of pyelonephritis, this mechanism may contribute to long-term bacterial persistence within the upper urinary tract. Although the present findings provide experimental support for such a model, its contribution to human disease will require direct clinical validation.

The phage experiments reveal an additional fundamental property of the Dr envelope: it functions as a size-selective molecular sieve for biological particles. Autoaggregation experiments first showed that introduction of Dr fimbriae abolished sedimentation in naturally autoaggregative strains, indicating efficient masking of surface determinants responsible for cell-cell interactions (S13 Fig). This steric shielding was then directly demonstrated using three lytic bacteriophages differing in particle size: P1, λ and T7. These phages recognize different classes of *E. coli* receptors. P1 vir and T7 utilize lipopolysaccharide molecules distributed across the outer membrane, whereas λ vir recognizes the LamB porin [89,90]. The largest phage, P1, showed the strongest reduction in infection efficiency, λ exhibited intermediate inhibition, whereas the smallest phage, T7, infected Dr-positive and Dr-negative bacteria equally efficiently. Adsorption assays confirmed that reduced infectivity resulted from limited access to surface receptors rather than changes in receptor abundance. Particularly informative was the behavior of bacteriophage M13, whose infection remained completely unaffected by Dr expression because it enters through F pili rather than outer membrane receptors (S14 Fig). Together, these observations demonstrate that the Dr envelope selectively restricts access to outer membrane receptors while leaving receptor-independent entry pathways unaffected.

The experimental observations were fully consistent with the structural model of the Dr envelope. Computational analysis predicted that large particles such as bacteriophage P1 rarely penetrate below approximately 150 nm from the bacterial surface, whereas particles the size of bacteriophage T7 readily approach the outer membrane to within approximately 20–30 nm (Fig 11B). The identical susceptibility of Dr-positive and Dr-negative bacteria to T7 therefore provides independent validation of the predicted structural organization. At the same time, unchanged susceptibility to low-molecular-weight antibiotics demonstrates that the Dr envelope does not function as a diffusional barrier for small molecules (S16 Fig). Instead, it behaves as a molecular filter whose selectivity is determined primarily by particle dimensions.

Unlike the AAEC191A model, the clinical strain IH11128 was not susceptible to bacteriophages P1, λ or T7. This result is fully consistent with previous studies showing that the K capsule efficiently masks outer membrane receptors required for adsorption of some phages [61]. Consequently, the contribution of the Dr envelope cannot be analysed independently in this genetic background. Rather than conflicting with the proposed model, these observations emphasize the value of the simplified AAEC191A system, in which the absence of the K capsule and O-antigen-associated surface complexity allows the specific contribution of the Dr envelope to be resolved experimentally.

These observations may nevertheless have important implications for phage therapy. Many therapeutic bacteriophages recognize receptors located on lipopolysaccharide or outer membrane proteins and therefore must first overcome the extracellular structures surrounding the bacterial surface. In strains expressing a K capsule, this polysaccharide layer is likely to constitute the primary steric barrier. However, UPEC capsule production is dynamic, and numerous capsule-specific depolymerase-producing phages are capable of degrading capsular polysaccharides during infection [60]. Under these conditions, the Dr envelope may represent a second protective layer limiting access of larger virions to the outer membrane. Conversely, phages that utilize capsular polysaccharides as their primary receptors would bypass this first barrier but may still encounter steric restriction imposed by the underlying Dr envelope. The combined action of both extracellular structures may therefore influence phage adsorption efficiency in a phage-dependent manner and should be considered during the development of phage therapy against Dr-positive UPEC.

A similar role of a chaperone–usher fimbrial envelope in limiting bacteriophage infection has not been described previously. Interestingly, a recent study demonstrated that type 1 fimbriae protect *E. coli* against type VI secretion system-mediated killing, providing further evidence that extracellular appendages can function as physical barriers against biological antagonists [91]. However, the same principle may extend to other members of the Dr adhesin family and potentially to adhesins belonging to the FGL subgroup of chaperone–usher systems [26]. This concept is reminiscent of the F1 capsule of *Yersinia pestis*, where a dense hydrated polymer layer sterically limits contact between the bacterial surface and macrophage receptors, thereby preventing efficient phagocytosis [92,93]. Because bacteriophage P1 recognizes lipopolysaccharide as its receptor, the observed reduction in P1 susceptibility suggests that the Dr envelope likewise restricts physical access to LPS molecules. Whether such steric shielding also modulates recognition of Dr-positive bacteria by innate immune receptors remains to be determined. Nevertheless, these findings highlight the broader concept that supramolecular surface architectures can regulate bacterial interactions with large biological particles through purely physical mechanisms.

Taken together, the present work identifies the Dr capsule as a multifunctional supramolecular surface architecture whose biological role extends far beyond receptor-specific adhesion. By integrating structural, biophysical, physicochemical, and functional analyses with validation in a clinical UPEC isolate, this study demonstrates that the Dr capsule governs bacterial surface properties, enhances mechanical stability of adhesion under urinary conditions, mediates CaOx recognition, and functions as a size-selective molecular filter for biological particles. These findings establish the Dr capsule as a previously unrecognized determinant of the physicochemical interface between UPEC and its environment, providing a new framework for understanding how chaperone–usher adhesins contribute to bacterial persistence and pathogenesis within the urinary tract.

## Materials and methods

### Bacterial strains, culture media, and experimental buffers

The *E. coli* strain IH11128 (O75:K5:H-) is a clinical uropathogenic isolate recovered from a patient with pyelonephritis that expresses Dr fimbriae [94,95]. The isogenic *draC* insertion mutant DR14 was generated from IH11128 by insertional mutagenesis using a suicide vector, as previously described. Inactivation of *draC*, required for Dr fimbrial assembly, abolishes the surface expression of Dr fimbriae [96]. *E. coli* AAEC191A is a derivative of the K-12 laboratory strain carrying a deletion of the entire *fim* operon (Δ*fim*), generated by allelic exchange, which abolishes expression of type 1 fimbriae [97]. *E. coli* JM101 (ATCC 33876) and BL21(DE3) (Novagen) were used together with AAEC191A for heterologous production of Dr fimbriae encoded by plasmid pCC90. Plasmid pCC90, a derivative of pACYC177, carries the *dra* operon in which the promoter region and regulatory genes upstream of *draB* were deleted. The operon was cloned under control of the constitutive kanamycin resistance gene promoter [52]. The parental plasmid pACYC177 (New England Biolabs) was used to construct non-fimbriated control strains. Throughout the manuscript, strains carrying pCC90 and pACYC177 are referred to as Dr^+^ and Dr^−^, respectively.

Unless stated otherwise, all strains were maintained on LB broth with agar. Liquid cultures of the laboratory strains *E. coli* AAEC191A, BL21(DE3), and JM101 carrying either the Dr fimbriae-encoding plasmid pCC90 (Dr^+^) or the control plasmid pACYC177 (Dr^−^) were routinely grown in LB broth supplemented with ampicillin (100 μg/ml; Sigma-Aldrich). Liquid cultures of the clinical UPEC strain IH11128 were routinely grown in M63 minimal medium supplemented with 1% (w/v) casamino acids hydrolysate and 0.2% (w/v) glycerol, whereas the isogenic Dr-deficient mutant DR14 was grown under the same conditions with the addition of ampicillin (10 μg/ml). Unless indicated otherwise, pH-dependent experiments were performed with AAEC191A Dr^+^ and Dr^−^ bacteria suspended in PBS buffers of defined composition: pH 5.7 (130 mM NaCl, 0.1774 mM Na_2_HPO_4_, 19.82 mM NaH_2_PO_4_), pH 7.0 (130 mM NaCl, 10.36 mM Na_2_HPO_4_, 9.636 mM NaH_2_PO_4_), and pH 8.0 (130 mM NaCl, 18.3 mM Na_2_HPO_4_, 1.698 mM NaH_2_PO_4_). AU used in adhesion and crystal interaction assays contained NaCl (110 mM), Na_2_SO_4_·10H_2_O (10 mM), KH_2_PO_4_ (7 mM), K_2_HPO_4_ (7 mM), lactic acid (1.1 mM), citric acid (3 mM), NaHCO_3_ (2 mM), urea (170 mM), uric acid (0.4 mM), creatinine (7 mM), NH_4_Cl (25 mM), MgSO_4_·7H_2_O (2 mM), CaCl_2_·2H_2_O (1.663 mM), and FeSO_4_ (0.005 mM). The solution was adjusted to pH 5.7 and sterilized by filtration through a 0.22 µm membrane filter.

Bacterial concentration in cultures and suspensions was routinely estimated by OD_600_ measurements. For laboratory strains carrying either the Dr fimbriae-encoding plasmid pCC90 (Dr^+^) or the control plasmid pACYC177 (Dr^−^), Dr fimbriae production increased light scattering, resulting in higher OD_600_ values at identical cell numbers. Therefore, equivalent cell numbers were obtained by using a 1.3-fold higher OD_600_ for Dr^+^ than for Dr^−^ suspensions. This correction factor was established by direct cell counting in a Petroff chamber. In contrast, the clinical strain IH11128 contains only a subpopulation of Dr-expressing bacteria, resulting in no appreciable difference in OD_600_ relative to its isogenic Dr-deficient mutant DR14. Therefore, identical OD_600_ values were used for IH11128 and DR14 throughout the study.

Because BL21(DE3) and JM101 showed strong autoaggregation and sedimentation, they were excluded from experiments where spontaneous aggregation could affect interpretation of Dr fimbrial effects, including viscosity measurements, vacuum filtration, zeta potential determination, MATH, CA analysis, adhesion to abiotic surfaces, phage adsorption assays, and LC–MS analysis.

### Antisera and antibodies

Rabbit polyclonal anti-Dr antiserum (Immunolab, Gdynia, Poland) was generated by immunization with purified native Dr fimbriae isolated from *E. coli* AAEC191A carrying plasmid pCC90. The antibody titer, determined by ELISA against purified Dr fimbriae, was 1:11,500. This antiserum was used as the primary antibody in immunofluorescence, immunoaggregation, and Western blotting experiments. For Western blot analysis, horseradish peroxidase-conjugated goat anti-rabbit IgG secondary antibody (Goat Anti-Rabbit IgG, HRP-conjugate; Merck, Cat. #12–348) was used. For immunofluorescence, either rhodamine-conjugated goat anti-rabbit IgG (Goat Anti-Rabbit IgG, H & L Chain Specific Rhodamine Conjugate–TRITC; Calbiochem, Cat. #40 1321) or fluorescein-conjugated goat anti-rabbit IgG (Goat Anti-Rabbit IgG, H & L Chain Specific Fluorescein Conjugate–FITC; Calbiochem, Cat. #40 1314) was used as the secondary antibody.

### Isolation of Dr fimbriae from cell surfaces

An overnight culture of *E. coli* AAEC191A Dr^+^ adjusted to OD_600_ = 3.4 was used for fimbriae isolation. One milliliter of culture was collected, centrifuged, and the pellet resuspended in 500 µl PBS (pH 7.4). Samples were incubated at 65 °C for 3 h to release surface-associated fimbriae, followed by centrifugation at 1,500 × g for 5 min. Supernatants containing detached fimbrial fractions were collected and analyzed by LC–MS. For zeta potential measurements, fimbrial fractions were concentrated using Amicon Ultra-15 Ultracel-100K (Merck) and further purified by size exclusion chromatography on a Superdex 200 Increase 10/300 GL column (Cytiva) using the appropriate running buffer.

### Detection of Dr fimbriae with Western blotting

For all experiments involving Dr^+^ strains, the presence of Dr fimbriae was routinely verified by Western blotting. Bacterial suspensions were mixed with Laemmli buffer and either heated at 100 °C or incubated at room temperature for 60 min before separation by SDS–PAGE in 15% (w/v) polyacrylamide gels. Heating caused complete depolymerization of Dr fimbriae into DraE monomers, detected as a ~ 14 kDa band, whereas non-heated samples preserved the polymeric structure and remained at the top of the stacking gel because of their high molecular weight. Parallel analysis of heated and non-heated samples confirmed that DraE was present as polymerized fimbriae. Proteins were transferred to nitrocellulose membranes, which were blocked for 1 h at room temperature in PBS containing 1% (w/v) bovine serum albumin (BSA). Membranes were then incubated for 1 h at room temperature with rabbit polyclonal anti-Dr primary antibodies diluted 1:1,000 in PBS containing 1% BSA, followed by incubation for 1 h at room temperature with HRP-conjugated goat anti-rabbit IgG secondary antibodies (1:1,000 dilution in PBS containing 1% BSA).

### Relative quantification of Dr fimbriae in model AAEC191A Dr^+^ and IH11128 strains

To compare Dr fimbriae production between AAEC191A Dr^+^ and IH11128, both strains were cultured for 16 h at 37 °C with shaking in M63 medium supplemented with 1% casamino acids and 0.2% glycerol. Cells were harvested by centrifugation (5000 × g) and resuspended to OD_600_ = 2.5 before isolation of fimbrial fractions, which were analyzed by Western blotting. The relative DraE content in fimbrial fractions heated at 100 °C was determined densitometrically using a VersaDoc imaging system and Quantity One (Bio-Rad Laboratories). AAEC191A Dr^+^ was set as 100%, and DraE levels in IH11128 were calculated relative to this reference. To estimate the fraction of IH11128 cells actively producing Dr fimbriae, overnight cultures were harvested, resuspended in 1 ml PBS to an OD_600_ of 0.7, and analyzed by immunofluorescence microscopy. Bacterial suspensions were incubated for 1 h at room temperature with rabbit polyclonal anti-Dr primary antibodies (1:50 dilution in 1 ml PBS containing 1% BSA), followed by three washes with 1 ml PBS. Cells were subsequently incubated for 1 h at room temperature with FITC- or TRITC-conjugated goat anti-rabbit IgG secondary antibodies (1:100 dilution in 1 ml PBS containing 1% BSA), washed three times with 1 ml PBS, and examined by phase-contrast and fluorescence microscopy. Phase-contrast images were used to determine the total number of bacterial cells, whereas fluorescence images identified the Dr fimbriae-positive subpopulation. The percentage of Dr-positive cells was determined by counting bacteria in three randomly selected microscopic fields, each measuring 700 × 700 µm. The percentage of fimbriae-producing cells was used to correct DraE abundance values obtained by Western blotting. Three independent biological replicates, each including three technical replicates, were performed.

### Culture viscosity measurements

Two *E. coli* strains, AAEC191A Dr^−^ and AAEC191A Dr^+^, were used for viscosity measurements. Cells were grown in LB broth at 37 °C to OD_600_ ≈ 0.5 and diluted tenfold with fresh medium before introduction into the microfluidic system.

Viscosity and optical density were measured simultaneously using a droplet-based microfluidic method analyzing individual bacterial suspension droplets flowing through a microchannel [98]. The channel had a square cross-section of 600 × 600 µm and two optical sensors placed 1 m apart, allowing determination of droplet transit time and absorbance at 600 nm. A constant pressure difference of ~25 mbar maintained flow of the continuous phase, consisting of hexadecane with 1.0% Span-80 to minimize interfacial tension effects. The system was kept at 37 °C, and droplet volume was 2.16 µl. During measurements, droplets were repeatedly passed between sensors to obtain reproducible transit time and absorbance values. Viscosity was calculated from transit time using prior calibration with water–glycerol mixtures of known viscosity; shorter transit times indicated lower viscosity and longer times higher hydrodynamic resistance. Five independent biological replicates were performed.

### Vacuum filtration of bacterial suspensions

Overnight cultures of *E. coli* AAEC191A Dr^+^, AAEC191A Dr^−^, IH11128, and DR14 were harvested by centrifugation and washed once with PBS (pH 5.7, 7.0, or 8.0) or 0.1 M potassium nitrate. For experiments performed with the AAEC191A model, non-fimbriated bacteria were adjusted to OD_600_ = 0.65 and Dr-fimbriated bacteria to OD_600_ = 0.85 in PBS. In 0.1 M potassium nitrate, AAEC191A Dr^−^ cells were adjusted to OD_600_ = 0.32, 0.65, and 1.3, whereas AAEC191A Dr^+^ cells were adjusted to OD_600_ = 0.43, 0.85, and 1.7. Clinical strains IH11128 and DR14 were analysed only in 0.1 M potassium nitrate and were adjusted to OD_600_ = 0.35, 0.70, and 1.40. For each condition, 10 ml of suspension was filtered through 0.45 µm cellulose membrane filters (47 mm diameter) under constant vacuum at room temperature. Filtration time was recorded in two stages: (i) from the start of filtration to the appearance of the first central dry area on the filter surface, and (ii) until complete disappearance of liquid from the filter surface. Three independent biological replicates, each including three technical replicates, were performed. Results were statistically evaluated using the non-parametric Kruskal-Wallis test.

### Electron microscopy

The ultrastructure of bacterial cells was analyzed by transmission and scanning electron microscopy. For TEM, overnight cultures of *E. coli* AAEC191A Dr^+^ and Dr^−^ grown in LB broth were harvested by centrifugation and resuspended in PBS (pH 7.4) to OD_600_ = 0.1. A 5 µl aliquot of diluted suspension was applied to carbon-coated grids (GF Microsystems), negatively stained with 1.5% uranyl acetate (Sigma-Aldrich), and examined using a Tecnai G2 Spirit BioTWIN (FEI Company) operated at 120 kV. For SEM, morphology was examined using a Quanta FEG250 equipped with a secondary electron ET detector. Samples were coated with a 10 nm gold layer to prevent electrostatic charging, and images were acquired at 10 kV. For both TEM and SEM analyses, three independent biological replicates were performed, each comprising three technical replicates.

### LC–MS/MS analysis

Bacterial samples for proteomic LC–MS/MS analysis were prepared using a standardized workflow optimized for quantitative protein recovery. *E. coli* AAEC191A Dr^+^ and Dr^−^ were grown in LB broth at 37 °C with agitation to stationary phase (OD_600_ 3.3–3.5). Cell numbers were determined using a Petroff chamber (Marienfeld, Germany).

For total cell proteome analysis, 180–200 µl of culture was harvested by centrifugation and washed once with ice-cold 1 × PBS to remove medium residues. Metabolism was quenched by adding two volumes of pre-chilled methanol:ethanol (1:1, v/v; −20 °C), followed by brief vortexing and immediate freezing on dry ice. Samples were thawed on ice and dried using a SpeedVac (Eppendorf, Germany) at 30 °C for 4 h. Dried pellets were resuspended in 1 ml PBS, and protein concentration was measured at 280 nm. Samples were adjusted to 10–40 µg/µl. Fimbrial fractions were prepared as described above. For denaturation and reduction, 30 µl of protein solution (~20 µg/µl) was mixed with 30 µl water, 240 µl 8 M guanidine hydrochloride, 72 µl 300 mM Tris-HCl (pH 8.0) containing 12 mM CaCl_2_, and 12 µl 100 mM tris(2-carboxyethyl)phosphine. Samples were incubated at 37 °C for 30 min, then alkylated with 24 µl 300 mM iodoacetamide for 30 min in the dark at 37 °C. For enzymatic digestion, 34 µl of reduced and alkylated protein solution was mixed with 106 µl water, 10 µl 500 mM Tris-HCl (pH 8.0), and 10 µl 0.5 µg/µl Trypsin Platinum (Promega), then incubated overnight (16 h) at 37 °C. Digestion was stopped by adding 5 µl formic acid. Peptide samples were stored at −80 °C until analysis.

NanoLC–MS/MS analysis was performed using an ACQUITY M-Class (Waters) coupled to a ZenoTOF 7600 (SCIEX, Canada) with a nano-ESI source. Five microliters of peptide digest were injected onto a C18 trap column and separated on a reverse-phase C18 analytical column (75 µm × 100 mm, 2 µm particles). Mobile phase A was 0.1% formic acid in water, and mobile phase B was 0.1% formic acid in methanol. Flow rate was 350 nl/min. Mass spectrometric acquisition was performed in positive ion mode over m/z 400–3000 using full-scan acquisition with DIA (SWATH) and IDA MS/MS. Instrument settings were: ion source voltage 3800 V, collision energy 10 eV, curtain gas 35 psi, nebulizing gas 12 psi, source temperature 190 °C, and declustering potential 100 V. Raw data were processed with SCIEX OS and Spectronaut 18 (Biognosys). Quantitation was based on extracted ion chromatograms (XICs) with a ± 0.02 Da extraction window.

Absolute quantification of DraB, DraC, DraD, and DraE proteins was performed using synthetic peptide standards (Lipopharm, Gdańsk, Poland) corresponding to unique tryptic peptides: SPAPFVVTPPLFR (DraB), IEEYPALFR (DraC), TGGDGWSPVK (DraD), and FFLISDNNR (DraE) (S4 Table in S1 File). Peptides (>95% purity) were dissolved in LC-MS-grade water containing 5% methanol and added to digested biological samples to generate calibration curves (S5 Table in S1 File, S18 Fig). Doubly charged precursor ions ([M + 2H]^2+^) were quantified at m/z values 714.4059 (DraB), 569.3006 (DraC), 502.2458 (DraD), and 563.2881 (DraE). Extracted ion chromatograms were generated using a ± 0.01 Da mass window. Calibration curves were obtained from technical triplicates and used for absolute protein quantification. Retention times used for peptide identification were 27.92 min (DraB), 24.34 min (DraC), 19.34 min (DraD), and 24.27 min (DraE). Analytical sensitivity was estimated using the standard calibration-based equations for the limit of detection (LOD) and limit of quantification (LOQ): LOD = 3.3 × σ/S and LOQ = 10 × σ/S, where S denotes the slope of the calibration curve and σ denotes the standard deviation of the response estimated from the regression model. The amount-based LOD and LOQ values were subsequently converted to particles per cell using the quantitative normalization applied in the study. DraB showed the lowest calculated thresholds, with an LOD of 22.49 particles per cell and an LOQ of 68.12 particles per cell. DraD showed intermediate sensitivity (LOD 33.98, LOQ 103.03 particles per cell), whereas DraE and DraC exhibited the highest calculated thresholds, both with LOD values of approximately 63 particles per cell and LOQ values of approximately 191 particles per cell.

Three independent biological replicates, each including three technical replicates, were performed. Results were statistically evaluated using the unpaired two-tailed Student’s t-test.

### Structural modeling of the Dr fimbrial envelope

A structural model of the Dr fimbrial envelope produced by *E. coli* Dr^+^ was constructed using the known structure of self-complemented AfaE as a template and the established DraE donor strand complementation dimerization mode [20]. A linear 7-mer polymer composed of seven identical DraE chains was generated using MODELLER [99].

Polymer conformational sampling was performed in three independent 500-ns equilibrium simulations using GROMACS with the amber99SB-ILDN force field. Simulations were carried out in an elongated 10 × 10 × 36 nm simulation box containing 113071 TIP3P water molecules and 0.15 M KCl [100,101], representing a short fimbrial segment embedded in a dense fimbrial layer. From the combined trajectories, 450 conformational snapshots were extracted and divided into five overlapping dimer geometries each, excluding the terminal N-terminal dimer, resulting in a pool of 2250 DraE dimer conformations. These dimer geometries were subsequently used to assemble grid models of the fimbrial layer consisting of 5 × 5 units, corresponding to experimentally determined DraC usher surface density (approximately 1 per 3970 nm^2^) and the DraE/DraC ratio, approximated as 150 DraE subunits per fimbrial chain.

Assembly of the fimbrial layer was performed using a Monte Carlo procedure. An initial dimer was positioned at Z = 0 with random lateral displacement in the XY plane, and subsequent extension steps were generated by aligning randomly selected dimers from the simulation-derived pool onto the terminal DraE subunit using MDTraj [102]. Proposed extensions were rejected if they produced steric clashes, penetrated the membrane region (center of mass below 2 nm), or introduced downward-directed growth with excessive probability. Periodic boundary conditions in the XY plane were applied during clash detection. To reconstruct the full envelope geometry, the resulting 315 × 315 nm patch was transformed into a cylindrical segment with a radius of 500 nm, corresponding to the average radius of an *E. coli* cell. Nine successive 36° rotations around the Y axis were applied to generate a complete cylindrical surface, followed by axial replication using VMD [103]. Additional geometric manipulations were performed using Gromologist [104].

Protein density profiles and steric clash probabilities were calculated using custom Python scripts. For large-scale assembly, a mesoscale representation of the fimbrial layer was generated by reducing each 139-residue DraE unit to 10 representative Cα atoms selected by k-medoid clustering, yielding final cylinder segments containing 377500 particles, corresponding to approximately 78 million atoms. To obtain smoothed density profiles, cylinder segments were projected on the xy plane, offset to align the profile with the edge of the cylinder representing the bacterium, and normalized to a maximum of 1 at that point. The corresponding density profiles from TEM micrographs were calculated using ImageJ.

#### Phage clearance probability calculations.

To calculate clearance probabilities for the three analyzed bacteriophages (T7, λ vir and P1 vir) simplified structural models of phage particles were generated using available crystallographic data for capsid and tail components combined with literature-reported tail lengths. For T7, only the capsid geometry was considered, based on PDB entry 2XVR. For λ phage, structural elements from PDB entries 8K36 and 7VII were combined. For P1, entries 8JAJ and 6O3H were used, with the unknown head geometry approximated using the similarly sized capsid of phage P74-26, both reaching approximately 85 nm in diameter. To reduce computational cost, every fourth Cα atom from each phage model was retained for steric sampling.

At each defined Z-offset relative to the bacterial outer membrane, the center of mass of the phage head was translated to each of the 25 grid positions of the fimbrial layer. For every grid position, 24 random rotational orientations were sampled. Steric clashes between the phage model and the XY-periodic fimbrial layer were identified when any two atoms approached within 1 nm. Clearance probabilities were calculated as averages over 600 independent translation–rotation configurations (25 positions × 24 rotations) for each Z-coordinate.

### Microbial adhesion to hydrocarbons (MATH)

Overnight cultures of *E. coli* AAEC191A Dr^+^, AAEC191A Dr^−^, IH11128, and DR14 were suspended in PBS (pH 7.0). AAEC191A Dr^+^, IH11128, and DR14 were adjusted to OD_600_ = 1.3, whereas AAEC191A Dr^−^ was adjusted to OD_600_ = 1.0 to obtain comparable bacterial cell numbers. For each measurement, 3 ml of bacterial suspension was mixed with 1 ml of organic phase (xylene or hexadecane). Control samples without organic phase were prepared in parallel. Samples were vortexed for 90 s and incubated 15 min at room temperature for phase separation. One milliliter of the aqueous phase was collected, and absorbance at 570 nm was measured. The percentage of adhesion, reflecting surface hydrophobicity, was calculated as:


$$\begin{array}{c} \%\;of\;adhesion=\frac{A_{\text{0}}-A_{\text{1}}}{A_{\text{0}}}\times \text{100\%} \end{array}$$

where A₀ is the absorbance of the control sample, and A_1_ is that of the aqueous phase after mixing.

For microscopic observation, 5 µl of bacterial suspension prepared as described for the MATH assay were placed into a 5 mm × 2 mm well on a glass slide, which was then filled with the respective organic phase and sealed with a coverslip. After 15 min, the water–organic interface was observed using an inverted phase-contrast microscope.

Three independent biological replicates, each including three technical replicates, were performed. Results were statistically evaluated using the non-parametric Kruskal-Wallis test.

### Determination of wetting contact angle and ΔG_BWB_

Bacterial lawns of *E. coli* AAEC191A Dr^+^ and Dr^−^ were prepared on cellulose membrane filters as in the vacuum filtration section. Briefly, 10 ml of suspensions in 100 mM potassium nitrate were filtered, using OD_600_ = 0.85 for Dr^+^ and 0.65 for Dr^−^ cells. The confluent multilayers were washed with 5 ml deionized water, dried at room temperature for 50 min, and analyzed within 30 min. Complete coverage of the filters was confirmed by scanning electron microscopy.

Contact angles were measured using a Ramé-hart Model 90-U3-PRO system with DROPimage Pro. Deionized water, formamide, and diiodomethane (Sigma-Aldrich) served as probe liquids. Droplets were applied as freely falling drops from a height of 1 cm using a Gilmont GS-1200 Micrometer Syringe. Contact angles were determined with DROPimage Pro, with ten independent measurements per sample and liquid. Three independent biological replicates, each including three technical replicates, were performed.

Surface free energy parameters were calculated using the Good–van Oss–Chaudhury model [105]. The hydrophobicity of bacterial surfaces was expressed as the free energy of interaction between bacterial surfaces immersed in water according to the following equation:


$$\begin{array}{c} {\Delta G}_{BWB}=-\text{2}{\left(\sqrt{\gamma_{B}^{LW}}-\sqrt{\gamma_{W}^{LW}}\right)}^{\text{2}}+\text{4}\left(\sqrt{\gamma_{B}^{+}\gamma_{W}^{-}}+\sqrt{\gamma_{B}^{-}\gamma_{W}^{+}}-\sqrt{\gamma_{B}^{+}\gamma_{B}^{-}}-\sqrt{\gamma_{W}^{+}\gamma_{W}^{-}}\right) \end{array}$$

where: ∆𝐺_BWB_ – value of free energy of interaction between bacteria when immersed in water, B – bacteria, W – water, 𝛾^𝐿𝑊^, 𝛾^+^, 𝛾^−^ – Lifshitz-van der Waals (dispersive), acid (electron acceptor), and base (electron donor) components of surface free energy. The values of 𝛾^𝐿𝑊^, 𝛾^+^, 𝛾^−^ for water were taken from reference [106].

### Zeta potential measurements

Overnight cultures of *E. coli* AAEC191A Dr^+^ and Dr^−^ were harvested and suspended in PBS at pH 5.7, 7.0, and 8.0 to OD_600_ = 0.5 and 0.4, respectively. Measurements were performed using a Zetasizer Nano ZS (Malvern Instruments, UK) based on laser Doppler electrophoresis with a 633 nm red laser. Samples were loaded into DTS1060 folded capillary cells and equilibrated for 120 s at 25 °C. Zeta potentials were calculated using the Smoluchowski approximation. Temperature was maintained at 25 °C using the Peltier-controlled holder. Dispersant parameters for calculations were: viscosity 0.89 cP, refractive index 1.33, and dielectric constant 78.5. Instrument control and data analysis were done with Malvern Dispersion Technology Software. Three independent biological replicates, each including three technical replicates, were performed. Six independent measurements were conducted per sample, each with at least 15 sub-runs and 120 s intervals. Results were statistically evaluated using the Welch’s corrected Student’s t-test.

### Effect of glutaraldehyde and antibody-mediated cross-linking of bacterial cells

Overnight cultures of *E. coli* AAEC191A Dr^+^, AAEC191A Dr^−^, IH11128, and DR14 were harvested and resuspended in 1 ml PBS (pH 7.5) containing 0.05% Tween 20 to OD_600_ = 1.0. Glutaraldehyde (25% stock solution) was added to final concentrations of 2%, 1%, 0.5%, 0.25%, 0.1%, and 0.01%. Samples were incubated for 15 min at 4 °C to allow cross-linking. Reactions were quenched by adding 300 µl PBS containing 50 mM glycine and incubated for an additional 15 min at room temperature. The degree of cross-linking was evaluated by phase-contrast microscopy by determining the lowest glutaraldehyde concentration inducing visible bacterial aggregates and the maximum aggregate size observed at each concentration.

To assess Dr-specific immunoagglutination, IH11128 and DR14 cells were resuspended in 1 ml PBS (pH 7.5) supplemented with 0.1% BSA to OD_600_ = 1.0. Rabbit polyclonal anti-Dr antibodies were added at final dilutions of 1:10, 1:25, 1:50, and 1:500. Samples were incubated for 1 h at room temperature with gentle mixing, and bacterial aggregation was evaluated by phase-contrast microscopy using an inverted microscope.

Three independent biological replicates, each including three technical replicates, were performed.

### Assessment of bacterial adhesion to glass and polystyrene surfaces under static conditions

Overnight cultures of *E. coli* AAEC191A Dr^+^ and Dr^−^ were harvested and resuspended in PBS at pH 5.7, 7.0, or 8.0 to OD_600_ = 0.7. Aliquots of 40 µl were deposited onto glass or polystyrene surfaces and incubated for 15 min at room temperature under static conditions. Non-adherent cells were removed by gently rinsing with 1 ml of the corresponding buffer. Attached bacteria were visualized by phase-contrast microscopy and quantified using the ‘Count and Measure’ module in cellSens Dimension 4.1. For each slide, bacteria were counted in 10 fields, each covering an area of 100 µm^2^. Three independent biological replicates, each including three technical replicates, were performed. Results were statistically evaluated using the non-parametric Kruskal-Wallis test.

### Meniscus retraction assay on glass and polystyrene surfaces

Overnight cultures of *E. coli* AAEC191A Dr^+^ and Dr^−^ were harvested by centrifugation and resuspended in PBS (pH 7.0) to an OD_600_ of 0.5. Aliquots of 10 µl of bacterial suspension were deposited onto glass slides or untreated polystyrene surfaces and covered with coverslips. Samples were maintained at 25 °C and constant humidity. Meniscus movement was induced by gradual evaporation of water from the edge of the coverslip, while the bacterial liquid layer remained at 10 ± 2 µm. Meniscus motion was recorded using an inverted Olympus IX73 microscope equipped with a UPlanFL N 20 × /0.30 objective and a Hamamatsu Photonics ORCA-Flash2.8 camera, providing a field of view of 696.2 × 522.2 µm and a spatial resolution of 0.363 µm per pixel. Images were acquired at 5 frames s^−1^ for 60 s on glass and 80 s on polystyrene. Meniscus velocity was determined from the distance traveled during image acquisition. Video files were converted to MP4 using Kdenlive. Bacteria remaining after meniscus passage were quantified by counting cells in 10 fields, each covering an area of 100 µm^2^, before and after meniscus movement within the same regions using the ‘Count and Measure’ module in cellSens Dimension 4.1.

For experiments performed with the clinical strains, only glass surfaces were used. To identify the Dr-positive subpopulation, IH11128 and DR14 cells (1 ml PBS pH 7.4, OD_600_ = 1.0) were incubated for 1 h at room temperature with gentle mixing in the presence of rabbit polyclonal anti-Dr antibodies diluted 1:200. The high antibody dilution was selected to minimize interference with Dr-mediated adhesion while allowing reliable identification of Dr-producing cells by fluorescence microscopy. Cells were washed once with 1 ml PBS and subsequently incubated for 1 h with TRITC-conjugated goat anti-rabbit secondary antibodies diluted 1:200. After a single wash with AU, bacterial suspensions were resuspended in AU to OD_600_ = 0.5, and the meniscus retraction assay was performed on glass as described above. The use of AU reproduced the urinary conditions employed in the remaining validation experiments with the clinical strains. Following meniscus passage, bacteria remaining attached to the glass surface were examined by both phase-contrast and fluorescence microscopy, allowing discrimination between Dr-positive and Dr-negative cells within the IH11128 population.

Three independent biological replicates, each including three technical replicates, were performed.

### Adhesion to glass and polystyrene surfaces under laminar flow conditions

Adhesion of overnight cultures of *E. coli* AAEC191A Dr^+^ and Dr^−^ to glass and untreated polystyrene surfaces was analyzed under laminar flow-induced shear stress. Bacterial suspensions were prepared in PBS at pH 5.7, 7.0, or 8.0 to OD_600_ values of 0.4 for Dr^+^ and 0.3 for Dr^−^ cells. Suspensions were introduced into a parallel plate flow chamber (GlycoTech) with a silicone gasket forming a flow cell 2.5 mm wide, 10 mm long, and 0.25 mm high. The chamber was attached to the surface by vacuum, and flow was generated using a syringe pump (Harvard Apparatus).

***Resistance of surface-bound bacteria to detachment by shear stress.*** Bacterial suspensions in the respective buffers were introduced into the flow chamber and incubated without flow for 10 min to allow adhesion. Subsequently, sequential one-minute flow intervals generating increasing shear stresses of 0.01, 0.05, 0.28, 0.6, 1.1, 2.8, 4.4, and 6.6 pN µm^−2^ were applied to assess detachment of surface-bound bacteria.

For experiments performed with the clinical strains, IH11128 and DR14 were suspended in AU to OD_600_ = 0.35. Detachment assays were performed on both glass and untreated polystyrene surfaces following the same protocol as described above, except that an additional shear stress of 8.8 pN µm^−2^ was applied at the end of the detachment series.

***Accumulation of bacteria under shear stress.*** To investigate adhesion kinetics under flow, bacterial suspensions were passed through the chamber for 15 min at constant shear stresses of 0.01, 0.05, or 0.28 pN µm^−2^. This experiment was performed only for bacteria in PBS at pH 5.7, as adhesion under flow at pH 7.0 and 8.0 was negligible.

Time-lapse videos were recorded using the same imaging setup as in the experiment analyzing bacterial detachment by the moving meniscus. The acquisition rate was adjusted to match the flow velocity, ranging from 2 frames s^−1^ at a shear stress of 0.01 pN µm^−2^ to 50 frames s^−1^ at shear stress values above 6.6 pN µm^−2^. For both detachment and accumulation analyses, three independent biological replicates were performed, each comprising three technical replicates. Statistical analysis of bacterial attachment and accumulation was performed as described in reference [25].

### Adhesion of bacteria to calcium salt crystals

Overnight cultures of *E. coli* AAEC191A Dr^+^ and Dr^−^ were harvested and resuspended in PBS adjusted to pH 5.7, 7.0, or 8.0 to OD_600_ values of 0.9 and 0.7, respectively. To 5.5 ml of bacterial suspension, 500 µl of a 20% suspension of CaOx monohydrate or HA prepared in the corresponding buffer was added. Samples were incubated for 40 min with gentle inversion followed by 30 min sedimentation. Immediately after mixing, 500 µl aliquots were collected for further analyses. Control samples were prepared by replacing crystals with PBS. After incubation, the optical density of the supernatant was measured at 600 nm. The percentage of bacterial adhesion was calculated as described for the MATH assay, where *A₀* represents the absorbance of the control without crystals and *A_1_* the absorbance of the sample incubated with CaOx or HA. To evaluate bacterial adhesion under conditions resembling urine, the same assay was performed using AU instead of PBS.

For validation experiments, the clinical strains IH11128 and DR14 were examined only for interactions with CaOx. Overnight cultures were resuspended in AU to OD_600_ = 0.8, and the adhesion assay was performed as described above.

Three independent biological replicates, each including three technical replicates, were performed. Results were statistically evaluated using the non-parametric Kruskal–Wallis test.

#### Visualization of bacteria bound to calcium oxalate crystals.

For the AAEC191A model, bacteria bound to CaOx and HA crystals were visualized by staining with DAPI. Briefly, 5 µl of DAPI (2 mg ml^−1^) was added to bacterial–crystal suspensions, followed by incubation for 1 h at room temperature with gentle mixing. Samples were centrifuged at 9 × *g* for 5 min, supernatants were removed, and pellets were gently resuspended in 0.5 ml of the corresponding buffer. To evaluate binding strength, samples were subjected to three washing cycles with 1.5 ml of the corresponding buffer and vigorous pipetting, followed by centrifugation at 9 × *g* for 5 min after each wash. Pellets were finally resuspended in 0.3 ml buffer and analysed by phase-contrast and fluorescence microscopy.

For the clinical strains, bacteria associated with CaOx crystals were visualized using SYTO 9 (final concentration 1 µM). Stained samples were incubated for 1 h at room temperature with gentle mixing and subsequently washed three times with 1.5 ml AU using the same centrifugation conditions as described above. Pellets were finally resuspended in AU and examined by phase-contrast and fluorescence microscopy.

To determine whether CaOx binding depended specifically on Dr fimbriae, IH11128 suspensions were pre-incubated for 1 h at room temperature with rabbit polyclonal anti-Dr antibodies diluted 1:10 before addition of CaOx crystals. Crystal-binding assays were then performed as described above.

To identify Dr-positive bacteria associated with CaOx crystals, samples obtained from the standard IH11128 binding assay were subjected to immunofluorescence staining. Crystal-associated bacteria were incubated for 1 h at room temperature in PBS (pH 7.4) containing 0.1% BSA with rabbit polyclonal anti-Dr antibodies diluted 1:50, washed once with PBS, and subsequently incubated for 1 h with TRITC-conjugated goat anti-rabbit secondary antibodies diluted 1:100. Following a final PBS wash, samples were analysed by phase-contrast and TRITC fluorescence microscopy.

### Interaction of the model AAEC191A Dr^+^ and Dr^−^ strains with HA and CaOx crystals during growth in artificial urine

A sterile polystyrene 24-well plate was prepared by adding 1.5 ml of AU to each well. Overnight bacterial cultures were diluted in AU to OD_600_ = 0.025 and used to inoculate the wells. HA and CaOx were added to selected wells to a final concentration of 0.05% (w/v), while two control wells contained AU inoculated with Dr^+^ or Dr^−^ bacteria, without minerals. The plate was incubated at 37 °C under static conditions. After 2 h of incubation, all experimental variants were analyzed by phase-contrast microscopy using the same imaging setup as in the experiment assessing bacterial detachment by the moving meniscus and recorded as 10 s video sequences acquired at 10 frames s^−1^ to assess early bacterial interactions with crystal surfaces. After 24 h of incubation, 5 µl of DAPI (2 mg/ml stock solution) was added to each well, followed by incubation for 1 h at room temperature. After staining, the supernatant was gently removed, each well was carefully rinsed once with 1.5 ml of AU, and 1.5 ml of fresh AU was gently added to the crystals remaining at the bottom of the well. The suspensions were subsequently analyzed microscopically using phase-contrast microscopy to visualize HA and CaOx crystals and fluorescence microscopy under DAPI excitation to detect bacteria associated with crystal surfaces. Four independent biological replicates, each including three technical replicates, were performed.

### Sedimentation and autoaggregation assay

Overnight cultures of *E. coli* AAEC191A, BL21(DE3), and JM101 carrying either the Dr fimbriae plasmid pCC90 (Dr^+^) or control pACYC177 (Dr^−^) were grown in LB medium and adjusted to OD_600_ = 2.0 using the strain-specific spent culture medium obtained after centrifugation. Forty milliliters of each adjusted culture were transferred into sterile flat-bottom screw-cap tubes (25 × 145 mm) and left undisturbed at room temperature for sedimentation. Tubes were photographed at 0, 0.5, 1, 2, 2.5, 3, 3.5, 4, 10, 22, 24, 28, and 48 h using a Canon EF 50 mm f/1.4 lens under constant white-light illumination. Changes in culture density were analyzed at three positions along each tube based on grayscale luminance measurements. Luminance values were compared with control tubes containing spent culture medium from centrifuged bacterial cultures incubated identically. Differences in luminance were interpreted as changes in optical density, and the percentage of remaining OD over time was calculated. Autoaggregation and settling behavior of overnight cultures were additionally monitored by phase-contrast microscopy. Three independent biological replicates, each including three technical replicates, were performed.

### Bacteriophage efficiency of plating assay

The efficiency of plating of T7, λ vir and P1 vir bacteriophages was determined using Dr^+^ and Dr^−^ variants of *E. coli* AAEC191A and JM101. M13 bacteriophage was analyzed analogously, but only on JM101 variants due to the presence of sex pili required for phage adsorption. A volume of 100 µl of bacterial culture was mixed with 3 ml of molten LB top agar (0.5%, w/v) containing the appropriate antibiotic and poured onto LB agar plates. For assays involving P1 vir, the top agar was additionally supplemented with calcium chloride to a final concentration of 5 mM. Ten-fold serial dilutions of bacteriophage lysates were prepared in TM buffer containing 10 mM magnesium sulfate and 50 mM Tris hydrochloride (pH 7.5), and 10 µl of each dilution was spotted onto the solidified top agar surface. Plates were incubated upright overnight at 37 °C. Plaques were counted after incubation, and bacteriophage efficiency of plating was expressed as the ratio of plaque numbers obtained on Dr^+^ and Dr^−^ variants.

To examine susceptibility of the clinical strains, undiluted lysates of T7, λ vir, and P1 vir bacteriophages were spotted onto bacterial lawns of IH11128 and DR14 prepared as described above. Because no plaques were observed with any of the undiluted lysates, serial dilution analysis was not performed for these strains.

Three independent biological replicates, each including three technical replicates, were performed. Results were statistically evaluated with the test for proportion.

### Bacteriophage adsorption assay

Overnight bacterial cultures were refreshed in fresh LB medium until reaching OD_600_ of 0.3. Samples of 2.4 x 10^8^ bacteria were collected and volumes were adjusted with LB medium to 1 ml, where required, and supplemented with 5 mM MgCl_2_ and 5 mM CaCl_2_. P1 vir and λ vir lysates were added to obtain the multiplicity of infection (MOI) of 0.05. A control sample contained LB medium supplemented with salts indicated above. Samples were incubated for 30 min at 37 °C and centrifuged at 14,000 x g for 15 min at 4 °C. Phages present in supernatants were titrated on appropriate bacterial strains harboring no plasmids. The percentage of adsorption was calculated from numbers of phages present in samples containing no bacteria vs. numbers of unadsorbed phages in samples with bacteria. In the case of T7 bacteriophage, phage lysate was added to bacterial samples to obtain MOI = 0.001. Samples were incubated for 5 min at 37 °C and two 200 µl aliquots were collected from each sample. One sample was centrifuged for 1 min at 14,000 x g. 100 µl of supernatants and uncentrifuged samples were added to 3 ml of molten top LB agar (0.5%) and poured onto the LB agar plates. Plates were incubated upright overnight at 37 °C. Three independent biological replicates, each including three technical replicates, were performed. The percentage of adsorption was calculated with numbers of plaques obtained for centrifuged samples (unadsorbed phages) vs. uncentrifuged ones (total phages). Results were statistically evaluated with unpaired Student’s t-test.

### Determination of LamB abundance

To determine whether altered susceptibility to bacteriophage λ was due to steric shielding rather than changes in receptor abundance, LamB protein levels were quantified in AAEC191A Dr^+^ and Dr^−^ strains. Two unique tryptic peptides, NLIEWLPGSTIWAGK and ITLAQQWQAGDSIWSRPAIR, were analyzed by label-free LC–MS in three biological replicates per condition. No significant differences in peak areas were observed between strains for either peptide (unpaired two-tailed Student’s t-test, p > 0.05), as shown in S6 Table in S1 File and S15 Fig.

### Minimum inhibitory concentration (MIC) determination

The minimum inhibitory concentration (MIC) was determined by the broth microdilution method using 96-well microtiter plates with *E. coli* AAEC191A Dr^+^ and Dr^−^ strains. Bacterial suspensions were standardized to OD_600_ and diluted in LB broth to a final inoculum of approximately OD_600_ = 0.001. Susceptibility was tested against chloramphenicol, erythromycin, kanamycin, spectinomycin, and tetracycline, with antibiotics twofold serially diluted in LB to final ranges of 0.008–128 µg/ml (chloramphenicol), 0.05–200 µg/ml (erythromycin), 0.008–64 µg/ml (kanamycin), 0.025–100 µg/ml (spectinomycin), and 0.002–64 µg/ml (tetracycline). Equal volumes of bacterial inoculum were added to each well. Growth controls, medium sterility controls, and antibiotic-only wells were included. Plates were incubated for 20 h at 37 °C, shaken for 12 s before measurement, and OD_600_ was recorded using a 2 × 2 matrix per well (bandwidth 10 nm, 25 flashes). MIC values were defined as the lowest antibiotic concentration completely inhibiting growth. Three independent biological replicates, each including three technical replicates, were performed.

### Antibiotic susceptibility by disk diffusion assay

The susceptibility of *E. coli* AAEC191A Dr^+^ and Dr^−^ was assessed using a disk diffusion assay on a two-layer agar system. The bottom layer contained LB agar medium, while the upper layer was prepared by mixing LB broth and LB agar in a 2:1 (v/v) ratio with 100 µl of overnight bacterial culture. Antibiotics tested were chloramphenicol (34 mg/ml), erythromycin (100 mg/ml), kanamycin (20 mg/ml), spectinomycin (100 mg/ml), and tetracycline (12.5 mg/ml), with serial tenfold dilutions from 10^−1^ to 10^−8^. Sterile cellulose disks were loaded with 10 µl of antibiotic in 70% ethanol or 15 µl of aqueous solution, depending on solubility. Disks were arranged around the plate perimeter with undiluted stock in the center. Plates were incubated overnight at 37 °C. Three independent biological replicates, each including three technical replicates, were performed.

### Statistical analysis

Unless otherwise stated, all experiments were performed with three biological replicates, each including three technical replicates. Bacterial cell counts are presented as mean values ± standard deviation (SD). Unless stated otherwise, statistical significance between the analyzed groups was evaluated using the non-parametric Kruskal-Wallis test. This method was specifically chosen because cell counting yields discrete data that often do not meet the assumptions of normal distribution required for standard parametric tests. The levels of statistical significance are defined as follows: * p < 0.05, ** p < 0.01, and *** p < 0.001.

## Supporting information

S1 Rawgel Original uncropped and unadjusted blot and gel images underlying all Western blot and gel results presented in the manuscript and Supporting Information. (PDF)

S2 Rawgel Physicochemical model of bacterial transport and adhesion in a water film. (DOCX)

S1 File S1 Table. Cell surface characteritics of the Dr fimbria-producing and non-producing AAEC191A bacteria. **S2 Table.** Bacteriophage efficiency of plating. **S3 Table.** Bacteriophage adhesion to *E. coli* AAEC191A Dr^+^ and Dr^−^ strains. **S4 Table.** Summary of [M+2H]^2+^ precursor masses for synthetic peptides used in targeted analysis of the Dra protein family. **S5 Table.** Calibration curves generated for synthetic peptides corresponding to tryptic fragments of DraB, DraC, DraD, and DraE proteins. **S6 Table.** Quantitative LC-MS analysis of two tryptic peptides from the phage λ receptor protein LamB in the AAEC191A Dr^−^ and AAEC191A Dr^+^ strains.
(DOCX)

S1 Fig Relative abundance of Dr fimbriae in model and clinical Dr-positive strains. (A) Representative Western blot analysis of fimbrial fractions isolated from *E. coli* AAEC191A Dr^+^ and IH11128. Fimbrial fractions from both strains were incubated in Laemmli buffer at 25 °C and 100 °C before electrophoresis to distinguish assembled fimbrial forms from fully dissociated DraE monomers. Three independent fimbrial fraction isolations obtained from the same biological culture of IH11128 are shown as technical isolation replicates. DraE was detected using rabbit polyclonal anti-Dr antibodies followed by horseradish peroxidase-conjugated goat anti-rabbit secondary antibodies. The DraE monomer band is indicated. (B) Representative immunofluorescence analysis of overnight cultures of IH11128, AAEC191A Dr^+,^ and AAEC191A Dr^-^ using anti-Dr antibodies and FITC-conjugated secondary antibodies. For each strain, corresponding phase-contrast and fluorescence images are shown. The scale bar shown applies to all images and represents 50 μm.
(TIF)

S2 Fig Comparison of filtration times of Dr^+^ and Dr^−^ bacterial suspensions in PBS at different pH values. Filtration times of *E. coli* AAEC191A Dr^+^ and Dr^−^ suspensions in PBS buffers at pH 5.7, 7.0, and 8.0 through 0.45 µm cellulose filters. Suspensions of Dr^+^ and Dr^−^ bacteria were adjusted to OD_600_ values of 0.85 and 0.65, respectively, to obtain comparable cell numbers. The lower data points indicate the appearance of the first filter area free of suspension, whereas the upper data points indicate complete disappearance of liquid from the filter surface. Data represent mean ± SD (n = 3), statistical analysis was performed using the non-parametric Kruskal-Wallis test. For each strain, differences in filtration time across the analyzed pH conditions were not statistically significant (p > 0.05).
(TIF)

S3 Fig Cross-sectional scanning electron microscopy of cellulose filters after filtration of Dr^+^ and Dr^−^ bacterial suspensions. Representative cross-sectional scanning electron microscopy images of *E. coli* AAEC191A Dr^+^ and Dr^−^ bacterial layers formed on 0.45 µm cellulose filters after filtration of suspensions prepared in 100 mM potassium nitrate. The images show the upper bacterial layer retained on the filter surface together with the internal structure of the cellulose filter. Dr^+^ suspensions with OD_600_ values of 1.7, 0.85, and 0.43 and Dr^−^ suspensions with OD_600_ values of 1.3, 0.65, and 0.32 were used to obtain comparable cell numbers. Control images show cellulose filters after filtration of the same volume of 100 mM potassium nitrate without bacterial cells.
(TIF)

S4 Fig Transmission electron microscopy of Dr^+^ and Dr^−^ bacteria. (A, B) Representative transmission electron microscopy images of *E. coli* AAEC191A Dr^+^ bacteria after negative staining with 1.5% uranyl acetate. Panel (A) shows a continuous electron-dense surface layer surrounding the bacterial cell, corresponding to the Dr fimbrial envelope with an estimated thickness of approximately 200–300 nm. Panel (B) shows partially disrupted envelope regions in which individual fimbrial structures are visible. (C) Representative transmission electron microscopy image of Dr^−^ bacteria prepared under identical conditions.
(TIF)

S5 Fig Preparation of the Dr fimbrial envelope from the *E. coli* AAEC191A Dr^+^. (A) SDS-PAGE analysis of fimbrial fractions (FF) isolated from the AAEC191A Dr^+^ cells suspended in PBS (pH 7.4) to an OD_600_ of 3.4. To release Dr fimbriae from the cell surface, the suspensions were incubated at 65 °C for 1, 2 and 3 h. After three hours of incubation, quantitative isolation of Dr fimbriae was obtained. Fractions were incubated in Laemmli buffer at 25 and 100 °C before electrophoresis to confirm the polymeric structure of Dr fimbriae and to check for possible contamination with monomeric DraE protein. CFF, 20 times concentrated FF. (B) Size exclusion chromatography of FF on a Superdex 200 Increase 10/300 GL column. Dr fimbriae elute in the column void volume of 8.3 ml. Inset shows the SDS-PAGE analysis of the fraction corresponding to the peak in the chromatogram. M, Prestained Protein Ladder (Thermo Scientific, Cat. No. 26616).
(TIF)

S6 Fig The effect of introducing variability in fimbrial length on protein density profiles. 5x5 fimbrial patches were rebuilt, with the length of each fimbrial polymer drawn from the normal distribution centered at 142 and standard deviation set to the specified value. Profiles are analogous to those shown in Fig 2B.
(TIF)

S7 Fig Comparison of surface projections of fimbrial densities. (A) Assembled models of the fimbrial layer used to calculate density projections. (B) Density profiles of fimbrial subunits (tan to red) or of electron microscopy stain (purple and brown) as a function of distance above or below the bacterial outer membrane; -500 nm corresponds to the cylindrical axis of the bacterium. The experimental profiles, obtained from ImageJ, were offset to align the bump in density with the outer membrane. (C) Line profiles in the electron microscopy images along which signal density was evaluated.
(TIF)

S8 Fig Binding of Dr^+^ and Dr^−^ bacteria to hydroxyapatite crystals at different pH values. Representative phase-contrast (BW) and fluorescence (DAPI) images of HA crystals with DAPI-labeled *E. coli* AAEC191A Dr^+^ and Dr^−^ bacteria attached to their surfaces. *Binding* shows samples after DAPI staining followed by a single gentle wash to remove excess unbound fluorophore. *Detachment* shows bacteria remaining associated with HA after three washing cycles of the sediment combined with intensive pipetting. Bacteria–HA mixtures were prepared in PBS at pH 5.7, 7.0, and 8.0.
(TIF)

S9 Fig Binding of Dr^+^ and Dr^−^ bacteria to calcium oxalate crystals at different pH values. Representative phase-contrast (BW) and fluorescence (DAPI) images of CaOx monohydrate crystals with DAPI-labeled *E. coli* Dr^+^ and Dr^−^ bacteria attached to their surfaces. *Binding* shows samples after DAPI staining followed by a single gentle wash to remove excess unbound fluorophore. *Detachment* shows bacteria remaining associated with CaOx after three washing cycles of the sediment combined with intensive pipetting. Bacteria– CaOx mixtures were prepared in PBS at pH 5.7, 7.0, and 8.0.
(TIF)

S10 Fig Independent biological replicates showing accumulation of Dr^+^ bacteria on calcium oxalate in artificial urine. Representative phase-contrast (PC), fluorescence (DAPI), and merged (PC+DAPI) images from three independent biological experiments showing *E. coli* Dr^+^ bacteria after 24 h growth in AU (pH 5.7) in the presence of CaOx monohydrate at a final concentration of 0.05% (w/v). Overnight cultures were diluted in AU to OD_600_ = 0.025 and incubated statically at 37 °C in 24-well polystyrene plates. After incubation, bacteria associated with crystal surfaces were stained with DAPI, planktonic cells were gently removed and crystals remaining at the bottom of the wells were rinsed once with AU. Phase-contrast images show crystal morphology, fluorescence images show DAPI-labeled bacteria, and merged images present the spatial relationship between bacteria and crystal surfaces. Scale bars, 20 and 100 µm.
(TIF)

S11 Fig Independent biological replicates showing association of Dr^+^ bacteria with hydroxyapatite in artificial urine. Representative phase-contrast (PC), fluorescence (DAPI), and merged (PC+DAPI) images from three independent biological experiments showing *E. coli* Dr^+^ bacteria after 24 h growth in AU (pH 5.7) in the presence of HA at a final concentration of 0.05% (w/v). Overnight cultures were diluted in AU to OD_600_ = 0.025 and incubated statically at 37 °C in 24-well polystyrene plates. After incubation, bacteria associated with crystal surfaces were stained with DAPI, planktonic cells were gently removed and crystals remaining at the bottom of the wells were rinsed once with AU. Phase-contrast images show crystal morphology, fluorescence images show DAPI-labeled bacteria, and merged images present the spatial relationship between bacteria and crystal surfaces. Scale bars, 20 and 100 µm.
(TIF)

S12 Fig Differential stability of hydroxyapatite in PBS and citrate buffer at pH 5.7. (A) Macroscopic appearance of suspensions containing 3% HA in 6 ml of PBS (pH 5.7) or 3 mM citrate buffer (pH 5.7). Tubes were shaken for 1 h and then left undisturbed for sedimentation. The photograph shows both tubes after 1 h of sedimentation. (B) Phase-contrast microscopy images showing HA samples collected from the tubes shown in panel (A) immediately after shaking (t = 0 h) and from the supernatant above the sediment after 1 h of sedimentation (t = 1 h) in PBS or 3 mM citrate buffer (pH 5.7). Scale bars, 50 μm.
(TIF)

S13 Fig Sedimentation behavior of Dr^+^ and Dr^−^ bacterial strains under static conditions. Representative phase-contrast images of samples collected from the bottom of tubes containing overnight cultures of *E. coli* Dr^+^ and Dr^−^ variants of AAEC191A, BL21(DE3), and JM101 after 3 h incubation under static conditions. Representative settling of static bacterial suspensions (OD_600_ = 2.0) of Dr^+^ and Dr^−^ variants of AAEC191A, BL21(DE3), and JM101 recorded after 0, 2, 4, and 10 h. The LB indicates control post-culture medium obtained after centrifugation of bacterial cells. Sedimentation profiles corresponding to the three suspension layers (a–c) indicated on the LB control tube, expressed as the percentage of the initial optical density measured at t = 0 h over 48 h of incubation. Data represent mean ± SD from three independent biological replicates.
(TIF)

S14 Fig Dr fimbrial envelope limits bacteriophage access to the bacterial surface in JM101. Representative plaque assays showing lysis induced by bacteriophages P1 vir, λ vir, T7, and M13 on lawns of *E. coli* JM101 Dr^+^ and Dr^−^ bacteria. Ten-fold serial dilutions of bacteriophage lysates (10^0^–10^−8^) were spotted onto LB top agar containing bacterial cells. Images show contrast-enhanced versions highlighting plaque boundaries.
(TIF)

S15 Fig Relative quantification of the phage λ receptor protein LamB in *E. coli* AAEC191A Dr^−^ and AAEC191A Dr^+^ strains. Boxplots represent LC-MS peak areas (cpSs) for two unique tryptic peptides—p1: NLIEWLPGSTIWAGK and p2: ITLAQQWQAGDSIWSRPAIR—acquired from three biological replicates per condition. Peptide intensities were normalized to the number of cells subjected to enzymatic digestion, as described in the proteomic sample preparation section of the manuscript. Statistical comparison using an unpaired two-tailed Student’s t-test revealed no significant differences between the strains for either peptide (p > 0.05).
(TIF)

S16 Fig Dr fimbrial envelope does not alter antibiotic susceptibility of *E. coli* AAEC191A. (A) Representative disk diffusion assays comparing susceptibility of *E. coli* AAEC191A Dr^+^ and Dr^−^ cells to chloramphenicol, erythromycin, kanamycin, spectinomycin, and tetracycline. Serial ten-fold dilutions of each antibiotic were applied to cellulose disks placed on two-layer agar plates seeded with bacterial cells. Plates were incubated overnight at 37 °C. (B) Growth inhibition profiles obtained by broth microdilution assays for the same antibiotics. Graphs show OD_600_ values of Dr^+^ and Dr^−^ bacterial cultures as a function of antibiotic concentration after overnight incubation under static conditions at 37 °C. Data represent mean ± SD from three independent biological replicates.
(TIF)

S17 Fig Representative susceptibility of the clinical UPEC strains IH11128 and DR14 to T7, λ vir, and P1 vir bacteriophages. Undiluted lysates (10 µl) were spotted onto bacterial lawns. No plaques were observed after overnight incubation at 37 °C.
(TIF)

S18 Fig Calibration curves for synthetic tryptic peptides derived from DraB, DraC, DraD, and DraE proteins. Each curve represents the linear relationship between the known concentration of the synthetic peptide standard and the corresponding chromatographic peak area extracted from the EIC (Extracted Ion Chromatogram) at a defined m/z window (±0.02 Da). The peptides analyzed include SPAPFVVTPPLFR (DraB, m/z 714.4059), IEEYPALFR (DraC, m/z 569.3006), TGGDGWSPVK (DraD, m/z 502.2458), and FFLISDNNR (DraE, m/z 563.2881). All measurements were performed in triplicates using the ZenoTOF 7600 mass spectrometer, and retention times were used to confirm identity and ensure consistency. The curves exhibit strong linearity (R2>0.98) across the tested concentration range and served as the basis for absolute quantification of target proteins in biological samples.
(TIF)

S1 Video A dynamic 3D visualization of the cylindrical sections of the Dr envelope. First, a 25 x 25 patch of DraE fimbriae, each containing 142 subunits, was built using a Monte Carlo procedure using dimer geometries sampled in atomistic molecular dynamics. The patch was transformed and repeated to form a ring, and additional translations along the cylindrical axis were applied for visualization purposes.
(MP4)

S2 Video Differential resistance of AAEC191A Dr^+^ and Dr^−^ bacteria attached to glass during passage of a receding meniscus. Representative time-lapse phase-contrast recordings showing detachment of surface-adhered Dr^+^ and Dr^−^ bacteria from glass by a receding liquid meniscus. Panels correspond to the conditions shown in Fig 6: (A) Dr^+^ bacteria attached to glass and (B) Dr^−^ bacteria attached to glass. Labels indicate air (a), meniscus (m), and bacteria (b). Recordings were acquired for 1 min at 5 frames per second. The video illustrates efficient detachment of Dr^−^ cells by the receding meniscus, whereas many Dr^+^ cells remain attached after meniscus passage.
(MP4)

S3 Video Detachment of Dr^+^ and Dr^−^ bacteria from untreated polystyrene by a receding meniscus. Representative time-lapse phase-contrast recordings showing the behavior of surface-adhered AAEC191A Dr^+^ and Dr^−^ bacteria on untreated polystyrene during passage of a receding liquid meniscus. Panels correspond to the conditions shown in Fig 6: (A) Dr^+^ bacteria attached to untreated polystyrene and (B) Dr^−^ bacteria attached to untreated polystyrene. Labels indicate air (a), meniscus (m), and bacteria (b). Recordings were acquired for 80 sec at 5 frames per second. Both bacterial variants were detached from untreated polystyrene by capillary forces generated by the receding meniscus. In the Dr^+^ sample, detached cells were immediately transported within the meniscus and accumulated at the wetting arm contacting the upper glass coverslip, where they adhered efficiently, resulting in visible bacterial accumulation behind the receding meniscus.
(MP4)

S4 Video Dynamic association of Dr^+^ *E. coli* with calcium oxalate crystals in artificial urine. Representative time-lapse phase-contrast recordings showing interactions of Dr^+^ *E. coli* AAEC191A with CaOx monohydrate crystals in AU (pH 5.7). Overnight cultures were diluted in AU to OD_600_ = 0.025, supplemented with CaOx to a final concentration of 0.05% (w/v), and incubated under static conditions at 37 °C. The video shows four independent biological experiments recorded after 2 h of incubation. Images were acquired for 10 s at 0.1 s intervals using phase-contrast microscopy. Bacteria display characteristic polar attachment to CaOx crystals, forming radial arrangements around crystal surfaces. Collective bacterial motility results in visible oscillations and occasional displacement of crystals along the bottom surface. Recordings were acquired using an Olympus IX73 inverted microscope equipped with a UPlanFL N 20×/0.30 objective and a Hamamatsu Photonics ORCA-Flash2.8 camera.
(MP4)

S5 Video Control conditions showing absence of specific crystal-associated behavior outside the Dr^+^–CaOx interaction. The four panels show Dr^+^ *E. coli* AAEC191A in the presence of HA, Dr^−^ *E. coli* AAEC191A in the presence of CaOx monohydrate, Dr^−^ *E. coli* AAEC191A in the presence of HA, and control cultures of Dr^+^ and Dr^−^ bacteria in AU without added minerals. No characteristic radial attachment, crystal oscillation, or crystal displacement was observed under these conditions. Experimental conditions and recording parameters were identical to those described in S4 Video.
(MP4)

## References

[1] Yang X, Chen H, Zheng Y, Qu S, Wang H, Yi F. Disease burden and long-term trends of urinary tract infections: A worldwide report. Front Public Health. 2022;10:888205. doi: 10.3389/fpubh.2022.888205 35968451 PMC9363895

[2] Flores-Mireles AL, Walker JN, Caparon M, Hultgren SJ. Urinary tract infections: epidemiology, mechanisms of infection and treatment options. Nat Rev Microbiol. 2015;13(5):269–84. doi: 10.1038/nrmicro3432 25853778 PMC4457377

[3] Morgan CJ, Atkins H, Wolfe AJ, Brubaker L, Aslam S, Putonti C, et al. Phage Therapy for Urinary Tract Infections: Progress and Challenges Ahead. Int Urogynecol J. 2025;36(7):1343–53. doi: 10.1007/s00192-025-06136-8 40358692 PMC12356746

[4] Forsyth VS, Armbruster CE, Smith SN, Pirani A, Springman AC, Walters MS. Rapid growth of uropathogenic Escherichia coli during human urinary tract infection. mBio. 2018;9(2):e00186-18. doi: 10.1128/mBio.00186-18 PMC5844997 29511075

[5] Frick-Cheng AE, Sintsova A, Smith SN, Krauthammer M, Eaton KA, Mobley HLT. The gene expression profile of uropathogenic Escherichia coli in women with uncomplicated urinary tract infections is recapitulated in the mouse model. mBio. 2020;11(4):e01412-20. doi: 10.1128/mBio.01412-20 PMC7439467 32788379

[6] Phan M-D, Schirra HJ, Nhu NTK, Peters KM, Sarkar S, Allsopp LP, et al. Combined functional genomic and metabolomic approaches identify new genes required for growth in human urine by multidrug-resistant Escherichia coli ST131. mBio. 2024;15(3):e0338823. doi: 10.1128/mbio.03388-23 38353545 PMC10936160

[7] Banerjee R, Johnston B, Lohse C, Chattopadhyay S, Tchesnokova V, Sokurenko EV, et al. The clonal distribution and diversity of extraintestinal Escherichia coli isolates vary according to patient characteristics. Antimicrob Agents Chemother. 2013;57(12):5912–7. doi: 10.1128/AAC.01065-13 24041881 PMC3837911

[8] Nicolas-Chanoine MH, Blanco J, Leflon-Guibout V, Demarty R, Alonso MP, Canica MM. Intercontinental emergence of Escherichia coli clone O25:H4-ST131 producing CTX-M-15. J Antimicrob Chemother. 2007;61(2):273–81. doi: 10.1093/jac/dkm464 18077311

[9] Manges AR, Geum HM, Guo A, Edens TJ, Fibke CD, Pitout JDD. Global Extraintestinal Pathogenic Escherichia coli (ExPEC) Lineages. Clin Microbiol Rev. 2019;32(3):e00135-18. doi: 10.1128/CMR.00135-18 31189557 PMC6589867

[10] Petty NK, Ben Zakour NL, Stanton-Cook M, Skippington E, Totsika M, Forde BM, et al. Global dissemination of a multidrug resistant Escherichia coli clone. Proc Natl Acad Sci U S A. 2014;111(15):5694–9. doi: 10.1073/pnas.1322678111 24706808 PMC3992628

[11] Gibreel TM, Dodgson AR, Cheesbrough J, Fox AJ, Bolton FJ, Upton M. Population structure, virulence potential and antibiotic susceptibility of uropathogenic Escherichia coli from Northwest England. J Antimicrob Chemother. 2012;67(2):346–56. doi: 10.1093/jac/dkr451 22028202

[12] Alvarez-Fraga L, Phan MD, Goh KGK, Nhu NTK, Hancock SJ, Allsopp LP, et al. Differential Afa/Dr fimbriae expression in the multidrug-resistant Escherichia coli ST131 clone. mBio. 2022;13(1):e03519-21. doi: 10.1128/mbio.03519-21 PMC8764528 35038925

[13] Servin AL. Pathogenesis of Afa/Dr diffusely adhering Escherichia coli. Clin Microbiol Rev. 2005;18(2):264–92. doi: 10.1128/CMR.18.2.264-292.2005 15831825 PMC1082796

[14] Nowicki B, Selvarangan R, Nowicki S. Family of Escherichia coli Dr adhesins: decay-accelerating factor receptor recognition and invasiveness. J Infect Dis. 2001;183 Suppl 1:S24-7. doi: 10.1086/318846 11171008

[15] Väisänen-Rhen V. Fimbria-like hemagglutinin of Escherichia coli O75 strains. Infect Immun. 1984;46(2):401–7. doi: 10.1128/iai.46.2.401-407.1984 6150006 PMC261546

[16] Anderson KL, Billington J, Pettigrew D, Cota E, Simpson P, Roversi P, et al. An Atomic Resolution Model for Assembly, Architecture, and Function of the Dr Adhesins. Mol Cell. 2004;15(4):647–57. doi: 10.1016/j.molcel.2004.08.003 15327779

[17] Piatek R, Zalewska B, Kolaj O, Ferens M, Nowicki B, Kur J. Molecular aspects of biogenesis of Escherichia coli Dr Fimbriae: characterization of DraB-DraE complexes. Infect Immun. 2005;73(1):135–45. doi: 10.1128/IAI.73.1.135-145.2005 15618148 PMC538934

[18] Cota E, Jones C, Simpson P, Altroff H, Anderson KL, du Merle L, et al. The solution structure of the invasive tip complex from Afa/Dr fibrils. Mol Microbiol. 2006;62(2):356–66. doi: 10.1111/j.1365-2958.2006.05375.x 16965519 PMC2628978

[19] Jedrzejczak R, Dauter Z, Dauter M, Piatek R, Zalewska B, Mróz M, et al. Structure of DraD invasin from uropathogenic Escherichia coli: a dimer with swapped beta-tails. Acta Crystallogr D Biol Crystallogr. 2006;62(Pt 2):157–64. doi: 10.1107/S0907444905036747 16421447

[20] Pettigrew D, Anderson KL, Billington J, Cota E, Simpson P, Urvil P, et al. High resolution studies of the Afa/Dr adhesin DraE and its interaction with chloramphenicol. J Biol Chem. 2004;279(45):46851–7. doi: 10.1074/jbc.M409284200 15331605

[21] Nowicki B, Moulds J, Hull R, Hull S. A hemagglutinin of uropathogenic Escherichia coli recognizes the Dr blood group antigen. Infect Immun. 1988;56(5):1057–60. doi: 10.1128/iai.56.5.1057-1060.1988 2895740 PMC259761

[22] Westerlund B, Kuusela P, Risteli J, Risteli L, Vartio T, Rauvala H, et al. The O75X adhesin of uropathogenic Escherichia coli is a type IV collagen-binding protein. Mol Microbiol. 1989;3(3):329–37. doi: 10.1111/j.1365-2958.1989.tb00178.x 2568575

[23] Guignot J, Peiffer I, Bernet-Camard MF, Lublin DM, Carnoy C, Moseley SL, et al. Recruitment of CD55 and CD66e brush border-associated glycosylphosphatidylinositol-anchored proteins by members of the Afa/Dr diffusely adhering family of Escherichia coli that infect the human polarized intestinal Caco-2/TC7 cells. Infect Immun. 2000;68(6):3554–63. doi: 10.1128/IAI.68.6.3554-3563.2000 10816511 PMC97642

[24] Berger CN, Billker O, Meyer TF, Servin AL, Kansau I. Differential recognition of members of the carcinoembryonic antigen family by Afa/Dr adhesins of diffusely adhering Escherichia coli (Afa/Dr DAEC). Mol Microbiol. 2004;52(4):963–83. doi: 10.1111/j.1365-2958.2004.04033.x 15130118

[25] Zalewska-Piątek B, Olszewski M, Lipniacki T, Błoński S, Wieczór M, Bruździak P, et al. A shear stress micromodel of urinary tract infection by the Escherichia coli producing Dr adhesin. PLoS Pathog. 2020;16(1):e1008247. doi: 10.1371/journal.ppat.1008247 31917805 PMC7004390

[26] Zavialov A, Zav’yalova G, Korpela T, Zav’yalov V. FGL chaperone-assembled fimbrial polyadhesins: anti-immune armament of Gram-negative bacterial pathogens. FEMS Microbiol Rev. 2007;31(4):478–514. doi: 10.1111/j.1574-6976.2007.00075.x 17576202

[27] Zavialov AV, Berglund J, Pudney AF, Fooks LJ, Ibrahim TM, MacIntyre S, et al. Structure and Biogenesis of the Capsular F1 Antigen from Yersinia pestis. Cell. 2003;113(5):587–96. doi: 10.1016/s0092-8674(03)00351-9 12787500

[28] Lindler LE, Tall BD. Yersinia pestis pH 6 antigen forms fimbriae and is induced by intracellular association with macrophages. Mol Microbiol. 1993;8(2):311–24. doi: 10.1111/j.1365-2958.1993.tb01575.x 8100346

[29] Pakharukova N, Roy S, Tuittila M, Rahman MM, Paavilainen S, Ingars A-K, et al. Structural basis for Myf and Psa fimbriae-mediated tropism of pathogenic strains of Yersinia for host tissues. Mol Microbiol. 2016;102(4):593–610. doi: 10.1111/mmi.13481 27507539

[30] Iriarte M, Vanooteghem JC, Delor I, Díaz R, Knutton S, Cornelis GR. The Myf fibrillae of Yersinia enterocolitica. Mol Microbiol. 1993;9(3):507–20. doi: 10.1111/j.1365-2958.1993.tb01712.x 8105362

[31] Roy SP, Rahman MM, Yu XD, Tuittila M, Knight SD, Zavialov AV. Crystal structure of enterotoxigenic Escherichia coli colonization factor CS6 reveals a novel type of functional assembly. Mol Microbiol. 2012;86(5):1100–15. doi: 10.1111/mmi.12044 23046340

[32] Nataro JP, Kaper JB. Diarrheagenic Escherichia coli. Clin Microbiol Rev. 1998;11(1):142–201. doi: 10.1128/CMR.11.1.142 9457432 PMC121379

[33] Berry AA, Yang Y, Pakharukova N, Garnett JA, Lee W chao, Cota E, et al. Structural insight into host recognition by aggregative adherence fimbriae of enteroaggregative Escherichia coli. PLoS Pathog. 2014;10(9):e1004404. doi: 10.1371/journal.ppat.1004404 PMC4169507 25232738

[34] Salih O, Remaut H, Waksman G, Orlova EV. Structural analysis of the Saf pilus by electron microscopy and image processing. J Mol Biol. 2008;379(1):174–87. doi: 10.1016/j.jmb.2008.03.056 18448124

[35] Hahn E, Wild P, Hermanns U, Sebbel P, Glockshuber R, Häner M. Exploring the 3D Molecular Architecture of Escherichia coli Type 1 Pili. J Mol Biol. 2002;323(5):845–57. doi: 10.1016/S0022-2836(02)01005-7 12417198

[36] Hospenthal MK, Redzej A, Dodson K, Ukleja M, Frenz B, Rodrigues C, et al. Structure of a Chaperone-Usher Pilus Reveals the Molecular Basis of Rod Uncoiling. Cell. 2016;164(1–2):269–78. doi: 10.1016/j.cell.2015.11.049 26724865 PMC4715182

[37] Sokurenko EV, Vogel V, Thomas WE. Catch-bond mechanism of force-enhanced adhesion: counterintuitive, elusive, but . . . widespread? Cell Host Microbe. 2008;4(4):314–23. doi: 10.1016/j.chom.2008.09.005 18854236 PMC2610669

[38] Sauer MM, Jakob RP, Eras J, Baday S, Eriş D, Navarra G, et al. Catch-bond mechanism of the bacterial adhesin FimH. Nat Commun. 2016;7:10738. doi: 10.1038/ncomms10738 26948702 PMC4786642

[39] Thomas WE, Nilsson LM, Forero M, Sokurenko EV, Vogel V. Shear-dependent “stick-and-roll” adhesion of type 1 fimbriated Escherichia coli. Mol Microbiol. 2004;53(5):1545–57. doi: 10.1111/j.1365-2958.2004.04226.x 15387828

[40] Nilsson LM, Thomas WE, Sokurenko EV, Vogel V. Elevated shear stress protects Escherichia coli cells adhering to surfaces via catch bonds from detachment by soluble inhibitors. Appl Environ Microbiol. 2006;72(4):3005–10. doi: 10.1128/AEM.72.4.3005-3010.2006 16598008 PMC1449047

[41] Anderson BN, Ding AM, Nilsson LM, Kusuma K, Tchesnokova V, Vogel V, et al. Weak rolling adhesion enhances bacterial surface colonization. J Bacteriol. 2007;189(5):1794–802. doi: 10.1128/JB.00899-06 17189376 PMC1855705

[42] McLay RB, Nguyen HN, Jaimes-Lizcano YA, Dewangan NK, Alexandrova S, Rodrigues DF, et al. Level of Fimbriation Alters the Adhesion of Escherichia coli Bacteria to Interfaces. Langmuir. 2017;34(3):1133–42. doi: 10.1021/acs.langmuir.7b02447 28976770

[43] Otto K, Elwing H, Hermansson M. The role of type 1 fimbriae in adhesion of Escherichia coli to hydrophilic and hydrophobic surfaces. Colloids Surf B Biointerfaces. 1999;15(1):99–111. doi: 10.1016/S0927-7765(99)00050-8

[44] Iyer D, Gulyuk AV, Reddy P, Kirste R, Collazo R, LaJeunesse DR, et al. Behavior of E. coli with Variable Surface Morphology Changes on Charged Semiconductor Interfaces. ACS Appl Bio Mater. 2019;2(9):4044–51. doi: 10.1021/acsabm.9b00573 35021338 PMC10167750

[45] Hasman H, Schembri MA, Klemm P. Antigen 43 and type 1 fimbriae determine colony morphology of Escherichia coli K-12. J Bacteriol. 2000;182(4):1089–95. doi: 10.1128/JB.182.4.1089-1095.2000 10648536 PMC94386

[46] Hasman H, Chakraborty T, Klemm P. Antigen-43-mediated autoaggregation of Escherichia coli is blocked by fimbriation. J Bacteriol. 1999;181(16):4834–41. doi: 10.1128/JB.181.16.4834-4841.1999 PMC93969 10438752

[47] Sherlock O, Schembri MA, Reisner A, Klemm P. Novel roles for the AIDA adhesin from diarrheagenic Escherichia coli: cell aggregation and biofilm formation. J Bacteriol. 2004;186(23):8058–65. doi: 10.1128/JB.186.23.8058-8065.2004 15547278 PMC529095

[48] Sherlock O, Vejborg RM, Klemm P. The TibA adhesin/invasin from enterotoxigenic Escherichia coli is self recognizing and induces bacterial aggregation and biofilm formation. Infect Immun. 2005;73(4):1954–63. doi: 10.1128/IAI.73.4.1954-1963.2005 15784535 PMC1087433

[49] Wells TJ, Sherlock O, Rivas L, Mahajan A, Beatson SA, Torpdahl M, et al. EhaA is a novel autotransporter protein of enterohemorrhagic Escherichia coli O157:H7 that contributes to adhesion and biofilm formation. Environ Microbiol. 2008;10(3):589–604. doi: 10.1111/j.1462-2920.2007.01479.x 18237301

[50] Pratt LA, Kolter R. Genetic analysis of Escherichia coli biofilm formation: roles of flagella, motility, chemotaxis and type I pili. Mol Microbiol. 1998;30(2):285–93. doi: 10.1046/j.1365-2958.1998.01061.x 9791174

[51] Schembri MA, Blom J, Krogfelt KA, Klemm P. Capsule and fimbria interaction in Klebsiella pneumoniae. Infect Immun. 2005;73(8):4626–33. doi: 10.1128/IAI.73.8.4626-4633.2005 16040975 PMC1201234

[52] Carnoy C, Moseley SL. Mutational analysis of receptor binding mediated by the Dr family of Escherichia coli adhesins. Mol Microbiol. 1997;23(2):365–79. doi: 10.1046/j.1365-2958.1997.2231590.x 9044270

[53] Nagórka M, Zalewska-Piątek B, Iwanicki A, Wityk P, Jakiela S, Świtlik W, et al. Dataset for: The biophysical and functional characterization of the Dr fimbrial envelope in E. coli suggests its importance for pathogenesis and lithogenesis [Internet]. Zenodo; 2025 [cited 2026 Jul 29]. Available from: https://zenodo.org/doi/10.5281/zenodo.15720129 10.1371/journal.ppat.1014588 PMC13608002 42748165

[54] Yang F, Wang L, Zhou J, Xiao H, Liu H. In Situ Structures of the Ultra-Long Extended and Contracted Tail of Myoviridae Phage P1. Viruses. 2023;15(6):1267. doi: 10.3390/v15061267 37376567 PMC10304247

[55] Ge X, Wang J. Structural mechanism of bacteriophage lambda tail’s interaction with the bacterial receptor. Nat Commun. 2024;15(1):4185. doi: 10.1038/s41467-024-48686-3 38760367 PMC11101478

[56] Hu B, Margolin W, Molineux IJ, Liu J. The bacteriophage T7 virion undergoes extensive structural remodeling during infection. Science. 2013;339(6119):576–9. doi: 10.1126/science.1231887 PMC3873743 23306440

[57] Werts C, Michel V, Hofnung M, Charbit A. Adsorption of bacteriophage lambda on the LamB protein of Escherichia coli K-12: point mutations in gene J of lambda responsible for extended host range. J Bacteriol. 1994;176(4):941–7. doi: 10.1128/jb.176.4.941-947.1994 8106335 PMC205142

[58] Franklin NC. Mutation in gal U gene of E. coli blocks phage P1 infection. Virology. 1969;38(1):189–91. doi: 10.1016/0042-6822(69)90144-5 4891220

[59] Marvin DA, Hohn B. Filamentous bacterial viruses. Bacteriol Rev. 1969;33(2):172–209. doi: 10.1128/br.33.2.172-209.1969 4979697 PMC378320

[60] Knecht LE, Veljkovic M, Fieseler L. Diversity and function of phage encoded depolymerases. Front Microbiol. 2020;10:2949. doi: 10.3389/fmicb.2019.02949 PMC6966330 31998258

[61] Scholl D, Adhya S, Merril C. Escherichia coli K1’s capsule is a barrier to bacteriophage T7. Appl Environ Microbiol. 2005;71(8):4872–4. doi: 10.1128/AEM.71.8.4872-4874.2005 16085886 PMC1183359

[62] Wawer J, Chrzanowska N, Aranowski R, Narajczyk M, Iwanicki A, Nagórka M, et al. Determination of the apparent volume of bacteria using example of E. coli strain producing Dr fimbriae. J Mol Liq. 2025;438:128698. doi: 10.1016/j.molliq.2025.128698

[63] Labigne-Roussel AF, Lark D, Schoolnik G, Falkow S. Cloning and expression of an afimbrial adhesin (AFA-I) responsible for P blood group-independent, mannose-resistant hemagglutination from a pyelonephritic Escherichia coli strain. Infect Immun. 1984;46(1):251–9. doi: 10.1128/iai.46.1.251-259.1984 6148308 PMC261465

[64] Goldhar J, Perry R, Golecki JR, Hoschutzky H, Jann B, Jann K. Nonfimbrial, mannose-resistant adhesins from uropathogenic Escherichia coli O83:K1:H4 and O14:K?:H11. Infect Immun. 1987;55(8):1837–42. doi: 10.1128/iai.55.8.1837-1842.1987 2886432 PMC260610

[65] Bilge SS, Clausen CR, Lau W, Moseley SL. Molecular characterization of a fimbrial adhesin, F1845, mediating diffuse adherence of diarrhea-associated Escherichia coli to HEp-2 cells. J Bacteriol. 1989;171(8):4281–9. doi: 10.1128/jb.171.8.4281-4289.1989 2568985 PMC210202

[66] Pham TQ, Goluszko P, Popov V, Nowicki S, Nowicki BJ. Molecular cloning and characterization of Dr-II, a nonfimbrial adhesin-I-like adhesin isolated from gestational pyelonephritis-associated Escherichia coli that binds to decay-accelerating factor. Infect Immun. 1997;65(10):4309–18. doi: 10.1128/iai.65.10.4309-4318.1997 9317041 PMC175617

[67] Keller R, Ordoñez JG, de Oliveira RR, Trabulsi LR, Baldwin TJ, Knutton S. Afa, a diffuse adherence fibrillar adhesin associated with enteropathogenic Escherichia coli. Infect Immun. 2002;70(5):2681–9. doi: 10.1128/IAI.70.5.2681-2689.2002 11953412 PMC127892

[68] Korotkova N, Le Trong I, Samudrala R, Korotkov K, Van Loy CP, Bui A-L, et al. Crystal structure and mutational analysis of the DaaE adhesin of Escherichia coli. J Biol Chem. 2006;281(31):22367–77. doi: 10.1074/jbc.M604646200 16751628

[69] Daudon M, Donsimoni R, Hennequin C, Fellahi S, Le Moel G, Paris M, et al. Sex- and age-related composition of 10 617 calculi analyzed by infrared spectroscopy. Urol Res. 1995;23(5):319–26. doi: 10.1007/BF00300021 8839389

[70] Pak CYC, Poindexter JR, Adams-Huet B, Pearle MS. Predictive value of kidney stone composition in the detection of metabolic abnormalities. Am J Med. 2003;115(1):26–32. doi: 10.1016/s0002-9343(03)00201-8 12867231

[71] Lai H-C, Chang S-N, Lin H-C, Hsu Y-L, Wei H-M, Kuo C-C, et al. Association between urine pH and common uropathogens in children with urinary tract infections. J Microbiol Immunol Infect. 2021;54(2):290–8. doi: 10.1016/j.jmii.2019.08.002 31604680

[72] Liu Y, Qu M, Carter RE, Leng S, Ramirez-Giraldo JC, Jaramillo G. Differentiating calcium oxalate and hydroxyapatite stones in vivo using dual-energy CT and urine supersaturation and pH values. Acad Radiol. 2013;20(12):1521–5. doi: 10.1016/j.acra.2013.08.018 PMC3963806 24200478

[73] Stamatelou K, Goldfarb DS. Epidemiology of Kidney Stones. Healthcare. 2023;11(3):424. doi: 10.3390/healthcare11030424 PMC9914194 36766999

[74] Kravdal G, Helgø D, Moe MK. Kidney stone compositions and frequencies in a Norwegian population. Scand J Urol. 2019;53(2–3):139–44. doi: 10.1080/21681805.2019.1606031 31070078

[75] Singh P, Enders FT, Vaughan LE, Bergstralh EJ, Knoedler JJ, Krambeck AE, et al. Stone Composition Among First-Time Symptomatic Kidney Stone Formers in the Community. Mayo Clin Proc. 2015;90(10):1356–65. doi: 10.1016/j.mayocp.2015.07.016 26349951 PMC4593754

[76] Grases F, Costa-Bauzá A, Julià F, Isern B, Guimerà J, Bauzá-Quetglas JL, et al. Evidence of bacterial imprints in different types of non-struvite kidney stones. BMC Urol. 2025;25(1):63. doi: 10.1186/s12894-025-01755-1 40155959 PMC11951595

[77] Tavichakorntrakool R, Prasongwattana V, Sungkeeree S, Saisud P, Sribenjalux P, Pimratana C, et al. Extensive characterizations of bacteria isolated from catheterized urine and stone matrices in patients with nephrolithiasis. Nephrol Dial Transplant. 2012;27(11):4125–30. doi: 10.1093/ndt/gfs057 22461670

[78] Zhong F, Wu W, Chen D, Lai Y, Tiselius H-G, Jiang C, et al. The characteristic and relationship of Escherichia coli isolated from urine and stones in patients with calcium oxalate stones. Urolithiasis. 2021;49(5):407–14. doi: 10.1007/s00240-021-01243-9 33454825

[79] Barr-Beare E, Saxena V, Hilt EE, Thomas-White K, Schober M, Li B, et al. The Interaction between Enterobacteriaceae and Calcium Oxalate Deposits. PLoS One. 2015;10(10):e0139575. doi: 10.1371/journal.pone.0139575 26448465 PMC4598009

[80] Schmidt WC, Mousavi A, Li J, Yang R, Gonzalez Marin G, Schreiber HL. Intercalated bacterial biofilms are intrinsic internal components of calcium-based kidney stones. Proc Natl Acad Sci USA. 2026;123(5):e2517066123. doi: 10.1073/pnas.2517066123 PMC12867757 41587311

[81] Amimanan P, Tavichakorntrakool R, Fong-Ngern K, Sribenjalux P, Lulitanond A, Prasongwatana V, et al. Elongation factor Tu on Escherichia coli isolated from urine of kidney stone patients promotes calcium oxalate crystal growth and aggregation. Sci Rep. 2017;7(1):2953. doi: 10.1038/s41598-017-03213-x 28592876 PMC5462744

[82] Chutipongtanate S, Sutthimethakorn S, Chiangjong W, Thongboonkerd V. Bacteria can promote calcium oxalate crystal growth and aggregation. J Biol Inorg Chem. 2013;18(3):299–308. doi: 10.1007/s00775-012-0974-0 23334195

[83] Khan SR, Glenton PA, Backov R, Talham DR. Presence of lipids in urine, crystals and stones: implications for the formation of kidney stones. Kidney Int. 2002;62(6):2062–72. doi: 10.1046/j.1523-1755.2002.00676.x 12427130

[84] Chutipongtanate S, Thongboonkerd V. Red blood cell membrane fragments but not intact red blood cells promote calcium oxalate monohydrate crystal growth and aggregation. J Urol. 2010;184(2):743–9. doi: 10.1016/j.juro.2010.03.107 20639050

[85] Kim KM. Mulberry particles formed by red blood cells in human weddelite stones. J Urol. 1983;129(4):855–7. doi: 10.1016/s0022-5347(17)52399-x 6842724

[86] Kok DJ, Papapoulos SE, Bijvoet OL. Crystal agglomeration is a major element in calcium oxalate urinary stone formation. Kidney Int. 1990;37(1):51–6. doi: 10.1038/ki.1990.7 2299809

[87] Robertson WG. Do “inhibitors of crystallisation” play any role in the prevention of kidney stones? A critique. Urolithiasis. 2017;45(1):43–56. doi: 10.1007/s00240-016-0953-y 27900407

[88] Robinson TE, Hughes EAB, Wiseman OJ, Stapley SA, Cox SC, Grover LM. Hexametaphosphate as a potential therapy for the dissolution and prevention of kidney stones. J Mater Chem B. 2020;8(24):5215–24. doi: 10.1039/d0tb00343c 32436557

[89] Wang J, Ma W, Wang X. Insights into the structure of Escherichia coli outer membrane as the target for engineering microbial cell factories. Microb Cell Fact. 2021;20(1):73. doi: 10.1186/s12934-021-01565-8 33743682 PMC7980664

[90] Dolgalev GV, Safonov TA, Arzumanian VA, Kiseleva OI, Poverennaya EV. Estimating Total Quantitative Protein Content in Escherichia coli, Saccharomyces cerevisiae, and HeLa Cells. Int J Mol Sci. 2023;24(3):2081. doi: 10.3390/ijms24032081 PMC9916689 36768409

[91] Dessartine MM, Kosta A, Doan T, Cascales É, Côté JP. Type 1 fimbriae-mediated collective protection against type 6 secretion system attacks. mBio. 2024;15(4):e02553-23. doi: 10.1128/mbio.02553-23 PMC11005336 38497656

[92] Du Y, Rosqvist R, Forsberg A. Role of fraction 1 antigen of Yersinia pestis in inhibition of phagocytosis. Infect Immun. 2002;70(3):1453–60. doi: 10.1128/IAI.70.3.1453-1460.2002 11854232 PMC127752

[93] Peters DT, Reifs A, Alonso-Caballero A, Madkour A, Waller H, Kenny B. Unraveling the molecular determinants of the anti-phagocytic protein cloak of plague bacteria. PLoS Pathog. 2022;18(3):e1010447. doi: 10.1371/journal.ppat.1010447 PMC9004762 35358289

[94] Nowicki B, Holthöfer H, Saraneva T, Rhen M, Väisänen-Rhen V, Korhonen TK. Location of adhesion sites for P-fimbriated and for 075X-positive Escherichia coli in the human kidney. Microb Pathog. 1986;1(2):169–80. doi: 10.1016/0882-4010(86)90019-7 2907770

[95] Nowicki B, Barrish JP, Korhonen T, Hull RA, Hull SI. Molecular cloning of the Escherichia coli O75X adhesin. Infect Immun. 1987;55(12):3168–73. doi: 10.1128/iai.55.12.3168-3173.1987 2890586 PMC260044

[96] Goluszko P, Moseley SL, Truong LD, Kaul A, Williford JR, Selvarangan R. Development of experimental model of chronic pyelonephritis with Escherichia coli O75:K5:H-bearing Dr fimbriae: mutation in the dra region prevented tubulointerstitial nephritis. J Clin Invest. 1997;99(7):1662–72. doi: 10.1172/JCI119329 PMC507986 9120010

[97] Blomfield IC, McClain MS, Eisenstein BI. Type 1 fimbriae mutants of Escherichia coli K12: characterization of recognized afimbriate strains and construction of new fim deletion mutants. Mol Microbiol. 1991;5(6):1439–45. doi: 10.1111/j.1365-2958.1991.tb00790.x 1686292

[98] Sklodowska K, Debski PR, Michalski JA, Korczyk PM, Dolata M, Zajac M, et al. Simultaneous Measurement of Viscosity and Optical Density of Bacterial Growth and Death in a Microdroplet. Micromachines (Basel). 2018;9(5):251. doi: 10.3390/mi9050251 30424184 PMC6187717

[99] Sali A, Blundell TL. Comparative protein modelling by satisfaction of spatial restraints. J Mol Biol. 1993;234(3):779–815. doi: 10.1006/jmbi.1993.1626 8254673

[100] Lindorff-Larsen K, Piana S, Palmo K, Maragakis P, Klepeis JL, Dror RO, et al. Improved side-chain torsion potentials for the Amber ff99SB protein force field. Proteins. 2010;78(8):1950–8. doi: 10.1002/prot.22711 20408171 PMC2970904

[101] Abraham MJ, Murtola T, Schulz R, Páll S, Smith JC, Hess B, et al. GROMACS: High performance molecular simulations through multi-level parallelism from laptops to supercomputers. SoftwareX. 2015;1–2:19–25. doi: 10.1016/j.softx.2015.06.001

[102] McGibbon RT, Beauchamp KA, Harrigan MP, Klein C, Swails JM, Hernández CX, et al. MDTraj: A Modern Open Library for the Analysis of Molecular Dynamics Trajectories. Biophys J. 2015;109(8):1528–32. doi: 10.1016/j.bpj.2015.08.015 26488642 PMC4623899

[103] Humphrey W, Dalke A, Schulten K. VMD: visual molecular dynamics. J Mol Graph. 1996;14(1):33–8, 27–8. doi: 10.1016/0263-7855(96)00018-5 8744570

[104] Wieczór M, Czub J, Orozco M. Gromologist: A GROMACS-oriented utility library for structure and topology manipulation. SoftwareX. 2025;30:102118. doi: 10.1016/j.softx.2025.102118

[105] Van Oss CJ, Ju L, Chaudhury MK, Good RJ. Estimation of the polar parameters of the surface tension of liquids by contact angle measurements on gels. J Colloid Interf Sci. 1989;128(2):313–9. doi: 10.1016/0021-9797(89)90345-7

[106] Van Oss CJ. The Apolar and Polar Properties of Liquid Water and Other Condensed-Phase Materials. In: Interface Science and Technology [Internet]. Elsevier; 2008 [cited 2026 Jul 29]. p. 13–30. Available from: https://linkinghub.elsevier.com/retrieve/pii/S1573428508002020

