# Supplementary material for: Biophysical and functional characterization of the Escherichia coli Dr fimbrial envelope reveals its importance for pathogenesis and lithogenesis
Source: PLoS Pathog. 2026 Sep 16;22(9):e1014588. doi: 10.1371/journal.ppat.1014588 (PMC13608002; doi:10.1371/journal.ppat.1014588)

Original uncropped and unadjusted blot and gel images supporting the manuscript

Biophysical and functional characterization of the Escherichia coli Dr fimbrial envelope reveals its importance for pathogenesis and lithogenesis

Original uncropped and unadjusted Western blot corresponding to Figure 1E.

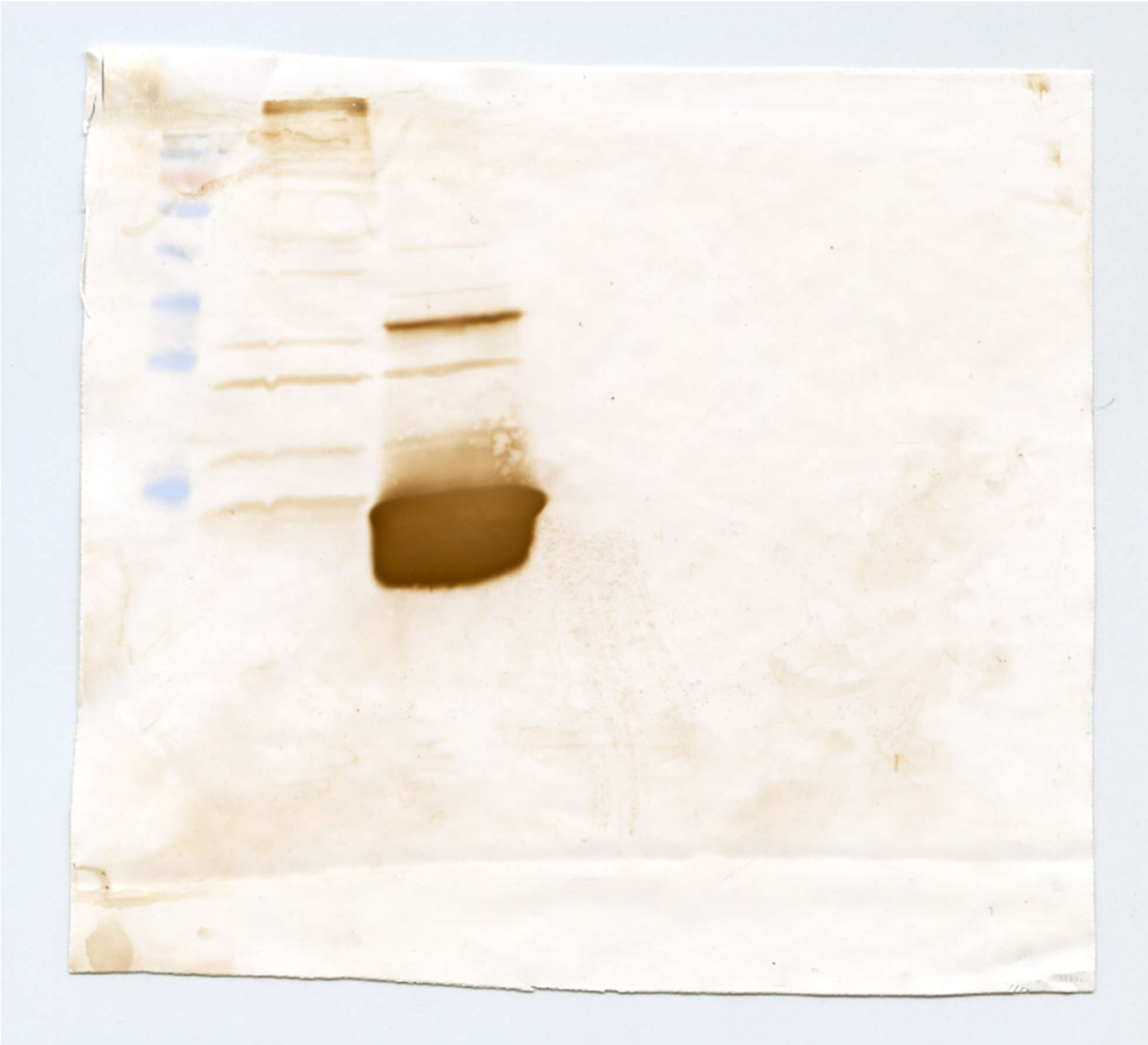

Original uncropped and unadjusted Western blot corresponding to Supporting Figure S1A.

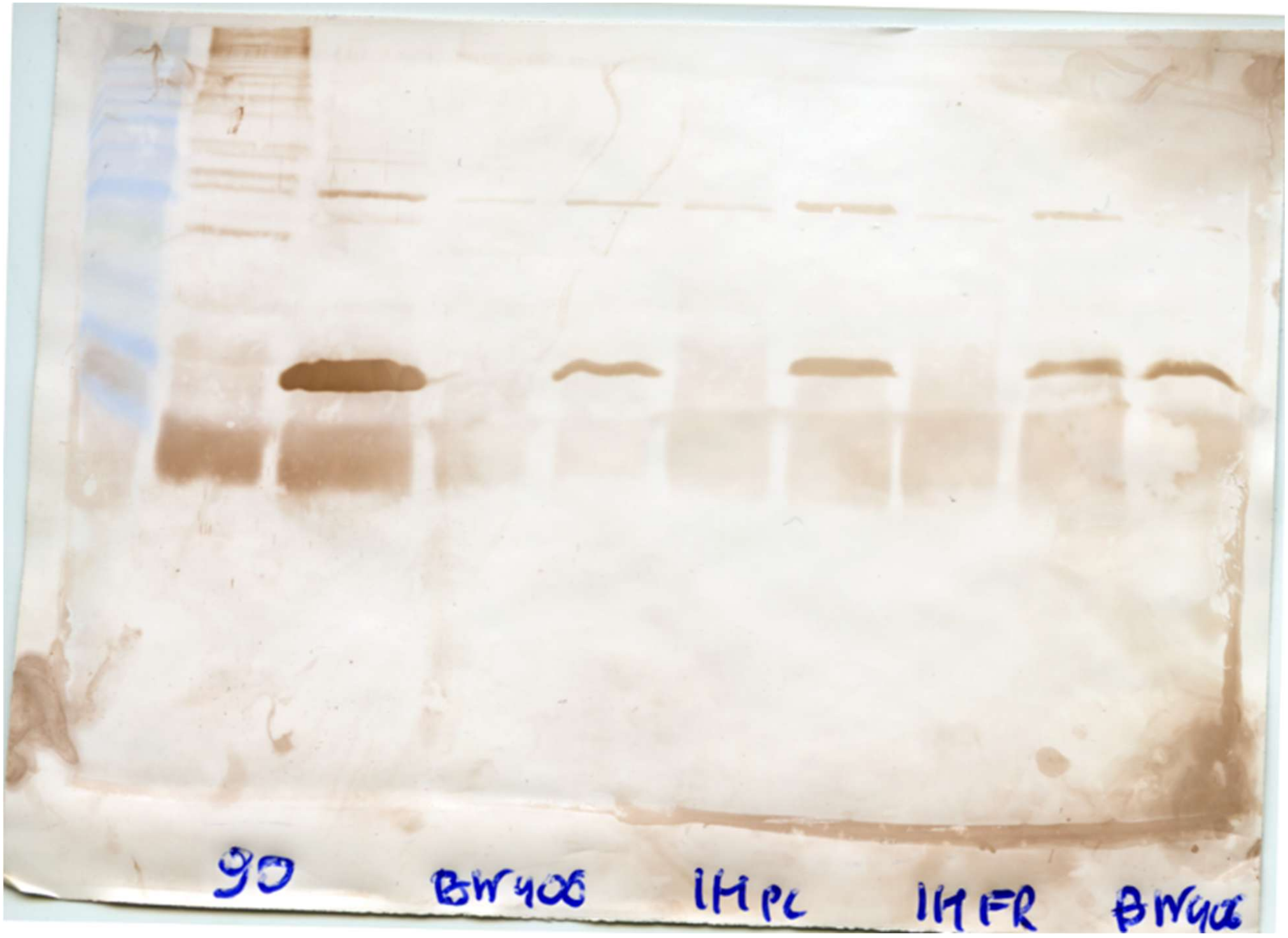

Original uncropped and unadjusted gel corresponding to Supporting Figure S5A.

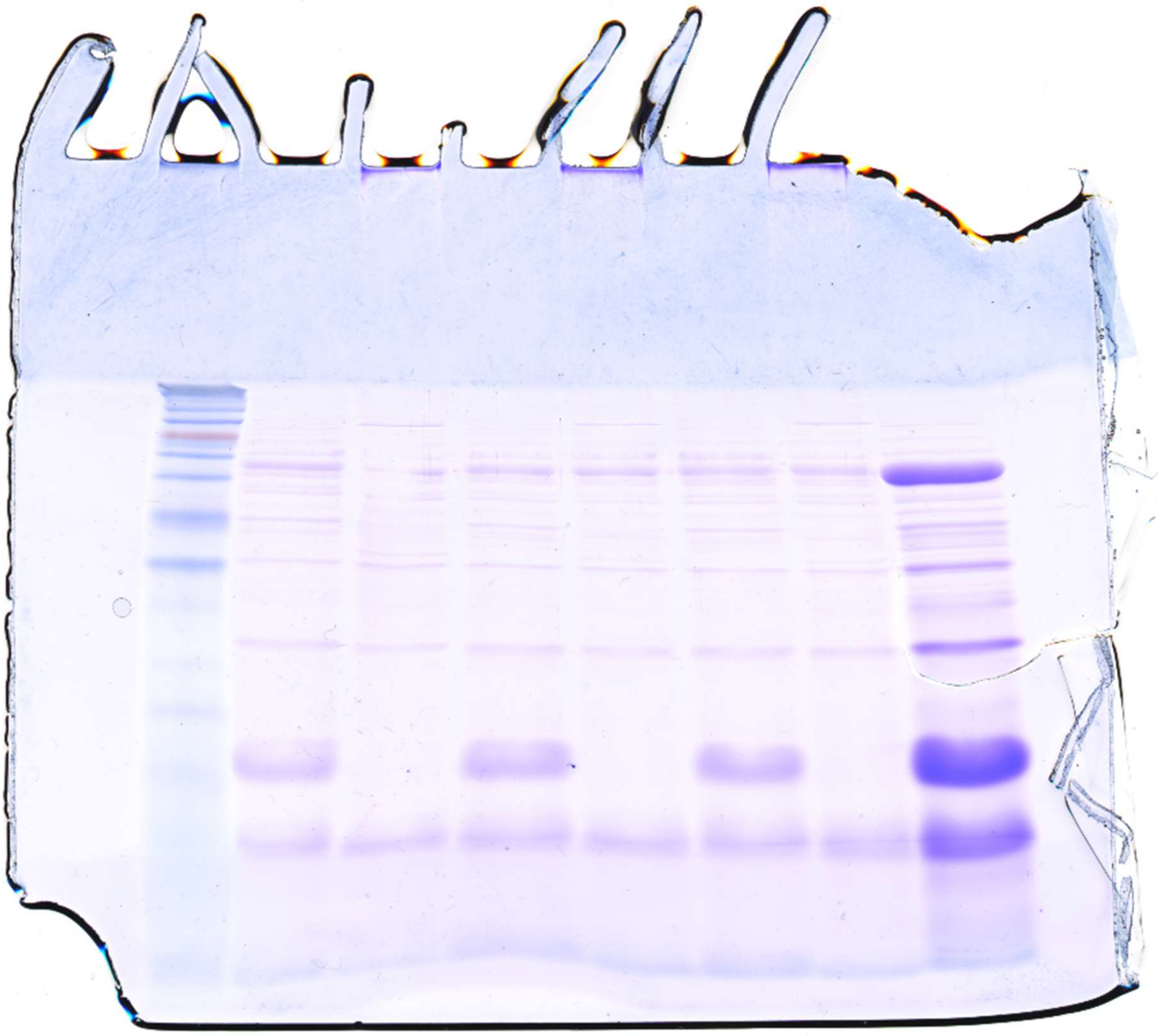

Original uncropped and unadjusted gel corresponding to Supporting Figure S5B.

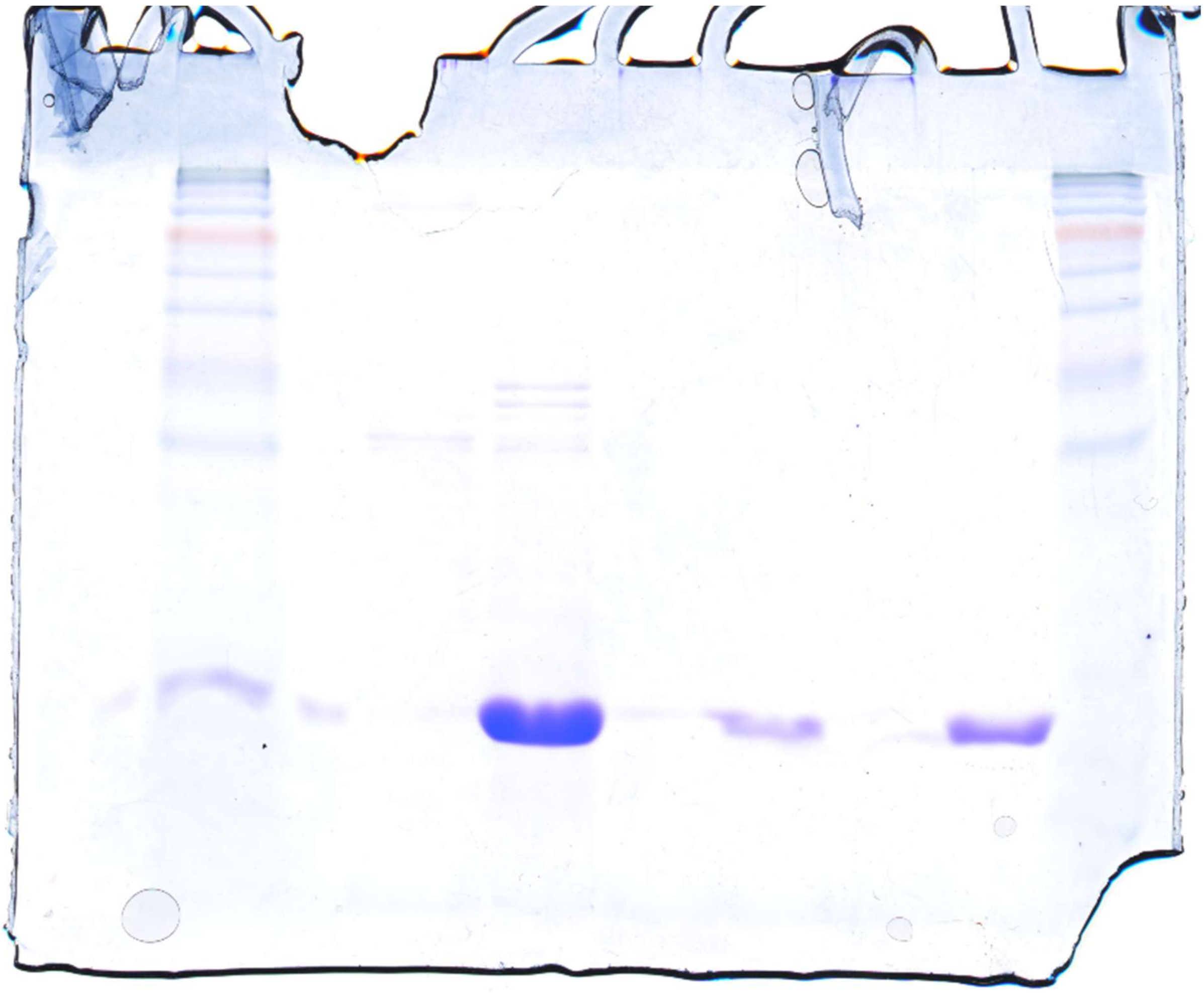

Supplement: Supplementary file 1 [file ppat.1014588.s001.pdf]
