# Supplementary material for: Biophysical and functional characterization of the Escherichia coli Dr fimbrial envelope reveals its importance for pathogenesis and lithogenesis
Source: PLoS Pathog. 2026 Sep 16;22(9):e1014588. doi: 10.1371/journal.ppat.1014588 (PMC13608002; doi:10.1371/journal.ppat.1014588)
Supplement: Supplementary file 2 [file ppat.1014588.s002.docx]

**Physicochemical Model of Bacterial Transport and Adhesion in a Water Film**

**1. Simplifying assumptions and their potential impact on the results**

To formulate an analytical model of cell anchoring within a moving meniscus, the following assumptions were made:

- **Ideal sphere geometry:** Cells are treated as spheres with a radius of $R=0.5 \mu m$. Neglecting the actual (often rod-like) shape may slightly affect the drag force distribution and contact area; however, at the capillary scale for small objects, this approximation is sufficient.
- **PBS buffer and DLVO theory:** The high ionic strength of the PBS solution (approx. 150 mM) drastically reduces the thickness of the electrical double layer (Debye length $\kappa^{-1}<1 nm$). This allows for the complete omission of electrostatic repulsion forces between the negatively charged cell and the glass surface.
- **Lack of non-specific resistance (**$\boldsymbol{F}_{\boldsymbol{T}}\boldsymbol{\approx}\boldsymbol{0}$**):** The experimental observation indicating 0% anchoring of non-fimbriated cells on glass allows us to assume that non-specific friction and resistance resulting solely from being pressed against a smooth substrate are insufficient to balance the meniscus shear forces.
- **Force distribution within the population:** It was assumed that the fimbrial anchoring force follows a normal distribution, and its standard deviation is 30% of the mean value ($\sigma_{adh}=0.3\mu_{adh}$).

**2. Identification and elimination of forces in the system**

A cell located at the liquid-solid-gas interface is potentially subjected to hydrodynamic and capillary forces (as well as buoyancy and friction, which can be practically neglected).

**2.1. Hydrodynamic drag force (**$\boldsymbol{F}_{\boldsymbol{D}}$**)**

For a laminar flow with a meniscus advancing velocity of $\upsilon=3.93 \mu m/s$ (calculated from $236 \mu m/60s$), the modified Stokes' law for a particle near a flat surface is expressed by the formula:

$F_{D}=6\pi\eta R\upsilon\cdot K\approx0.06 pN$	(1)

where $K=1.7$ is the wall correction factor, and $\eta\approx{10}^{-3}Pa\cdot s$ is the dynamic viscosity of water. This force is orders of magnitude smaller than the surface forces and is completely neglected in further considerations.

**2.2. Capillary force (**$\boldsymbol{F}_{\boldsymbol{C}}$**)**

This is the main driving force in the system, arising at the phase boundary (Figure 1). For water at ${25}^{o}C$ the surface tension is $\gamma\approx72 mN/m$. The maximum value of the capillary force is:

$F_{C}=2\pi R\gamma\approx226nN$	(2)

This vector is decomposed based on the contact angle $\theta={25}^{o}$:

- Shearing (scraping) component: $F_{C,x}=F_{C}\sin\left( {25}^{o} \right)\approx95.5 nN$	(3)
- Pressing (normal) component: $F_{C,y}=F_{C}\cos\left( {25}^{o} \right)\approx204.8 nN$	(4)

**Figure 1. Force distribution acting on a model bacterial cell at the receding liquid-solid-gas interface.** The diagrams illustrate the total capillary force ($F_{C}$) and its decomposition into shearing ($F_{C,x}$) and pressing/normal ($F_{C,y}$) components, which strongly depend on the contact angle ($\theta$). Left panel: Interaction with a glass substrate ($\theta={25}^{o}$), generating a significant downward pressing force ($F_{C,y}$). Right panel: Interaction with a polystyrene substrate, characterized by a different contact angle and force distribution. In order to resist the scraping meniscus, the sum of the specific fimbrial anchoring force ($F_{bond}$) and non-specific friction ($F_{T}$) must balance the shearing component.

**2.3. The role of Laplace pressure and local meniscus geometry**

In addition to the load exerted along the three-phase contact line, a cell trapped within a drying meniscus is subjected to capillary pressure. The concave profile of the meniscus formed in the narrow gap between two different slides generates a pressure difference described by the Young-Laplace equation (Eq. 5). This results in a force derived from the Laplace pressure acting on the cell's cross-section, which pulls the cell back into the liquid, effectively acting as an opposing force to the fimbrial adhesion. For the materials used in our experimental setup (glass and polystyrene), this opposing force acting on a cell with a radius of 0.5 µm was estimated to range from 5 to 13 nN. This force serves as a theoretical lower bound for the detachment force.

$\Delta p\approx\gamma\frac{cos{(\theta}_{top})+cos{(\theta}_{bottom})}{h}$	(5)

The exact vector and final magnitude of this force are closely tied to the local, microscopic shape of the meniscus, which deforms significantly as it bypasses the anchored cell. Because precisely defining this instantaneous geometry in a dynamic three-phase system is highly challenging, our conservative model integrates these coupled phenomena. By assuming an extreme scenario based on the macroscopic contact angle and ideal geometry, we established an absolute upper bound (approx. 110 nN) for the resultant surface load, combining the effects of both the contact line tension and the pressure difference. This represents the maximum mechanical resistance that the Dr fimbriae must overcome.

**3. Determination of the mean fimbrial anchoring force**

For a cell to resist the scraping process by the meniscus, the fimbrial bond force ($F_{bond}$) and the non-specific resistance ($F_{T}$) must balance the shear force:

$F_{bond}+F_{T}>F_{C,x}$	(6)

In accordance with the observed lack of anchoring for non-fimbriated cells, we assume $F_{T}\approx0$. Additionally, we can assume that the cell does not deform significantly under the influence of the capillary force. Thus, the survival condition reduces to:

$F_{bond}>95.5 nN$	(7)

Experimental data show that 67% of the population survived this test. This means that the detachment threshold cuts off the weakest 33% of the cells. In a standard normal distribution, the 33rd percentile corresponds to the parameter value $Z\approx-0.44$. The equation determining the mean anchoring force in the population ($\mu_{adh}$) takes the form:

$95.5=\mu_{adh}+Z\cdot\sigma_{adh}$	(8)

Substituting the assumption $\sigma_{adh}=0.3\mu_{adh}$:

$\mu_{\mathrm{adh}}=\frac{95.5}{0.868}\approx110 nN$	(9)

**4. Conclusion**

The calculated value of approximately 110 nN, derived from ideal geometry, represents the absolute theoretical upper limit of the system's total mechanical resistance. According to equation (6), this total encompasses both the specific fimbrial anchoring ($F_{bond}$) and non-specific friction ($F_{T}$). Although $F_{T}\approx0$ was initially assumed to establish this theoretical maximum, literature data and experimental observations demonstrate that biological systems always exhibit a certain level of non-specific adhesion (in the range of several nanonewtons).

By subtracting this baseline friction, and considering that the actual forces related to the meniscus may be mitigated by its local deformation on the cell, the actual specific fimbrial anchoring force ($F_{bond}$) is strictly lower than 110 nN. Furthermore, when factoring in the opposing Laplace pressure, the actual resistance forces can be estimated to fall between this theoretical lower bound and the upper limit of 110 nN, amounting to several tens of nanonewtons. This definitive estimation proves that the ability of Dr+ strains to block the moving contact line stems from generating a massive anchoring force, unprecedented for non-fimbriated cells, on the order of several tens of nanonewtons.
