# Supplementary material for: Biophysical and functional characterization of the Escherichia coli Dr fimbrial envelope reveals its importance for pathogenesis and lithogenesis
Source: PLoS Pathog. 2026 Sep 16;22(9):e1014588. doi: 10.1371/journal.ppat.1014588 (PMC13608002; doi:10.1371/journal.ppat.1014588)
Supplement: Supplementary file 3 [file ppat.1014588.s003.docx]

**S1 Table. Cell surface characters of the Dr fimbria-producing and non-producing AAEC191A bacteria.**

| **Contact angles and surface energy components** | | | | | | | | |
| --- | --- | --- | --- | --- | --- | --- | --- | --- |
| ***E. coli* strain** | **Contact angle [°]^a^** | | | **Surface tension components and parameters [ mJ m^-2^]^b^** | | | | **ΔG_BWB_^c^**  **[mJ m^-2^]** |
|  | **θ_w_** | **θ_F_** | **θ_D_** | **γ^AB^** | **γ^-^** | **γ^+^** | **γ^LW^** |  |
| **AAEC191A**  **Dr-** | 31.2±1.99 | 31.3±0.25 | 39.8±0.39 | 8.1±0.5 | 0.34±0.06 | 48.5±2.5 | 39.7±0.21 | 28.9±2.4 |
| **AAEC191A**  **Dr+** | 28.0±1.44 | 18.5±0.24 | 30.3±0.38 | 9.9±0.26 | 0.55±0.04 | 44.8±1.3 | 44.1±0.16 | 20.5±1.2 |
| **p-value^d^** | 0.087 | 3x10^-7^ | 6.83x10^-6^ | 0.005 | 0.009 | 0.08 | 6.35x10^-6^ | 0.005 |

a – the contact angle of water (θ_w_)_,_ formamide (θ_F_) and diiodomethane (θ_D_).

b – the Lifshitz-van der Waals (γ^LW^) and acid-base (γ^AB^) components of the total surface tensions and electron-donor (γ^-^) and electron acceptor (γ^+^) parameters.

c – the free energy (ΔG_BWB_) of interaction between bacteria when immersed in water.

d – P-values obtained in the unpaired Student’s t test.

**S2 Table. Bacteriophage efficiency of plating.**

| **Bacteriophage** | **Strain** | **Dr+/Dr- EOP** | **p-value** |
| --- | --- | --- | --- |
| P1 vir | AAEC191A | 0.116 ± 0.05 | 0.00207 |
| λ vir | AAEC191A | 0.10 ± 0.008 | < 2.2x10^-16^ |
| T7 | AAEC191A | 1.04 ± 0.05 | 1 |
| P1 | JM101 | 0.02 ± 0.01 | 1.596x10^-10^ |
| λ vir | JM101 | 0.23 ± 0.05 | < 2.2x10^-16^ |
| T7 | JM101 | 1.1 ± 0.24 | 1 |
| M13 | JM101 | 1.03 ± 0.06 | 1 |

EOP expressed as average ratio of three independent repeats ± standard deviation. P-values obtained in tests of proportions.

**S3 Table. Bacteriophage adhesion to *E. coli* AAEC191A Dr⁺ and Dr⁻ strains.**

| **Bacteriophage** | **Strain variant** | **Unabsorbed phages %** | **p-value** |
| --- | --- | --- | --- |
| P1 vir | Dr- | 2.2 ± 0.3 | 0.000065 |
| P1 vir | Dr+ | 79.5 ± 9.2 |  |
| λ vir | Dr- | 26 ± 1.4 | 0.0005 |
| λ vir | Dr+ | 69 ± 8.5 |  |
| T7 | Dr- | 29.1 ± 4.0 | 0.4721 |
| T7 | Dr+ | 28.9 ± 2.4 |  |

Adhesion expressed as average percentage of unabsorbed phages from three independent repeats ± standard deviation. P-values obtained in unpaired Student’s t test.

**S4 Table. Summary of [M+2H]²⁺ precursor masses for synthetic peptides used in targeted analysis of the Dra protein family.** The listed doubly charged m/z values correspond to peptide precursors selected for the generation of calibration curves. These values were used to extract chromatographic peak areas from the EICs (Extracted Ion Chromatograms) and quantify signal intensity. Subsequently, the standard curves derived from these peak areas enabled the determination of absolute protein concentrations in experimental samples. The retention time is also reported.

| **Protein** | **Precursor** | **m/z [M+2H]2+** | **Retention Time [min]** |
| --- | --- | --- | --- |
| **DraB** | SPAPFVVTPPLFR | 714.4059 | 27.92 |
| **DraC** | IEEYPALFR | 569.3006 | 24.34 |
| **DraD** | TGGDGWSPVK | 502.2458 | 19.34 |
| **DraE** | FFLISDNNR | 563.2881 | 24.27 |

|  | **Peptide [Area]** | | | |
| --- | --- | --- | --- | --- |
| **Concentration**  **[fmol]** | **DraE-FFLISDNNR** | **DraD-TGGDGWSPVK** | **DraC-IEEYPALFR** | **DraB-SPAPFVVTPPLFR** |
| 50 | 3728000 | 3830000 | 4470000 | 3100000 |
| 50 | 3824000 | 3850000 | 4460000 | 2990000 |
| 50 | 3921000 | 3940000 | 4280000 | 2860000 |
| 5 | 459600 | 309000 | 517000 | 318000 |
| 5 | 407000 | 302000 | 522000 | 341000 |
| 5 | 389800 | 321000 | 435000 | 359000 |
| 100 | 10300000 | 7870000 | 11500000 | 6740000 |
| 100 | 9762000 | 8210000 | 10300000 | 6300000 |
| 100 | 9935000 | 8930000 | 10400000 | 6200000 |
| 10 | 443500 | 355000 | 454000 | 353000 |
| 10 | 409600 | 433000 | 448000 | 383000 |
| 10 | 369900 | 421000 | 430000 | 368000 |
| 1 | 24920 | 9400 | 49800 | 9990 |
| 1 | 23960 | 9820 | 27000 | 11300 |
| 1 | 34230 | 9170 | 87400 | 11100 |

**S5 Table. Calibration curves generated for synthetic peptides corresponding to tryptic fragments of DraB, DraC, DraD, and DraE proteins.** Targeted proteomic analysis was performed using the ZenoTOF 7600 high-resolution mass spectrometer. For each protein, a representative tryptic peptide was selected based on uniqueness, ionizability, and chromatographic behavior. The following synthetic peptides were used: VAVVDVPDYR from DraB (precursor m/z [M+2H]²⁺ = 564.8021), SLDGLINFR from DraC (m/z = 515.2987), ITPEQDYVR from DraD (m/z = 567.3069), and GLLDVLGTVR from DraE (m/z = 534.8036). Extracted ion chromatograms (EICs) were generated for each precursor ion using a mass window of ±0.01 Da (i.e., total extraction width of 0.02 Da). Peptides were analyzed in triplicate across a dilution series, and integrated peak areas were plotted against known concentrations to construct linear calibration curves

**S6 Table. Quantitative LC-MS analysis of two tryptic peptides from the phage λ receptor protein LamB in the AAEC191A Dr⁻ and AAEC191A Dr⁺.** Peak areas for each peptide were extracted at the indicated retention times (RT) and charge states. Data represent raw integrated areas (CPSS) from three biological replicates per condition.

|  | **Sequence** | **NLIEWLPGSTIWAGK** | **ITLAQQWQAGDSIWSRPAIR** |
| --- | --- | --- | --- |
|  | **RT [min]** | 23.27 | 26.91 |
|  | **m/z (+2)** | 562.3067 | 766.4098 |
| **Area [cpss]** | **AAEC191A Dr-** | 7845171.22 | 1126624.64 |
|  |  | 7678593.80 | 962382.792 |
|  |  | 7910688.94 | 853882.543 |
|  | **AAEC191A Dr+** | 4961547.58 | 1015779.56 |
|  |  | 8451784.42 | 956558.517 |
|  |  | 6546553.35 | 1231913.15 |
