# Supplementary material for: Biophysical and functional characterization of the Escherichia coli Dr fimbrial envelope reveals its importance for pathogenesis and lithogenesis
Source: PLoS Pathog. 2026 Sep 16;22(9):e1014588. doi: 10.1371/journal.ppat.1014588 (PMC13608002; doi:10.1371/journal.ppat.1014588)
Supplement: Supplementary file 4 [file ppat.1014588.s027.docx]

**Response to reviewers**

**Reviewer #1**

**1. Nuccio and Bäumler (Microbiol. Mol. Biol. Rev., 2007) and Zavialov et al. (FEMS Microbiol. Rev., 2007) proposed an excellent classification of CUP fimbriae based on their phylogeny and functional properties. Dr fimbriae belong to the γ-family of classical CUP fimbriae and are closely related to the well-studied Yersinia F1 capsular (Caf), Ph6 (Psa), and Myf antigens. The Yersinia F1 capsular antigen functions as an "invisibility cloak," shielding other antigens and thereby protecting Y. pestis from phagocytosis. Thus, the concept of a "biophysical cloak" proposed by the authors is not entirely novel and should be discussed in the context of the published literature. In addition, the ultrastructure, physical properties, and binding characteristics of the Yersinia Caf, Psa, and Myf "polyadhesins" have already been thoroughly investigated and should also be discussed in the introduction.**

We sincerely thank the Reviewer for this valuable suggestion. In response, we have expanded the Introduction section to include references to F1, Ph6, Myf, CS6, and AAF/I–III structures. This is particularly important because, similarly to Dr fimbriae, these adhesins belong to the FGL chaperone–usher family and share closely related structural features. We also added a complete set of key literature references in the Introduction covering the most relevant studies describing the structure of these adhesins. In addition, in the Discussion section we now specifically refer to the F1 adhesin of *Yersinia pestis*, due to its reported role as a mechanical barrier limiting macrophage access to the bacterial cell surface. These additions strengthened the manuscript by highlighting that the observations made for the Dr envelope may also be relevant to other homologous FGL-type structures.

Finally, following the Reviewer’s suggestion and to avoid potential overinterpretation, we have discontinued the use of the terms “capsule” and “cloak” in reference to Dr fimbriae, and instead consistently use the terms “Dr fimbriae” and “Dr envelope” throughout the revised manuscript.

2. **Based on LC-MS analysis of DraE protein content in the Dr "capsule", the authors constructed a model of Dr fimbriae on the bacterial surface, estimating pilus density and "capsule" width. This is, in my opinion, a very interesting approach. However, the LC-MS analysis was performed using an artificial recombinant expression system, which may differ substantially from the expression levels observed in native Dr⁺ strains. Could the authors analyze a native strain using the same approach to validate their model? In addition, would it be possible to perform high-quality SEM analyses of Dr⁺ and Dr⁻ strains to further assess and support the proposed model?**

We thank the Reviewer for this important and very constructive comment. We fully agree that if Dr fimbriae production in our model strain *E. coli* AAEC191A Dr⁺ differed substantially from that of the native clinical strain IH11128, the relevance of our conclusions for UTI pathogenesis would remain only indirect. To address this point, we performed a detailed quantitative comparison of Dr fimbriae production between the model strain and the clinical isolate, and these new data are now included in the first section of the Results.

Dr fimbriae production in the clinical strain IH11128 is known to be strongly regulated, and even under optimized growth conditions only a fraction of the bacterial population expresses Dr fimbriae. Our immunofluorescence analysis demonstrated that under optimal conditions approximately 20% of IH11128 cells produce Dr fimbriae. Taking this proportion into account, we normalized densitometric measurements of fimbrial production in IH11128 and AAEC191A Dr⁺ cultures and obtained highly similar values when calculated on a per-cell basis. These results strongly support the conclusion that the recombinant model system reproduces physiologically relevant levels of Dr fimbrial production.

We also included TEM images of AAEC191A Dr⁺ cells in the Supplementary Information. These images were obtained only after numerous optimization attempts because Dr fimbriae are highly hydrophilic and extremely prone to mechanical disruption during sample preparation. In contrast, despite repeated efforts, we were unable to obtain equally satisfactory TEM images for IH11128. However, given the experimentally confirmed similarity in fimbrial abundance between both strains, we do not expect the surface organization in IH11128 to differ substantially.

At the same time, we would like to emphasize that performing most functional experiments directly on the clinical strain would not allow an unambiguous interpretation of the specific contribution of Dr fimbriae, because IH11128 carries multiple additional virulence-associated surface determinants whose influence on biological and physicochemical properties is difficult to isolate. In contrast, the well-defined AAEC191A model provides a controlled genetic background that allows direct attribution of observed biological and physicochemical effects specifically to the presence of Dr fimbriae.

We are grateful for this suggestion, as it prompted us to strengthen validation of the model system by directly relating Dr fimbrial production levels to those observed in the clinical strain.

3. **The LC-MS analysis of DraE protein content in the Dr capsule did not detect DraD. Could this reflect a limitation of the method, or is DraD indeed absent? Can the authors confirm this result using an alternative approach?**

We thank the Reviewer for this insightful question regarding the potential role of DraD in pathogenesis. Our LC-MS experiments were conducted under very stringent conditions to minimize quantitative error. In addition, we employed standard peptides for Dra proteins, which significantly enhanced the accuracy of protein quantification. The detection threshold for DraD in our measurements was approximately 34 molecules per cell.

It is important to note that the interpretation of DraD localization and function is complex and represents a topic worthy of a dedicated study. Consistent with the LC-MS data, DraD could not be detected in isolated fimbrial fractions using specific polyclonal anti-DraD sera. This observation may suggest that DraD is weakly associated at the tip of Dr fimbriae, as has been indirectly supported by previous structural studies (doi: 10.1107/S0907444905036747, Epub 2006 Jan 18; doi: 10.1186/1472-6807-11-25).

Moreover, DraD is highly sequence-conserved across the Dr adhesin family, is stably produced in the absence of chaperone proteins, and is exported independently of the usher protein. While the LC-MS result regarding DraD is certainly intriguing, the detailed discussion of DraD-related invasins is beyond the scope of the present study. We have therefore reported the observation as part of our LC-MS findings without further interpretation, focusing the manuscript on the aspects most directly relevant to Dr fimbriae function and UTI pathogenesis.

**4. The reported effect of Dr on colony morphology appears complex. What overall conclusion can be drawn from these experiments?**

We thank the Reviewer for this comment and agree with the observation. In the revised manuscript, we have chosen to focus primarily on three key aspects: the structural and physicochemical model of the Dr envelope, its role in mediating attachment to kidney stone components, and its function as a barrier against bacteriophages. In this context, the analysis of bacterial colony morphology contributes little to the main narrative and, for clarity and focus, has therefore been removed from the manuscript.

**5. Can the authors suggest a mechanistic explanation for why Dr expression slows swarming? Which cellular systems might be affected?**

We thank the Reviewer for raising this important point, which highlights the relevance of the Dr envelope model and its detailed biophysical characterization presented in our study. Swarming refers to the movement of bacteria within a thin liquid film, experimentally realized as a layer of water on a solid agar surface under controlled humidity conditions.

Two experiments in the manuscript provide a clear mechanistic explanation for the reduced motility observed in the AAEC191A Dr⁺ strain. First, bulk culture viscosity measurements demonstrated that the presence of Dr fimbriae strongly increases the viscosity of the system, which is expected to hinder swarming. Second, we introduced a novel experiment in which bacteria adhered to a glass surface were shown to pin the water meniscus, significantly slowing its motion. This phenomenon also contributes to reduced bacterial motility in thin liquid films.

We do not exclude the possibility that additional specific or nonspecific effects of Dr fimbriae on the bacterial surface may further influence swarming. Nevertheless, in the revised manuscript we have removed the swarming experiments, as they are not directly linked to the central themes of the study, which focus on the structural and physicochemical properties of the Dr envelope, its role in attachment to kidney stone components, and its protective effect against bacteriophages.

**6. The reported effects of fimbriae on aggregation, biofilm formation, viscosity, filtration, and sedimentation rates do not appear to be novel, as similar findings have been observed in other CUP systems. I recommend shortening these sections to allow more space for the novel findings (unless more detailed mechanistic data are added to these sections).**

We thank the Reviewer for this important comment and agree that individual effects of fimbriae on aggregation, biofilm formation, viscosity, filtration, or sedimentation have previously been reported for selected CUP systems. However, such studies remain relatively limited and highly fragmented, and to our knowledge there is still no comprehensive analysis integrating the physicochemical properties of a fimbrial surface structure into a unified functional model.

Following the Reviewer’s suggestion, we substantially reorganized this part of the manuscript. The biofilm-related section has been removed entirely. Autoaggregation and sedimentation data were reduced in scope and transferred to the Supplementary Information. At the same time, we significantly expanded the sections dedicated to experiments defining the physicochemical properties of the Dr envelope.

The structural model of the Dr envelope, obtained by integrating structural data, quantitative proteomics, TEM observations, and molecular dynamics simulations, enabled reconstruction of the three-dimensional organization of Dr fimbriae in direct relation to the entire bacterial cell surface. However, such a model alone remains essentially static and does not explain how the Dr envelope affects bacterial function. To assign biological, chemical, and physical meaning to this structure, we introduced a set of complementary and mechanistically linked experiments.

We first characterized fundamental surface parameters, including hydrophobicity/hydrophilicity and zeta potential. We then introduced a glutaraldehyde crosslinking assay, which provided insight into the degree of surface expansion of the Dr envelope and its interaction potential. This was followed by systematic analysis of bacterial interactions with different surfaces, including polystyrene, glass, calcium oxalate, and hydroxyapatite, examined under both static and dynamic conditions. We further added an adhesion-force-related experiment showing how surface-adhered bacteria influence water meniscus motion, providing an additional functional dimension to the model.

Taken together, these experiments transformed the structural reconstruction into a fully functional model of the Dr envelope. This model was then used to explain two biologically important phenomena directly linked to Dr fimbriae: protection against bacteriophages and selective binding to kidney stone components under artificial urine conditions. Importantly, we believe that the interpretative potential of this model extends beyond the phenomena described here and may serve as a framework for understanding additional biological properties of Dr-positive bacteria not addressed in the present study.

**7. The observation that Dr fimbriae may protect against phages is, by contrast, particularly interesting and novel. Has phage protection been reported for other CUP systems? Could the authors also examine whether capsule thickness influences the degree of protection?**

We thank the Reviewer for highlighting this aspect, as the bacteriophage experiments indeed emerged as a direct functional consequence of the structural Dr envelope model developed in this study. To the best of our knowledge, there are currently no published reports describing protection against bacteriophages mediated by a CUP-type adhesive envelope. The only analogous example we identified concerns the F1 (Caf) capsule of *Yersinia pestis*, which has been shown to act as a mechanical barrier limiting access of macrophages to the bacterial surface.

We agree that examining whether envelope thickness influences the degree of protection would be highly informative. However, in practice, establishing a promoter-controlled system allowing gradual modulation of Dr fimbriae production proved technically very difficult. The inducible systems we tested, including arabinose- and T7-based expression systems, consistently produced an essentially binary response (absence of production versus maximal production), regardless of inducer concentration. In our view, the most promising future approach for addressing this question would be to identify natural variants with altered Dr fimbrial expression levels, for example among UPEC ST131 isolates (doi:10.1128/mBio.03519-21).

Because of the potentially high biological significance of this phenomenon, we substantially expanded the bacteriophage experiments in the revised manuscript. First, we added bacteriophage T7 to the tested phage panel. Similar to P1, T7 recognizes LPS as its receptor, but its capsid is substantially smaller. Based on our Dr envelope model, we predicted that T7 should penetrate the Dr layer more readily and access the bacterial surface, which was confirmed experimentally. This allowed us to generate a well-calibrated size-dependent phage series (P1–λ–T7).

Second, we introduced a second bacterial strain, *E. coli* JM101, which differs markedly phenotypically from AAEC191A. This demonstrated that the observed protective effect is independent of strain genetic background.

Third, for AAEC191A we added direct measurements of phage adsorption to the bacterial surface. Equivalent adsorption measurements in JM101 did not yield interpretable results and were therefore not included.

Finally, we incorporated bacteriophage M13 as an additional control. Because M13 infects via sex pili, its infection cycle is independent of the bacterial outer surface architecture and therefore provides an important internal reference.

Altogether, the expanded dataset provides strong confirmation that the Dr envelope significantly restricts access of bacteriophages in the size range represented by P1 and λ, while allowing penetration of smaller phages such as T7.

**Reviewer #2:**

**Major concerns:
1. While the proposed structure interesting, it would be helpful to have some form of validation for the structure proposed (the authors indicate the potential size exclusion of the structure which could be tested, perhaps antibiotic exclusion). Since there are fibrils formed it would be helpful if there was an example of one or more individual fibrils in the proposed structure.**

We sincerely thank the Reviewer for this important comment, which addresses a key aspect of our study and directly concerns functional validation of the proposed Dr envelope model. We addressed this point experimentally at two complementary levels.

First, we expanded the bacteriophage panel by including bacteriophage T7. Similar to P1, T7 uses LPS as its receptor, but it is substantially smaller in size. This allowed us to generate a calibrated series of bacteriophages differing in capsid dimensions (P1–λ–T7), which enabled direct functional testing of the Dr envelope as a size-selective sieve. The experimental results were then confronted with the structural Dr envelope model and are now presented graphically in Fig. 11B. In addition, we strengthened this experiment by introducing a second *E. coli* strain (JM101) and bacteriophage M13 as an independent control.

Second, permeability of the Dr envelope to low-molecular-weight compounds is a fundamental issue both for bacterial physiology, including nutrient uptake, and for antibiotic susceptibility. We therefore tested experimentally whether the Dr envelope affects penetration of small molecules by comparing Dr⁺ and Dr⁻ bacteria against five different antibiotics. Two complementary experimental approaches were used: MIC determination and disk diffusion assays on solid medium. These results are presented in Fig. S14 and clearly demonstrate that the Dr envelope does not restrict diffusion of low-molecular-weight compounds to the bacterial cell surface.

Following the Reviewer’s suggestion, we also expanded Fig. 2 presenting the Dr envelope model in order to better visualize the contribution of individual fimbriae to the overall structure. This was implemented at two levels. First, we added a structural image showing several connected DraE subunits, which serves as the starting point for modeling the behavior of the entire envelope. Second, we included a selected fragment of the bacterial surface containing a small group of Dr fimbriae. This fragment is now shown both in top and side views, allowing better visualization of fimbrial packing density within the envelope. Importantly, this representation is provided for each tested sigma parameter value.

**2. The manuscript as presented is a series of experiments, the authors should guide the reader though their thought process of the study and when possible provide how the authors interpreted the data to proceed to the next set of experiments. the study would be strengthened with inclusion of rationale for each set of studies proposed and an overarching hypothesis. One example is why the authors would investigate viscosity of the culture.**

We fully agree with the Reviewer that the original version of the manuscript required a clearer logical structure and a more explicit presentation of the rationale linking successive experiments. In response, we made substantial efforts to reorganize the manuscript and strengthen the narrative flow.

First, we removed sections that were not directly connected to the central hypothesis of the study, including bacterial colony morphology, biofilm formation, swarming, and motility in liquid medium. Autoaggregation and sedimentation were reduced in scope and transferred to the Supplementary Information.

Second, throughout the revised manuscript we carefully restructured the Results section so that each experimental block directly follows from the conclusions of the preceding one. We introduced explicit transitions between sections, clarified the rationale for each experimental step, and added concise concluding statements indicating how each result guided the next stage of the study.

The revised manuscript now follows a clearly defined conceptual framework.

First, we validate the AAEC191A Dr⁺ model as a system faithfully reproducing Dr fimbriae production observed in the clinical strain IH11128. This step is essential because it establishes the clinical relevance of the model and allows subsequent conclusions to be interpreted in the context of UTI pathogenesis.

Second, we demonstrate through two simple macroscopic experiments, centrifugation and filtration, interpreted together with viscosity measurements, that the presence of Dr fimbriae produces measurable changes at the level of bacterial culture behavior. The rationale for investigating viscosity emerged directly from these observations, since both altered sedimentation and filtration suggested that Dr fimbriae substantially modify bulk physicochemical properties of the bacterial suspension. This section establishes that the Dr envelope indeed determines surface-related properties of bacterial cells and provides the starting point for ultrastructural and functional analysis.

Third, we construct the structural model of the Dr envelope by integrating TEM observations, quantitative proteomics, and computational modeling.

Fourth, we extend this structural model by incorporating chemical and physical properties, generating a fully functional three-dimensional representation of the Dr envelope supported by multiple physicochemical experiments.

Finally, we apply this functional model to explain two biologically important phenomena directly relevant to UTI pathogenesis: selective interaction of Dr-positive bacteria with kidney stone components and altered susceptibility to bacteriophage attack.

We believe that this revised organization now better reflects the underlying hypothesis of the study, namely that Dr fimbriae form a large-scale functional surface envelope whose structural properties directly determine bacterial physicochemical behavior and influence clinically relevant biological interactions.

**3. The rigor of the studies are unknown. In addition to restructuring the methods to be in the same order with the data presented, there should indication of the number of technical and biological replicates performed for each experiment.**

We agree with the Reviewer and appreciate this important comment regarding experimental rigor and clarity of presentation.

In the revised manuscript, the Methods section has been fully reorganized so that individual methodological descriptions now follow the same order as the corresponding experiments presented in the Results section. In addition, we introduced a dedicated *Statistical analysis* subsection that provides a general framework applicable to the majority of experiments.

In each section of the Methods, the number of biological replicates and the number of technical replicates per biological replicate are provided, along with the statistical test used for analysis. To improve readability, statistical information was also added directly to figure legends whenever possible, allowing the reader to interpret the data without the need to repeatedly cross-reference the Methods or Results sections.

To maintain readability, information on statistical significance was provided only where essential, rather than in every instance where it could be computed.

**4. The appropriateness of the conclusions would be better supported with less objective data than can be subjected to statistical analyses. Just a couple of examples:
a. The edges of the colonies in figure 3 are cropped and the observation of the overgrowth and undulating edges is difficult to see, the smoothness of the edges can be calculated on multiple colonies on multiple days and subjected to statistical analyses.
b. The swarming can also be measured to provide a range of mobility for each condition, again using multiple colonies on multiple days with statistical analyses
c. Biofilms do not grow uniformly on abiotic surfaces and the presentation of one single image per condition does not always provide a complete picture and it is always difficult to interpret the 3 D differences in a 2D image. Measurement of multiple random views of the biofilm height and inclusion of a 3D orthogonal view of the biofilms would also provide more interpretable data for the reader. The inclusion of videos is helpful, but they do not clearly portray the movement suggested by the authors. This is also the case for the examples of the detachment.**

We agree with the Reviewer and appreciate these detailed suggestions concerning quantitative support for visually presented data.

In the revised manuscript, the experiments related to colony morphology, swarming, and biofilm formation have been removed, as we concluded that they contributed only marginally to the central thematic focus of the study.

At the same time, the Reviewer’s recommendations were carefully taken into account for other experiments in which qualitative observations remain important. In cases where we present visually based phenomena, such as the polarized accumulation of Dr-positive bacteria on calcium oxalate crystals, movement of CaOx crystals mediated by attached Dr-positive bacteria, or microscopic analysis of bacterial interactions with calcium oxalate and hydroxyapatite under artificial urine conditions, we expanded the Supplementary Information to include additional representative images or videos obtained from at least three independent biological replicates.

This approach was intended to strengthen confidence in the reproducibility of the presented observations and to allow the reader to better appreciate the stability and consistency of the described phenomena across independent experiments.

Wherever possible, these qualitative observations are additionally supported by complementary quantitative measurements presented in the main manuscript

**5. PLoS Pathogens does not allow “data not shown”, the data must be provided.**

We agree with the Reviewer and fully complied with this requirement. The revised manuscript does not contain any statements referring to “data not shown.” All experimental results referred to in the text are now provided either in the main manuscript, in the Supplementary Information, or in the external data repository.

The manuscript currently contains 11 figures in the main text and, in the Supplementary Information, 15 figures, 5 video files, and 6 tables. In addition, extended datasets are deposited in the Zenodo repository.

**6. For the motility assays, please provide additional experimental details, how many frames per second were recorded, which software package was used, what function of the software was used, was the tracking of bacteria manual or automated?**

We thank the Reviewer for this comment. The bacterial motility experiment has been removed from the revised manuscript, as we concluded that it contributed only marginally to the central theme of the study.

At the same time, the Reviewer’s suggestions regarding video-based documentation were taken into account in other experimental sections where dynamic phenomena remain directly relevant. In these cases, supplementary video materials were expanded and accompanied by clearer methodological descriptions where necessary.

**7. What is the evidence that the Dr capsule is sensitive to mechanical damage? Please provide evidence or reference other published studies. Please also provide rationale for how a fragile structure could be a protective capsule.**

We thank the Reviewer for this important question.

Fimbrial envelopes represent very large supramolecular structures extending from the bacterial surface and, for this reason, are inherently susceptible to mechanical disruption under certain experimental conditions. A practical indication of this property is that one of the standard methods used for isolation of Dr fimbriae, as well as other CUP-type adhesins, relies on vigorous vortexing of bacterial suspensions to mechanically detach fimbrial structures from the cell surface (doi.org/10.1186/1471-2180-13-131; doi.org/10.1007/978-1-0716-0939-2_8). For this reason, during our experimental procedures even routine resuspension of bacterial pellets was performed very gently in order to minimize unintended fimbrial loss.

At the same time, the type of mechanical stress encountered under biological conditions differs fundamentally from that generated during in vitro handling. In vivo, bacteria are mainly exposed to laminar shear forces associated with fluid flow, whereas many laboratory manipulations may generate localized turbulent forces that are substantially more disruptive. Under physiological conditions such as those encountered in the urinary tract, fimbrial envelopes remain highly stable.

An additional factor is the strongly hydrophilic nature of the Dr envelope. Electron microscopy techniques such as TEM or SEM require extensive dehydration and often exposure to high vacuum, conditions that can induce major structural collapse or even complete destruction of highly hydrated surface assemblies. Similar effects have been reported for other hydrated biological structures, including bacterial mineral-associated surface matrices (doi:10.1073/pnas.2517066123).

Importantly, mechanical fragility under harsh experimental manipulation does not contradict the protective role of the Dr envelope under physiological conditions. Its protective function results from dense spatial organization and steric shielding at the bacterial surface, which remain effective under native hydrated conditions despite susceptibility to disruption during non-physiological sample processing.

**8. Please provide text for Figure 13.**

We thank the Reviewer for this comment. In the revised manuscript, we ensured that every figure, video, is explicitly cited in the main text at the appropriate location.

**Minor Issues:**

**1. The statement beginning on line 81 is not supported by the papers cited. Not all the papers report growth in human urine or the comparison with ASB strains. The relevance of this statement to the overall manuscript is unclear, especially in light of the numerous studies indicating that the primary site of UPEC replication in the bladder is within the superficial bladder epithelial cells.**

We thank the Reviewer for this important observation. We carefully re-examined the cited literature (references 3–5) and agree that the original text did not accurately reflect the content of these publications.

To address this, we have revised the manuscript to more accurately reflect these findings. The updated text now reads:

"UPEC strains are able to grow efficiently in the urinary tract environment during active human urinary tract infection (Forsyth et al., 3; Frick-Cheng et al., 4). Importantly, UPEC strains also display enhanced growth in human urine (HU) in vitro, achieving an approximately threefold shorter generation time and fourfold higher final optical density compared with reference non-uropathogenic E. coli strains (Phan et al., 5). These observations suggest that UPEC strains harbor specific adaptations that enable survival, growth, and persistence in an environment shaped by the chemical composition and hydrodynamics of urine in the bladder and kidneys."

**2. Line 169, consider changing the “Dr envelope” to “fimbriae” to be consistent with the remaining paragraph.**

We understand that using the term “envelope” in reference to Dr fimbriae may be somewhat confusing. However, throughout the manuscript we consistently use “Dr envelope” to emphasize that the Dr fimbriae form a continuous structure on the bacterial surface, effectively surrounding the cell and determining its surface properties. At the same time, we have discontinued the use of terms such as “capsule” or “cloak” in reference to Dr fimbriae, reserving those terms exclusively for polysaccharide-based capsules.
