# Supplementary material for: Biophysical and functional characterization of the Escherichia coli Dr fimbrial envelope reveals its importance for pathogenesis and lithogenesis
Source: PLoS Pathog. 2026 Sep 16;22(9):e1014588. doi: 10.1371/journal.ppat.1014588 (PMC13608002; doi:10.1371/journal.ppat.1014588)
Supplement: Supplementary file 5 [file ppat.1014588.s028.docx]

**Response to reviewers**

**Reviewer 1**

**Major Issues:**

**1. The experimental model used is not completely representative of a UPEC clinical isolate. The strain AAEC19A is a fim- derivative of E. coli K-12 strain MG1655 originally described by Ian Blomfield . What the authors need to state is that they are presenting a model for production of Dr+ and Dr- strains by using a K-12 derivative that lacks type 1 fimbriae. They present data that suggests that the Dr + “enveloppe” made using the plasmid system is seemingly similar to that made by the UPEC IH11128 strain based on biophysical comparison. This does not indicate that the K-12 strain is a model strain for the UPEC strain. There are a number of important differences in these strains that could affect many of the experiments presented in this paper. The UPEC strain can also make Type 1 fimbriae and also has a full O antigen (O75) that could affect some of the properties of the interactions concerning viscosity, aggregation, adherence to glass or interactions with kidney stone associated mineral salts.**

**Response:** We thank the Reviewer for this important comment and agree that the AAEC191A Dr⁺/Dr⁻ system should not be interpreted as a complete model of the UPEC strain IH11128. Our intention was to use the AAEC191A derivative, which lacks type 1 fimbriae, as a simplified and genetically well-defined background for investigating the physicochemical and functional consequences of Dr fimbriae production in isolation from other surface structures. To avoid any ambiguity, we have revised the manuscript throughout to consistently describe AAEC191A Dr⁺/Dr⁻ as a **model system for the Dr fimbrial envelope**, rather than as a model of the UPEC strain IH11128. This point is now also emphasized in the opening paragraph of the Discussion, where the scope and limitations of the model are explicitly addressed.

We fully agree that IH11128 differs from AAEC191A in several important aspects, including the presence of additional surface components such as type 1 fimbriae, the O75 lipopolysaccharide, and the K capsule. These structures may influence bacterial surface properties and therefore complicate direct quantitative comparisons between the two genetic backgrounds.

To address this limitation experimentally, we substantially expanded the manuscript by including a new section comparing the laboratory model with the clinical UPEC strain IH11128 and its Dr-negative derivative DR14. The comparison was intentionally based on several independent phenotypic assays. Importantly, the glutaraldehyde cross-linking assay alone does not provide direct evidence for the presence of a Dr fimbrial envelope. However, when interpreted together with the immunoaggregation assay using anti-Dr antibodies, it demonstrates that the cross-linked surface network observed in the clinical isolate is specifically associated with Dr fimbriae. Together with the filtration, MATH, meniscus adhesion, and calcium oxalate binding experiments, these results show that the principal Dr envelope-associated phenotype identified in the simplified laboratory model is also present in the clinical isolate.

Interestingly, the bacteriophage experiments further illustrate that the Dr envelope should not be considered in isolation from other surface structures. The clinical DR14 mutant, despite lacking Dr fimbriae, remained resistant to all three bacteriophages tested, whereas the laboratory AAEC191A Dr⁻ strain was susceptible. This observation strongly suggests that additional surface components of the clinical strain, most likely including the K capsule and the complete O antigen, constitute effective barriers against phage infection. At the same time, the experiments performed with the simplified AAEC191A model revealed the size-dependent filtering properties of the Dr fimbrial envelope itself. Taken together, these findings suggest that the Dr envelope and other surface structures of UPEC may act synergistically to restrict bacteriophage access to the cell surface. For example, the Dr fimbrial envelope could provide an additional protective barrier against phages recognizing capsular polysaccharides as receptors or producing capsule-degrading depolymerases. Although verification of this hypothesis will require studies using an appropriately selected collection of capsule-specific bacteriophages, we believe that our results provide a strong rationale for such future investigations.

We believe these revisions clarify both the scope of the AAEC191A model and the biological significance of validating the principal Dr envelope-associated phenotypes in the clinical UPEC strain IH11128.

**2. There are only a few experiments presented to compare levels of Dr adhesin made with the K-12 model compared to the UPEC strain. It is clear from the data that considerably more Dr adhesin is produced in the K-12 Dr+ strain than WT strain IH11128 based on the Western blot results in Fig. S1.**

**Response:** We thank the Reviewer for this important comment. We agree that the Western blot signal obtained from whole IH11128 cultures is substantially lower than that of the constitutively expressing AAEC191A Dr⁺ strain. However, this difference reflects the heterogeneous expression of Dr fimbriae in the clinical isolate rather than a lower level of fimbriae production by individual Dr-positive cells.

To address this issue, we performed an additional quantitative analysis. Western blotting of fimbrial fractions isolated from cultures normalized to identical OD₆₀₀ showed that the overall DraE signal in IH11128 corresponded to approximately 20 ± 5% of that observed in AAEC191A Dr⁺. Independently, immunofluorescence microscopy demonstrated that only 16 ± 4% of IH11128 cells expressed detectable Dr fimbriae, whereas all AAEC191A Dr⁺ cells were Dr-positive. After correcting the Western blot signal for the fraction of fimbriae-producing cells, the estimated amount of Dr fimbriae produced per Dr-positive IH11128 cell was found to be comparable to that of the AAEC191A Dr⁺ strain.

This comparison was introduced specifically to evaluate the physiological relevance of the constitutive model. It demonstrates that the AAEC191A Dr⁺ strain does not overproduce Dr fimbriae relative to the naturally expressing IH11128 subpopulation. Instead, it provides a homogeneous population of Dr-positive cells, enabling quantitative characterization of the Dr fimbrial envelope while maintaining a physiologically relevant level of fimbrial production per expressing bacterium.

**3. Why was IH11128 only used to demonstrate a comparative size of the Dr enveloppe from a WT clinical strain compared to the one that was used as a model being expressed from a medium (10 to 15) copy plasmid?**

**Response:** We thank the Reviewer for this comment. As explained in our responses to Comments 1 and 2, the purpose of including IH11128 was not to establish it as a second experimental model, but to validate that the Dr fimbrial envelope-associated phenotype identified using the genetically defined AAEC191A Dr⁺/Dr⁻ system is also present in a clinically relevant UPEC isolate.

Following the Reviewer's suggestions, we substantially expanded the manuscript by comparing the laboratory model with IH11128 and its Dr-negative derivative DR14 in multiple independent assays, including Dr fimbriae production, filtration behavior, MATH, glutaraldehyde cross-linking combined with immunoaggregation, meniscus-resistant adhesion, calcium oxalate binding, and bacteriophage susceptibility. Together, these experiments demonstrate that the principal Dr envelope-associated phenotypes identified in the model strain are also observed in the clinical isolate.

The AAEC191A Dr⁺/Dr⁻ pair nevertheless remains the primary experimental system because it provides a genetically defined background in which the presence or absence of Dr fimbriae is the only intentional variable, allowing the contribution of the Dr fimbrial envelope to be investigated independently of other UPEC surface structures. We have clarified this rationale in the revised manuscript.

**4**. **All of the data presented other than this demonstration of similar size for the enveloppe was done in a K-12 strain that was engineered not to make type 1 fimbriae. Nearly all E. coli typically make type 1 fimbriae. These fimbriae are also known to be important for UTIs particularly in the bladder. Although they may not be as produced during chronic infection in the kidney where Dr fimbriae may be more relevant, it would be more relevant to place the plasmid-based model into the clinical strain UPEC IH11128 and determine if it functions similarly in this strain background.

Effects of other surface products made by the UPEC strain would be of interest to consider for some of the phenotypes observed. Effects of possibly making type 1 fimbriae or effect of having a full LPS O antigen instead of the K-12 rough LPS importantly could potentially influence the phenotypes of envelope structure or thickness, potential association with kidney stone associated salts, or resistance to phage. It is unclear why the authors did not introduce this plasmid into the UPEC strain to test. It would genetically be cleaner to create a dra knockout mutant in IH11128 and then use the UPEC IH11128 pACYC177 as a Dr-negative and pCC90 as the Dr+ strain. It would need to be a different cloning vector possibly, if the clinical isolate is resistant to ampicillin.

Such an experimental design would more accurately represent features of a true UPEC strain, but with comparison of the added contribution that expression of the Dr fimbriae would have to the various phenotypes examined by the authors.**

**Response:** We thank the Reviewer for this valuable comment. We fully agree that the AAEC191A model lacks several important surface structures naturally present in the clinical UPEC strain IH11128, including type 1 fimbriae. More generally, it lacks all additional surface components that may be expressed by IH11128 under the experimental conditions used in this study. For this reason, we considered that the most appropriate way to evaluate the physiological relevance of the model was not to construct an artificially modified clinical strain, but rather to determine whether the key Dr envelope-associated phenotypes identified in the AAEC191A model are also observed in the native clinical strain IH11128 and its Dr-deficient derivative DR14.

Before these analyses, we confirmed by yeast agglutination that both IH11128 and DR14 produce type 1 fimbriae. We then compared the strains using a series of independent assays evaluating Dr envelope-associated phenotypes. Despite the presence of the full complement of naturally expressed surface structures in the clinical background, IH11128 exhibited the same characteristic Dr-dependent properties as observed in the AAEC191A model, including increased filtration time, enhanced affinity for xylene, stronger resistance to meniscus-induced detachment from glass, and markedly greater adhesion to calcium oxalate crystals than its Dr-deficient derivative DR14. Interestingly, the difference in calcium oxalate binding between IH11128 and DR14 was even more pronounced than that observed in the AAEC191A model.

In our opinion, these results provide stronger validation of the AAEC191A model than would be obtained by introducing a constitutively expressed plasmid-borne dra operon into the clinical strain. They demonstrate that the Dr envelope-associated phenotypes identified using the simplified model are also manifested in the native UPEC background despite the presence of numerous additional surface structures that may contribute to the overall surface properties of the bacterium under physiological conditions.

**Minor Issues:**

**1. I recommend changing the title to :Biophysical and functional characterization of the Escherichia coli Dr fimbrial envelope reveals its importance for pathogenesis and lithogenesis, as it is inaccurate or abiguous to indicate the envelope is “in” E. coli** .

**Response:** We thank the Reviewer for this suggestion. The title has been revised as recommended to avoid the ambiguity associated with the phrase "in *E. coli*" and to more accurately describe the Dr fimbrial envelope.

**2. Would Dr fimbrial expression actually be favoured under these conditions of acidic pH ?**

**Response:** We thank the Reviewer for this important question. In our study, neither the model strain AAEC191A Dr⁺ nor the clinical strain IH11128 was cultivated under acidic conditions. Both strains were routinely grown under standard conditions at approximately pH 7.2 (LB or M63). For most experiments, overnight cultures were subsequently resuspended in PBS adjusted to the desired pH (5.7, 7.0, or 8.0) or in artificial urine (AU, pH 5.7) immediately before the measurements. Therefore, the acidic pH used in these assays was intended to evaluate the physicochemical properties and functional behavior of preformed Dr fimbrial envelopes rather than to induce or enhance Dr fimbriae expression.

The only exception was the 24-hour artificial urine experiment examining interactions between AAEC191A Dr⁺/Dr⁻ bacteria and calcium oxalate crystals. However, even under these conditions, we did not observe any evidence that growth in AU at pH 5.7 altered the amount of Dr fimbriae produced per expressing cell compared with bacteria grown under standard conditions.

**3. Would production of some other type of CUP fimbriae , such as type 1 fimbriae not lead to the same type of interactions with mineralization compared to a negative control strain that is “completely bald” ?**

**Response:** We thank the Reviewer for this interesting question. We do not know whether other CUP-assembled fimbriae exhibit affinity for calcium oxalate crystals similar to that of Dr fimbriae, and this would require experimental verification.

However, our results obtained with the clinical strains strongly suggest that the interaction observed in this study is specifically associated with Dr fimbriae. The IH11128 clinical isolate and its isogenic Dr-deficient mutant DR14 were both confirmed to produce type 1 fimbriae, as demonstrated by their ability to agglutinate yeast cells. Despite retaining type 1 fimbriae and other native surface structures, DR14 almost completely lost its ability to bind calcium oxalate crystals, whereas IH11128 displayed strong and highly specific crystal adhesion, even though only approximately 20% of the bacterial population expressed Dr fimbriae due to phase variation.

**4. Within the kidney, during UTI, under what biological or environmental conditions would there likely be the presence of bacteriophage that could target E. coli, other than if they were to be used as phage therapy?**

**Response:** We thank the Reviewer for this important question. To our knowledge, there are currently no reports demonstrating the presence of bacteriophages within the kidney during urinary tract infection. Therefore, we cannot conclude that bacteriophage-mediated selection occurs in this specific environment.

However, bacteriophages are well documented in other regions of the urinary tract, including in the urinary microbiome of both healthy individuals and UTI patients, where they infect their bacterial hosts, including *E. coli* (doi:10.1128/JB.00738-17; doi:10.1038/s41585-019-0192-4). Furthermore, the vast majority of UTIs originate in the lower urinary tract, particularly the urethra and bladder, from which uropathogenic *E. coli* may subsequently ascend to the kidneys. Consequently, bacteria may encounter bacteriophages before reaching the upper urinary tract, and phage-mediated selective pressure cannot be excluded during this stage of infection.

Our bacteriophage experiments were therefore intended primarily as a functional assay to probe the permeability and size-exclusion properties of the Dr fimbrial envelope rather than to model a specific stage of kidney infection. They demonstrate that the envelope can act as a physical barrier to large viral particles while remaining permeable to smaller phages, thereby providing experimental validation of the structural predictions of our computational model.

**5. Line 67 : replace “their recurrent form “ with “recurrent infection “.**

**Response:** We thank the Reviewer for pointing out this wording issue. The text has been corrected as suggested by replacing **"their recurrent form"** with **"recurrent infection."**

**6. Line 68 : It is important to consider that only certain E. coli strains can make these Dr adhesins. It may be about 10-15% of UPEC strains in general with higher % associated with recurrent or chronic UTIs . Just stating it this way in a summary gives the impression these structural adhesins are always there… The text should indicate “certain E. coli strains or some UPEC “ and not “These bacteria” which the reader would falsely consider as something typically made by all strains. This is clearly not the case.**

**Response:** We thank the Reviewer for this important clarification. We agree that Dr fimbriae are produced only by a subset of uropathogenic *E. coli* strains and that the original wording was too general. The text has been revised accordingly by replacing **"These bacteria"** with **"Some UPEC strains"** to accurately reflect the restricted distribution of Dr adhesins among *E. coli* isolates.

**7. Lines 111-112- At this point, you should establish the use of “fimbriae” instead of “pili” throughout your text. Even if both terms are interchangeably used in different articles, it is important to only use one term throughout your text when referring to CUP fimbriae. Ideally at the first use of the term fimbriae in your introduction, at lines 111-112 you can write something like “Fimbriae (also called pili) are filamentous surface structures assembled via the chaperone-usher pathway in many Gram-negative bacteria (15, 16)**

**Response:** We thank the Reviewer for this valuable suggestion. We agree that using a single term throughout the manuscript improves clarity and consistency. Accordingly, we have adopted the terminology **"fimbriae"** (and **"fimbriated"**) consistently throughout the manuscript when referring to chaperone-usher pathway (CUP) adhesins, thereby avoiding possible confusion with other types of pili, such as sex pili. In the Introduction, we also clarified this terminology by introducing the statement that fimbriae (also called pili) are filamentous surface structures assembled via the chaperone-usher pathway.

**8. Line 120: The term “classical pili” has no strict meaning. The text should be referring to other CUP fimbriae known to be multisubunit filamentous structures such as type 1 or P fimbriae. Although the term pili and fimbriae can be used interchangeably, authors should make the comparison mainly with other types of reference CUP fimbriae known to be extended structures on bacterial surfaces. The term pili again can also refer to other things such as conjugative pili or type IV pili which are structurally distinct, so the wording “classical pili” which could also be interpreted to refer to such distinct bacterial surface structures should not be used**.

**Response:** We thank the Reviewer for this comment. We agree that the term “classical pili” is not sufficiently precise and may refer to structurally distinct bacterial surface appendages. To avoid ambiguity, we have replaced this term with “heteropolymeric fimbriae” in the revised manuscript, which more accurately describes the class of multisubunit filamentous CUP fimbriae, including well-characterized examples such as type 1 and P fimbriae.

**9. Lines 144-152: Would be best to still use fimbriae and related terms instead of pili for type 1 and P fimbriae throughout this paragraph, as these are also CUP assembly structures and as such in the text maintaining the term fimbriae for all of these CUP adhesins is recommended for uniformity.

If you are using the term “pili” for type 1 and P pili, then you should also call the Dr adhesins, “Dr pili” I n your manuscript. Ironically based on your results and other work the Dr ans Afa “afimbrial “ adhesins are structurally not polymeric filamentous fimbriae “nor pili “. However, if you routinely use “fimbrial enveloppe” or “fimbriae” to describe Dr adhesins instead of calling them “Dr pili” or a “pili enveloppe”, it would be best for consistency to also use the term fimbriae for type 1 and P fimbrial adhesins instead of “pili” .**

**Lines 156-174. The text includes use of pili as well as use of fimbriae to describe the same structures. For consistency, since you use fimbriae to describe Dr fimbriae, also use fimbriae to describe other classes of CUP structures such as P or type 1 fimbriae.**

**Response:** Please see our response to comment No. 7.

**10. Line 209. Where is it or why isn’t it shown what % of cells of Dr+ model produce Dr enveloppe?**

**Response:** We thank the Reviewer for this comment. We have added the information in the revised manuscript that 100% of AAEC191A Dr⁺ cells exhibit fluorescence, indicating that all cells in the Dr⁺ model strain produce the Dr fimbrial envelope.

**11. Line 597. Authors should specific that E. coli JM101 produces F pili and is normally susceptible to M13 phage.**

**Response:** We thank the Reviewer for this helpful suggestion. We have incorporated this clarification into the revised manuscript by explicitly stating that *E. coli* JM101 produces F pili and is naturally susceptible to M13 bacteriophage infection.

**12. Line 613. It is incorrect to write that AAEC191A “ is a model strain of UPEC IH11128”. By placing such words in commas after AAEC191A it is suggesting that this strain is a mutant or derivative of the UPEC IH11128. The strain AAEC19A is a fim- derivative of E. coli K-12 strain MG1655 originally described by Ian Blomfield . What the authors need to state is that they are presenting a model for production of Dr+ and Dr- strains by using a K-12 derivative that lacks type 1 fimbriae. They present data that suggests that the Dr + “enveloppe” made using the plasmid system is seemingly similar to that made by the UPEC IH11128 strain based on biophysical comparison. This does not indicate that the K-12 strain is a model strain for the UPEC strain. There are a number of important differences in these strains that could affect many of the experiments presented in this paper. The UPEC strain can also make Type 1 fimbriae and also has a full O antigen (O75) that could affect some of the properties of the interactions concerning viscosity, aggregation, adherence to glass or interactions with kidney stone associated mineral salts. As such, authors have a model based on a fimbriae-negative strain that can compare effects of production of Dr adhesin on various phenotypes that they have tested.
Using this simplified model allows the authors to investigate biophysical properties of a Dr enveloppe produced by a fim-negative E. coli K-12 strain.**

**Response:** We thank the Reviewer for this important clarification. We agree that AAEC191A should not be described as a model strain of the clinical UPEC isolate IH11128. As discussed in our responses to the previous comments, the purpose of the AAEC191A system is to provide a genetically well-defined K-12 background that enables direct comparison of Dr⁺ and Dr⁻ phenotypes while minimizing interference from other surface structures, including type 1 fimbriae.

Accordingly, we have revised the wording throughout the manuscript to avoid implying that AAEC191A is a model of the IH11128 strain. Instead, we explicitly describe it as a model for investigating the contribution of the Dr fimbrial envelope to bacterial surface properties. We also refer the Reviewer to our detailed responses to Comments 2–4, where we explain the rationale for this experimental system, its validation against the clinical strain IH11128, and the complementary experiments performed with IH11128 and its Dr-deficient mutant DR14 to confirm the physiological relevance of the observations made using the model strain.

**13. Line 632. Correct spelling of fimbriae**

**Response:** We thank the Reviewer for noticing this typographical error. The spelling has been corrected in the revised manuscript.

**14. Line 781. Although there may not have been reports of phage inhibition through formation of a physical barrier by fimbriae, type 1 fimbriae have been shown to prevent or resist killing by a type VI secretion system recently…

mBio. 2024 Mar 18;15(4):e02553-23. doi: 10.1128/mbio.02553-23. Dessartine et al. 2024
Type 6 secretion killing is similar in mechanism to bacteriophage-mediated killing.**

**Response:** We thank the Reviewer for bringing this relevant study to our attention. We have incorporated this information into the Discussion and added the suggested reference (Dessartine *et al.*, 2024).

**15. Line 849…. Why isn’t the strain IH11128 described in the bacterial strains section as a urinary tract pathogenic (UPEC) strain?**

**Response:** We thank the Reviewer for this helpful suggestion. We have expanded the **Bacterial strains and culture conditions** section to provide a more complete description of the IH11128 and DR14 strains, explicitly stating that IH11128 is a uropathogenic *Escherichia coli* (UPEC) clinical isolate and describing the origin and characteristics of both strains.

**Reviewer 2:**

**Major Issues:**

**1. The manuscript places strong emphasis on phenotypes that emerge (only?) at very high fimbrial densities (1600 per cell). The authors argue that this regime reflects a naturally occurring high-expressing subpopulation. However, the regulatory basis for achieving and maintaining such extreme expression levels remains unclear. In a phase-variable system, while rare high-expressing cells indeed may arise, the absence of a mechanism that actively sustains elevated expression would be expected to relatively fast result in regression toward a population mean over successive divisions. Consequently, extremely high surface densities are likely to represent transient states rather than maintained phenotypic subpopulations. While this does not say that such rare strongly fimbriated bacteria are not important, their potential roles in pathogenesis thus should be interpreted with this in mind. Not at least since the central conclusions of the manuscript, which is also reflected in the title, rely heavily on phenomena observed at these high densities, the authors should more firmly discuss the physiological relevance of this regime and include thoughts about either how high expression levels can be sustained through regulatory pathways or why this is not important for the suggested function in pathogenesis.**

**Response:** We thank the Reviewer for this thoughtful comment. We agree that Dr fimbriae expression in IH11128 is phase-variable and that individual cells may switch between the ON and OFF states. However, we believe that our experimental design minimizes the impact of this variability and allows the analysis of a physiologically relevant population-average phenotype rather than transient expression states of individual bacteria.

To ensure physiological Dr fimbriae expression, the clinical strain IH11128 was routinely cultivated in M63 minimal medium supplemented with 1% casamino acids and 0.2% glycerol. This medium supports considerably lower bacterial densities than LB medium (overnight cultures typically reached OD₆₀₀ ≈ 2.5 compared with OD₆₀₀ ≈ 4–5 in LB) and was intentionally selected to avoid conditions that might promote non-physiological fimbrial overproduction. Interestingly, although the proportion of Dr-positive cells depended on the growth medium (approximately 20% in M63 medium and approximately 10% in LB), the amount of Dr fimbriae produced by individual Dr-positive cells remained comparable. Moreover, supplementation of the medium with glucose shifted the population almost completely to the OFF state, further demonstrating the importance of physiological growth conditions. For these reasons, IH11128 was always cultivated under the same standardized conditions throughout this study, whereas the constitutively expressing AAEC191A model strain, which lacks phase variation, was routinely grown in LB medium.

Importantly, all experiments were performed using overnight cultures rather than exponentially growing bacteria. Overnight cultures provide highly reproducible, population-averaged samples in which the fraction of Dr-positive cells reaches a stable equilibrium under defined culture conditions. This approach minimizes fluctuations associated with transient switching events that are more pronounced during exponential growth.

To evaluate the physiological relevance of the AAEC191A model, we deliberately introduced, at the beginning of the Results section, a quantitative comparison of Dr fimbriae production between AAEC191A Dr⁺ and IH11128. Immunofluorescence microscopy was used to determine the fraction of IH11128 cells expressing Dr fimbriae, whereas Western blot analysis of isolated fimbrial fractions provided an estimate of the amount of assembled surface fimbriae rather than total DraE protein, which may also exist as unassembled subunits within the cell. Combining these independent measurements demonstrated that, after correction for the proportion of Dr-positive cells, the amount of Dr fimbriae produced per expressing bacterium is comparable between IH11128 and the constitutively expressing AAEC191A Dr⁺ strain.

Furthermore, the additional validation experiments performed in the revised manuscript demonstrated that the principal Dr envelope-associated phenotypes identified in the AAEC191A model are also observed in the clinical strain IH11128 relative to its Dr-deficient derivative DR14. As expected, the magnitude of these effects is reduced because only approximately 20% of IH11128 cells express Dr fimbriae, yet the same qualitative phenotypes remain clearly detectable.

Finally, we would like to emphasize that both the computational model of the Dr fimbrial envelope and all quantitative analyses presented in this study are population-average models. We do not claim that every bacterium produces an identical number of fimbriae or that expression heterogeneity does not exist. Rather, our goal was to characterize the average structural and physicochemical properties of the Dr fimbrial envelope under standardized physiological conditions. To make this point explicit, we have revised the manuscript to state that the computational model represents the average characteristics of the bacterial population analyzed.

We believe that this population-based approach, together with the use of standardized culture conditions and validation in the clinical isolate, provides a physiologically relevant framework for investigating the role of the Dr fimbrial envelope in UPEC pathogenesis.

**2. Om the same these, the manuscript would benefit from a more detailed and quantitative comparison between the experimental system and native expression levels. The antibody-based fluorescence analysis used to estimate fimbrial abundance is not described in sufficient detail to assess its robustness. Providing information on staining conditions (e.g., antibody concentrations, incubation times, exposure settings), as well as quantitative single-cell intensity distributions, would greatly strengthen this comparison. Demonstrating that the upper range of the native population overlaps with the overexpression regime would be especially important.
An additional suggestion is to make independent physical measurements that could provide valuable complementary validation. For example, if the high-density fimbrial envelope significantly increases the effective size of the bacterium, as suggested, this should be reflected in measurable changes in hydrodynamic radius or diffusivity. Comparing such measurements between native high-expressing cells and the overexpression system could help establish whether they indeed occupy a similar physical regime. Notably, such measurements would also provide an independent means to test key predictions of the computational model presented in the manuscript (see comment 3).**

**Response:** We thank the Reviewer for this valuable suggestion. In the revised manuscript, we substantially expanded the methodological description of all immunological procedures used to estimate Dr fimbriae production.

Specifically, we added a separate section describing the antisera and antibodies used throughout the study, including the preparation of the rabbit anti-Dr polyclonal serum, its ELISA-determined antibody titer, and the sources and catalog numbers of all secondary antibodies. The Western blotting methodology was expanded to include membrane blocking conditions as well as the concentrations and incubation conditions of both primary and secondary antibodies. Likewise, the immunofluorescence protocol used to determine the fraction of IH11128 cells expressing Dr fimbriae was described in detail, including antibody dilutions, incubation conditions, washing steps, and the quantitative procedure used to determine the percentage of Dr-positive cells.

Regarding fluorescence intensity distributions, we respectfully believe that such an analysis would not provide a robust quantitative estimate of fimbrial abundance for bacterial cells. In wide-field immunofluorescence microscopy, the measured fluorescence intensity depends on numerous experimental parameters, including illumination intensity, optical alignment, filter characteristics, sample preparation, and image acquisition settings. Consequently, exposure times are routinely adjusted to maximize signal while avoiding detector saturation and are therefore not directly comparable between experiments.

Importantly, during optimization of the immunofluorescence protocol, we did not observe evidence for distinct subpopulations exhibiting markedly different fluorescence intensities. Instead, fluorescence appeared progressively in all Dr-positive cells as exposure time increased. Furthermore, when independently stained AAEC191A Dr⁺ and IH11128 cultures were mixed and analyzed in the same microscopic field under identical acquisition conditions, we were unable to distinguish two populations based on fluorescence intensity. Rather, Dr-positive cells from both strains exhibited comparable fluorescence signals, consistent with our conclusion that the amount of surface-associated Dr fimbriae per expressing cell is similar in the constitutive model and the naturally expressing IH11128 subpopulation.

For these reasons, we believe that the combination of quantitative immunofluorescence to determine the fraction of Dr-positive cells together with Western blot analysis of isolated fimbrial fractions provides a more reliable estimate of relative fimbrial production than fluorescence intensity measurements of individual bacterial cells.

We fully agree that independent physicochemical measurements capable of distinguishing Dr-positive from Dr-negative bacteria would provide valuable complementary validation of the computational model.

In fact, this question has become one of the major directions of our current research. Distinguishing between homogeneous Dr⁺ and Dr⁻ bacterial populations using purely physicochemical methods has proven to be considerably more challenging than initially anticipated, and the analysis of heterogeneous populations, such as the clinical strain IH11128, represents an even greater challenge. We initially explored dynamic light scattering (DLS), but the results were not sufficiently robust for reliable quantitative comparison of bacterial populations.

These limitations motivated us to develop alternative physicochemical approaches that are not routinely used in microbiology. Most recently, we introduced a density-based method for determining the apparent volume of bacterial cells, which successfully discriminates between homogeneous Dr⁺ and Dr⁻ populations. The initial results have been published (Wawer et al., Determination of the apparent volume of bacteria using the example of E. coli strain producing Dr fimbriae, Journal of Molecular Liquids, 2025, <https://doi.org/10.1016/j.molliq.2025.128698>). Extension of this approach to heterogeneous populations, including IH11128, is currently in progress.

Although these studies are highly promising, they extend beyond the scope of the present manuscript. We therefore believe that the current validation of the computational model using multiple independent structural, physicochemical, and functional assays provides the most comprehensive assessment currently available.

3**. The computational framework presented to model the Dr fimbrial envelope is conceptually interesting and provides a useful interpretation of how high-density fimbrial organization may shape the bacterial interface. The observed size-dependent exclusion of larger phages, while allowing smaller phages and molecules to penetrate, is consistent with the existence of a porous fimbrial layer and lends qualitative support to the model. However, several aspects of the model would benefit from further clarification and quantitative grounding.
First, the introduction of the "effective solubility" parameter (σ) appears largely phenomenological. While it is motivated in terms of electrostatic repulsion and hydrophilic swelling, no clear physical mapping or independent constraints are provided for its value. As currently implemented, σ effectively acts as a tunable parameter controlling envelope thickness and density profiles. It would strengthen the manuscript if the authors could relate σ to measurable physical properties (e.g., charge density, ionic strength, filament stiffness) or otherwise motivate the selected range of σ theoretically or experimentally.
Second, the assumption of uniform fimbrial length (≈140–150 subunits per polymer) is likely an oversimplification. In reality, fimbrial assembly is expected to produce a distribution of lengths, which could significantly affect the effective envelope thickness and its radial density profile. In particular, length dispersity would be expected to smooth and potentially reduce the effective outer boundary of the envelope. This limitation should be discussed, and if feasible, incorporated into the modeling framework.
Finally, the model makes useful and testable predictions about the physical extent of the fimbrial layer (200–300 nm). Additional quantitative validation would further strengthen the conclusions. For example, measurements of effective hydrodynamic size or cell diffusivity under different expression levels could provide an independent estimate of envelope thickness and complement the permeability data.**

**First, the introduction of the "effective solubility" parameter (σ)…**

We agree with the reviewer that the largely heuristic nature of the parameter does not allow for a strict physical interpretation, instead relating to effective measures of solubility or electrostatic repulsion. To more strongly ground the explored values in data, we compared the top-projected densities of the fimbrial layer between our computational models and signal intensities observed in **electron microscopy with positive staining**. As can be seen in **Fig. S6,** the two profiles (selected from the least cluttered of the images available) closely match the value of σ = 0.67 or slightly larger, although we do not discard the possibility that different environmental conditions will strongly affect this value.

**Second, the assumption of uniform fimbrial length (≈140–150 subunits per polymer)…**
The uniform value is clearly an oversimplification, yet no available experimental or mechanistic data could provide a valid estimate of length variability. To explore this effect of length variability, we generated multiple and plotted their densities as a function of distance from the OM (**see Fig. S7**) and note that until very high standard deviations, where individual fimbriae exceed 200 units, the density profiles remain largely unchanged.

**Minor Issues:**

**1. The manuscript presents a substantial and interesting body of experimental work however the organization and writing style could be improved to enhance clarity and readability. Several sections describing experiments that address the functional impact of the Dr fimbrial envelope appear repetitive. Bringing related experiments together and supporting them with more integrated figures or graphical summaries would improve the overall flow and make the narrative easier to follow. This could also significantly shorten the manuscript, which is now longer than needed without adding proportional clarity. In addition, some Results subsections end with brief interpretative or concluding statements, while others do not. Given the clear separation between Results and Discussion, it would be more consistent to reserve interpretation and synthesis for the Discussion section.**

**Response:** We thank the Reviewer for this constructive suggestion. We agree that the manuscript has become relatively long, reflecting the large number of complementary experimental approaches required to characterize the Dr fimbrial envelope from structural, physicochemical, functional, and computational perspectives.

We carefully considered reorganizing and further integrating the Results section. However, we concluded that the current organization best preserves the logical progression of the study, in which each experiment provides the basis for the subsequent analyses. Because many of the figures are already multipanel and approach the maximum size accepted by the journal, further merging of figures would require substantial reduction in panel size, compromising readability and image quality.

Moreover, in response to the reviewers' comments, we substantially expanded the manuscript by adding a new section validating the AAEC191A model against the clinical UPEC strain IH11128 and its Dr-deficient derivative DR14. This required the inclusion of three additional multipanel figures (Figures 12–14) in the main manuscript and two new supplementary figures that further strengthen the computational model. We also substantially revised and expanded the Discussion to relate the findings obtained with the AAEC191A model to the clinical strain.

Given the breadth of the experimental work, we believe that presenting the Results as a series of clearly separated subsections improves readability and allows readers to follow the experimental rationale more easily than would a more condensed presentation. Nevertheless, we carefully revised the manuscript to improve clarity and remove unnecessary repetition wherever possible.

2. The authors describe the fimbrial layer using terminology such as “supramolecular assembly” and “brush-like” organization, which suggests an analogy to grafted polymer brushes. While this conceptual framing is intuitively appealing, it may not be physically well justified in the present system and would benefit from further clarification.
Classical polymer brush behavior arises in the regime of very high grafting density, where excluded volume interactions between neighboring chains generate strong entropic stretching, leading to a relatively well-defined, surface-like polymer boundary. In contrast, the estimated spacing between fimbriae in this work (on the order of tens of nanometers) suggests a comparatively low grafting density, where intermolecular interactions are expected to be weak and infrequent. In such a regime, one would expect a more diffuse, heterogeneous three-dimensional distribution of filaments rather than a sharply defined “brush” interface.
This distinction has important implications for how the fimbrial layer is interpreted. Rather than behaving as a classical brush with a well-defined height, the Dr fimbrial coating may be better described as a sparse, flexible fibrous network forming a volumetric layer with a gradually decaying density profile. Referring to the outer boundary of this layer as a “surface” may therefore be conceptually misleading, as no clear physical interface is expected to emerge in the absence of strong collective stretching or packing effects.
Moreover, if steric interactions between fimbriae are indeed limited, the organization of the layer is likely governed primarily by individual filament flexibility and stochastic spatial distribution, rather than cooperative entropic effects characteristic of dense polymer brushes. Clarifying this distinction would help avoid overinterpretation of the polymer brush analogy and better align the model with the expected physical regime of the system.

**Response:** We thank the Reviewer for this thoughtful and insightful comment. We agree that the term *polymer brush* may imply the classical Alexander–de Gennes polymer brush regime, in which a very high grafting density leads to strong steric interactions, collective entropic stretching of neighboring chains, and the formation of a well-defined polymer interface. This was not our intention.

Our computational model does not assume collective stretching or strong steric interactions between neighboring fimbriae. On the contrary, individual fimbriae are treated as flexible filaments whose spatial organization is governed primarily by their intrinsic flexibility and stochastic conformations. Consequently, the resulting Dr envelope is represented as a three-dimensional porous supramolecular network with a gradually decreasing density rather than a sharply defined polymer interface.

We therefore agree that describing the Dr envelope as a *brush-like* structure may overemphasize the analogy with classical polymer brushes. To avoid this potential misunderstanding, we have revised the manuscript by replacing this terminology with more accurate descriptions, such as *fibrous network*. In the Discussion we replaced the sentence:

*"Together, these observations indicate that the Dr capsule behaves as a dynamic hydrated polymer brush whose local organization can adapt to environmental conditions without losing its structural continuity."*

with:

*"Together, these observations indicate that the Dr capsule behaves as a dynamic hydrated fibrous network whose local organization can adapt to environmental conditions without losing its structural continuity."*

We would also like to emphasize that the central conclusions of the manuscript do not rely on the assumptions of classical polymer brush theory. Rather, they are based on experimentally determined fimbrial abundance combined with Monte Carlo simulations describing the stochastic spatial organization of individual fimbriae. We believe that the revised terminology more accurately reflects the physical nature of the system while leaving the conclusions of the study unchanged.

**Reviewer 3**

**1. Lines 86-87. Please, add a reference supporting this statement.**

**Response:** We thank the Reviewer for this suggestion. An appropriate reference has now been added to support this statement.

**2. Lines 133 and 135. pH6 antigen (Psa), Myf and F1 capsular antigen are composed of a single subunit. Please replace “PsaA/PsaE” with “PsaA” and “MyfA/MyfB” with “MyfA”.**

**Response:** We thank the Reviewer for this comment. The suggested corrections have been incorporated into the revised manuscript.

**3. Lines 219-229. What is the reason for the peak observed in the viscosity plot? Why does the viscosity decrease after 12 h?**

**Response:** We thank the Reviewer for this important question. The relationship between culture viscosity and optical density observed in our experiments is consistent with the general behavior of bacterial cultures reported previously (Sklodowska et al., Micromachines (Basel). 2018 May 21;9(5):251. ).

During the exponential growth phase, the viscosity of the AAEC191A/pCC90 culture increases much more rapidly than that of the non-fimbriated control because the increasing number of bacteria carrying the Dr fimbrial envelope promotes extensive nonspecific cell-cell interactions. This process is facilitated by the relatively low zeta potential of the Dr envelope, which reduces electrostatic repulsion between neighboring cells. As cell density increases, these interactions progressively transform the suspension into a transient, gel-like network, resulting in a marked increase in viscosity.

The viscosity reaches its maximum at the transition to the stationary phase, when both cell density and fimbria-mediated interactions are greatest. Subsequently, despite the optical density remaining relatively constant, viscosity decreases because nutrient depletion and accumulation of metabolic by-products lead to progressive loss of cell viability, partial cell lysis, and weakening of the cohesive fimbria-mediated network. As a result, the transient cell-cell interaction network gradually disintegrates, leading to a pronounced decrease in viscosity. In contrast, the non-fimbriated control never develops such an extensive interaction network and therefore exhibits a substantially lower viscosity peak followed by a more gradual decline.

**4. Lines 337-345. Why does the Dr+ strain show adhesion to xylene but not to hexadecane?**

**Response:** We thank the Reviewer for this interesting observation. We agree that the different behavior of xylene and hexadecane deserves clarification. Although both solvents are commonly used in the MATH assay, they differ considerably in their physicochemical properties. Xylene is an aromatic hydrocarbon with greater polarizability and a higher electron-donor character than the completely apolar aliphatic hydrocarbon hexadecane. We therefore interpret the preferential adhesion of Dr⁺ bacteria to xylene as evidence that the Dr fimbrial envelope is not simply hydrophobic but rather exhibits amphipathic surface properties, allowing favorable interactions with aromatic organic phases while showing little affinity for purely aliphatic hydrocarbons. This interpretation is consistent with our contact angle measurements, which showed only modest changes in overall hydrophobicity, indicating that the Dr envelope modifies the physicochemical character of the bacterial surface rather than rendering it uniformly hydrophobic. We have clarified this point in the revised Discussion.

Furthermore, differences in water–hydrocarbon interfacial tension may affect the transport of bacterial cells across the phase boundary and thus contribute to the apparent differences observed between individual hydrocarbons in the MATH assay.
