# Supplementary material for: Biophysical and functional characterization of the Escherichia coli Dr fimbrial envelope reveals its importance for pathogenesis and lithogenesis
Source: PLoS Pathog. 2026 Sep 16;22(9):e1014588. doi: 10.1371/journal.ppat.1014588 (PMC13608002; doi:10.1371/journal.ppat.1014588)
Supplement: Peer review history

# Author comment: Author response to Decision Letter 0

**DOI:** 10.1371/journal.ppat.1014588.r001

8 Apr 2026

Attachment Submitted filename: Response to Reviewers.docx

# Peer review: Decision Letter 0

**DOI:** 10.1371/journal.ppat.1014588.r002

**Authors:** Matthew C. Wolfgang, Eric Oswald

10 Jun 2026

The biophysical and functional characterization of the Dr fimbrial envelope in E. coli reveals its importance for pathogenesis and lithogenesis

PLOS Pathogens

Dear Dr. Piatek,

Thank you for submitting your manuscript to PLOS Pathogens. After careful consideration, we feel that it has merit but does not fully meet PLOS Pathogens's publication criteria as it currently stands. Therefore, we invite you to submit a revised version of the manuscript that addresses the points raised during the review process.

Please submit your revised manuscript by Aug 09 2026 11:59PM. If you will need more time than this to complete your revisions, please reply to this message or contact the journal office at plospathogens@plos.org. When you're ready to submit your revision, log on to https://www.editorialmanager.com/ppathogens/ and select the 'Submissions Needing Revision' folder to locate your manuscript file.

Please include the following items when submitting your revised manuscript:

* A letter that responds to each point raised by the editor and reviewer(s). You should upload this letter as a separate file labeled 'Response to Reviewers'. This file does not need to include responses to any formatting updates and technical items listed in the 'Journal Requirements' section below.

* A marked-up copy of your manuscript that highlights changes made to the original version. You should upload this as a separate file labeled 'Revised Manuscript with Track Changes'.

* An unmarked version of your revised paper without tracked changes. You should upload this as a separate file labeled 'Manuscript'.

We look forward to receiving your revised manuscript.

Kind regards,

Eric Oswald, Ph.D., D.V.M.

Academic Editor

PLOS Pathogens

Matthew Wolfgang

Section Editor

Editor-in-Chief

PLOS Pathogens

orcid.org/0000-0003-2946-9497

Michael Malim

Editor-in-Chief

PLOS Pathogens

orcid.org/0000-0002-7699-2064

**Additional Editor Comments (if provided):**

**Journal Requirements:**

If the reviewer comments include a recommendation to cite specific previously published works, please review and evaluate these publications to determine whether they are relevant and should be cited. There is no requirement to cite these works unless the editor has indicated otherwise.

1) 1) Please ensure that the CRediT author contributions listed for every co-author are completed accurately and in full.

At this stage, the following Authors/Authors require contributions: Rafał Piatek, Michalina Nagórka, Beata Zalewska-Piątek, Adam Iwanicki, Paweł Wityk, Slawomir Jakiela, Weronika Switlik, Piotr Bruździak, Miłosz Wieczór, Vanessa Liévin-Le Moal, Joanna Raczak-Gutknecht, Przemysław Gnatowski, Mateusz Ficek, Andżelika Łepek, and Magdalena Narajczyk. Please ensure that the full contributions of each author are acknowledged in the "Add/Edit/Remove Authors" section of our submission form.

The list of CRediT author contributions may be found here: https://journals.plos.org/plospathogens/s/authorship#loc-author-contributions

2) PLOS now requires that authors provide the original uncropped and unadjusted images underlying all blot or gel results reported in a submission’s figures or Supporting Information files. This policy and the journal’s other requirements for blot/gel reporting and figure preparation are described in detail at https://journals.plos.org/plosone/s/figures#loc-blot-and-gel-reporting-requirements and https://journals.plos.org/plosone/s/figures#loc-preparing-figures-from-image-files.

When you submit your revised manuscript, please ensure that your figures adhere fully to these guidelines and provide the original underlying images for all blot or gel data reported in your submission. See the following link for instructions on providing the original image data: https://journals.plos.org/plosone/s/figures#loc-original-images-for-blots-and-gels.

In your cover letter, please note whether your blot/gel image data are in Supporting Information or posted at a public data repository, provide the repository URL if relevant, and provide specific details as to which raw blot/gel images, if any, are not available. Email us at customercare@plos.org if you have any questions.

**Reviewers' Comments:**

Reviewer's Responses to Questions

**Part I - Summary**

Please use this section to discuss strengths/weaknesses of study, novelty/significance, general execution and scholarship.

Reviewer #1: This is an interesting study based largely on biophysics based experimentation and microbiology to investigate characteristics of how the production of the Dr fimbrial adhesin can promote specific activities that would potentially contribute to aspects of chronic infection in the urinary tract and also potentially to formation or association with kidney stones by promoting lithogenesis. Further, the expression of these subunits that form a surrounding structure envelope can also potentially provide some resistance to killing by larger bacteriophages (such as P1 and lambda), but not smaller ones such as T7. This suggests in some environments where phage may be present that this could contribute to bacterial persistence. For nearly all of the study, the experimental design used was to compare a Dr+ E. coli strain (producing considerably more Dr adhesin than is present on most strains naturally producing these fimbriae) to a Dr- E. coli strain using the E. coli K-12 type 1 fimbriae-negative strain AAEC191A.

Reviewer #2: (No Response)

Reviewer #3: Nagorka et al. have addressed my concerns and substantially improved the manuscript by focusing on its most important and interesting findings. The revised version is considerably more focused and logically structured. Furthermore, the authors have provided valuable background information that effectively connects their results to previous studies of the FGL (γ3) family of CUP systems and allows several interesting generalizations to be made. Interestingly, the proposed role of Dr envelope in binding to abiotic surfaces and its “shielding” function is also in line with the recently discovered bifunctional role of archaic CUP systems (e.g. Csu pili of A. baumannii) in adhesion and 3D biofilm formation/protective coat formation. I would recommend the paper for publication, provided that the authors address the minor suggestions and corrections listed below.

1. Lines 86-87. Please, add a reference supporting this statement.

2. Lines 133 and 135. pH6 antigen (Psa), Myf and F1 capsular antigen are composed of a single subunit. Please replace “PsaA/PsaE” with “PsaA” and “MyfA/MyfB” with “MyfA”.

3. Lines 219-229. What is the reason for the peak observed in the viscosity plot? Why does the viscosity decrease after 12 h?

4. Lines 337-345. Why does the Dr+ strain show adhesion to xylene but not to hexadecane?

**********

**Part II – Major Issues: Key Experiments Required for Acceptance**

Please use this section to detail the key new experiments or modifications of existing experiments that should be absolutely required to validate study conclusions.

Generally, there should be no more than 3 such required experiments or major modifications for a "Major Revision" recommendation. If more than 3 experiments are necessary to validate the study conclusions, then you are encouraged to recommend "Reject".

Reviewer #1: The experimental model used is not completely representative of a UPEC clinical isolate.

The strain AAEC19A is a fim- derivative of E. coli K-12 strain MG1655 originally described by Ian Blomfield . What the authors need to state is that they are presenting a model for production of Dr+ and Dr- strains by using a K-12 derivative that lacks type 1 fimbriae. They present data that suggests that the Dr + “enveloppe” made using the plasmid system is seemingly similar to that made by the UPEC IH11128 strain based on biophysical comparison. This does not indicate that the K-12 strain is a model strain for the UPEC strain. There are a number of important differences in these strains that could affect many of the experiments presented in this paper. The UPEC strain can also make Type 1 fimbriae and also has a full O antigen (O75) that could affect some of the properties of the interactions concerning viscosity, aggregation, adherence to glass or interactions with kidney stone associated mineral salts.

There are only a few experiments presented to compare levels of Dr adhesin made with the K-12 model compared to the UPEC strain. It is clear from the data that considerably more Dr adhesin is produced in the K-12 Dr+ strain than WT strain IH11128 based on the Western blot results in Fig. S1.

Why was IH11128 only used to demonstrate a comparative size of the Dr enveloppe from a WT clinical strain compared to the one that was used as a model being expressed from a medium (10 to 15) copy plasmid?

All of the data presented other than this demonstration of similar size for the enveloppe was done in a K-12 strain that was engineered not to make type 1 fimbriae. Nearly all E. coli typically make type 1 fimbriae. These fimbriae are also known to be important for UTIs particularly in the bladder. Although they may not be as produced during chronic infection in the kidney where Dr fimbriae may be more relevant, it would be more relevant to place the plasmid-based model into the clinical strain UPEC IH11128 and determine if it functions similarly in this strain background.

Effects of other surface products made by the UPEC strain would be of interest to consider for some of the phenotypes observed. Effects of possibly making type 1 fimbriae or effect of having a full LPS O antigen instead of the K-12 rough LPS importantly could potentially influence the phenotypes of envelope structure or thickness, potential association with kidney stone associated salts, or resistance to phage. It is unclear why the authors did not introduce this plasmid into the UPEC strain to test. It would genetically be cleaner to create a dra knockout mutant in IH11128 and then use the UPEC IH11128 pACYC177 as a Dr-negative and pCC90 as the Dr+ strain. It would need to be a different cloning vector possibly, if the clinical isolate is resistant to ampicillin.

Such an experimental design would more accurately represent features of a true UPEC strain, but with comparison of the added contribution that expression of the Dr fimbriae would have to the various phenotypes examined by the authors.

Reviewer #2: Major Comment 1

The manuscript places strong emphasis on phenotypes that emerge (only?) at very high fimbrial densities (1600 per cell). The authors argue that this regime reflects a naturally occurring high-expressing subpopulation. However, the regulatory basis for achieving and maintaining such extreme expression levels remains unclear. In a phase-variable system, while rare high-expressing cells indeed may arise, the absence of a mechanism that actively sustains elevated expression would be expected to relatively fast result in regression toward a population mean over successive divisions. Consequently, extremely high surface densities are likely to represent transient states rather than maintained phenotypic subpopulations. While this does not say that such rare strongly fimbriated bacteria are not important, their potential roles in pathogenesis thus should be interpreted with this in mind. Not at least since the central conclusions of the manuscript, which is also reflected in the title, rely heavily on phenomena observed at these high densities, the authors should more firmly discuss the physiological relevance of this regime and include thoughts about either how high expression levels can be sustained through regulatory pathways or why this is not important for the suggested function in pathogenesis.

Major Comment 2

Om the same these, the manuscript would benefit from a more detailed and quantitative comparison between the experimental system and native expression levels. The antibody-based fluorescence analysis used to estimate fimbrial abundance is not described in sufficient detail to assess its robustness. Providing information on staining conditions (e.g., antibody concentrations, incubation times, exposure settings), as well as quantitative single-cell intensity distributions, would greatly strengthen this comparison. Demonstrating that the upper range of the native population overlaps with the overexpression regime would be especially important.

An additional suggestion is to make independent physical measurements that could provide valuable complementary validation. For example, if the high-density fimbrial envelope significantly increases the effective size of the bacterium, as suggested, this should be reflected in measurable changes in hydrodynamic radius or diffusivity. Comparing such measurements between native high-expressing cells and the overexpression system could help establish whether they indeed occupy a similar physical regime. Notably, such measurements would also provide an independent means to test key predictions of the computational model presented in the manuscript (see comment 3).

Major Comment 3

The computational framework presented to model the Dr fimbrial envelope is conceptually interesting and provides a useful interpretation of how high-density fimbrial organization may shape the bacterial interface. The observed size-dependent exclusion of larger phages, while allowing smaller phages and molecules to penetrate, is consistent with the existence of a porous fimbrial layer and lends qualitative support to the model. However, several aspects of the model would benefit from further clarification and quantitative grounding.

First, the introduction of the "effective solubility" parameter (σ) appears largely phenomenological. While it is motivated in terms of electrostatic repulsion and hydrophilic swelling, no clear physical mapping or independent constraints are provided for its value. As currently implemented, σ effectively acts as a tunable parameter controlling envelope thickness and density profiles. It would strengthen the manuscript if the authors could relate σ to measurable physical properties (e.g., charge density, ionic strength, filament stiffness) or otherwise motivate the selected range of σ theoretically or experimentally.

Second, the assumption of uniform fimbrial length (≈140–150 subunits per polymer) is likely an oversimplification. In reality, fimbrial assembly is expected to produce a distribution of lengths, which could significantly affect the effective envelope thickness and its radial density profile. In particular, length dispersity would be expected to smooth and potentially reduce the effective outer boundary of the envelope. This limitation should be discussed, and if feasible, incorporated into the modeling framework.

Finally, the model makes useful and testable predictions about the physical extent of the fimbrial layer (200–300 nm). Additional quantitative validation would further strengthen the conclusions. For example, measurements of effective hydrodynamic size or cell diffusivity under different expression levels could provide an independent estimate of envelope thickness and complement the permeability data.

Reviewer #3: Additional experiments are not required

**********

**Part III – Minor Issues: Editorial and Data Presentation Modifications**

Please use this section for editorial suggestions as well as relatively minor modifications of existing data that would enhance clarity.

Reviewer #1: Here are a number of other comments/critiques based on reading of the manuscript :

I recommend changing the title to :

Biophysical and functional characterization of the Escherichia coli Dr fimbrial envelope reveals its importance for pathogenesis and lithogenesis

, as it is inaccurate or abiguous to indicate the envelope is “in” E. coli .

Would Dr fimbrial expression actually be favoured under these conditions of acidic pH ?

Would production of some other type of CUP fimbriae , such as type 1 fimbriae not lead to the same type of interactions with mineralization compared to a negative control strain that is “completely bald” ?

Within the kidney, during UTI, under what biological or environmental conditions would there likely be the presence of bacteriophage that could target E. coli, other than if they were to be used as phage therapy?

Line 67 : replace “their recurrent form “ with “recurrent infection “.

Line 68 : It is important to consider that only certain E. coli strains can make these Dr adhesins. It may be about 10-15% of UPEC strains in general with higher % associated with recurrent or chronic UTIs . Just stating it this way in a summary gives the impression these structural adhesins are always there… The text should indicate “certain E. coli strains or some UPEC “ and not “These bacteria” which the reader would falsely consider as something typically made by all strains. This is clearly not the case.

Line 80: Within the kidney, during UTI, under what biological or environmental conditions would there likely be the presence of bacteriophage that could target E. coli, other than if they were to be used as phage therapy?

Lines 111-112- At this point, you should establish the use of “fimbriae” instead of “pili” throughout your text. Even if both terms are interchangeably used in different articles, it is important to only use one term throughout your text when referring to CUP fimbriae. Ideally at the first use of the term fimbriae in your introduction, at lines 111-112 you can write something like “Fimbriae (also called pili) are filamentous surface structures assembled via the chaperone-usher pathway in many Gram-negative bacteria (15, 16)

Line 120: The term “classical pili” has no strict meaning. The text should be referring to other CUP fimbriae known to be multisubunit filamentous structures such as type 1 or P fimbriae. Although the term pili and fimbriae can be used interchangeably, authors should make the comparison mainly with other types of reference CUP fimbriae known to be extended structures on bacterial surfaces. The term pili again can also refer to other things such as conjugative pili or type IV pili which are structurally distinct, so the wording “classical pili” which could also be interpreted to refer to such distinct bacterial surface structures should not be used.

Lines 144-152: Would be best to still use fimbriae and related terms instead of pili for type 1 and P fimbriae throughout this paragraph, as these are also CUP assembly structures and as such in the text maintaining the term fimbriae for all of these CUP adhesins is recommended for uniformity.

If you are using the term “pili” for type 1 and P pili, then you should also call the Dr adhesins, “Dr pili” I n your manuscript. Ironically based on your results and other work the Dr ans Afa “afimbrial “ adhesins are structurally not polymeric filamentous fimbriae “nor pili “. However, if you routinely use “fimbrial enveloppe” or “fimbriae” to describe Dr adhesins instead of calling them “Dr pili” or a “pili enveloppe”, it would be best for consistency to also use the term fimbriae for type 1 and P fimbrial adhesins instead of “pili” .

Lines 156-174. The text includes use of pili as well as use of fimbriae to describe the same structures. For consistency, since you use fimbriae to describe Dr fimbriae, also use fimbriae to describe other classes of CUP structures such as P or type 1 fimbriae.

Line 209. Where is it or why isn’t it shown what % of cells of Dr+ model produce Dr enveloppe?

Line 597. Authors should specific that E. coli JM101 produces F pili and is normally susceptible to M13 phage.

Line 613. It is incorrect to write that AAEC191A “ is a model strain of UPEC IH11128”. By placing such words in commas after AAEC191A it is suggesting that this strain is a mutant or derivative of the UPEC IH11128. The strain AAEC19A is a fim- derivative of E. coli K-12 strain MG1655 originally described by Ian Blomfield . What the authors need to state is that they are presenting a model for production of Dr+ and Dr- strains by using a K-12 derivative that lacks type 1 fimbriae. They present data that suggests that the Dr + “enveloppe” made using the plasmid system is seemingly similar to that made by the UPEC IH11128 strain based on biophysical comparison. This does not indicate that the K-12 strain is a model strain for the UPEC strain. There are a number of important differences in these strains that could affect many of the experiments presented in this paper. The UPEC strain can also make Type 1 fimbriae and also has a full O antigen (O75) that could affect some of the properties of the interactions concerning viscosity, aggregation, adherence to glass or interactions with kidney stone associated mineral salts. As such, authors have a model based on a fimbriae-negative strain that can compare effects of production of Dr adhesin on various phenotypes that they have tested.

Using this simplified model allows the authors to investigate biophysical properties of a Dr enveloppe produced by a fim-negative E. coli K-12 strain.

Line 632. Correct spelling of fimbriae

Line 781. Although there may not have been reports of phage inhibition through formation of a physical barrier by fimbriae, type 1 fimbriae have been shown to prevent or resist killing by a type VI secretion system recently…

mBio. 2024 Mar 18;15(4):e02553-23. doi: 10.1128/mbio.02553-23. Dessartine et al. 2024

Type 6 secretion killing is similar in mechanism to bacteriophage-mediated killing.

Line 849…. Why isn’t the strain IH11128 described in the bacterial strains section as a urinary tract pathogenic (UPEC) strain?

Reviewer #2: Minor Comment 1

The manuscript presents a substantial and interesting body of experimental work however the organization and writing style could be improved to enhance clarity and readability. Several sections describing experiments that address the functional impact of the Dr fimbrial envelope appear repetitive. Bringing related experiments together and supporting them with more integrated figures or graphical summaries would improve the overall flow and make the narrative easier to follow. This could also significantly shorten the manuscript, which is now longer than needed without adding proportional clarity. In addition, some Results subsections end with brief interpretative or concluding statements, while others do not. Given the clear separation between Results and Discussion, it would be more consistent to reserve interpretation and synthesis for the Discussion section.

Minor Comment 2

The authors describe the fimbrial layer using terminology such as “supramolecular assembly” and “brush-like” organization, which suggests an analogy to grafted polymer brushes. While this conceptual framing is intuitively appealing, it may not be physically well justified in the present system and would benefit from further clarification.

Classical polymer brush behavior arises in the regime of very high grafting density, where excluded volume interactions between neighboring chains generate strong entropic stretching, leading to a relatively well-defined, surface-like polymer boundary. In contrast, the estimated spacing between fimbriae in this work (on the order of tens of nanometers) suggests a comparatively low grafting density, where intermolecular interactions are expected to be weak and infrequent. In such a regime, one would expect a more diffuse, heterogeneous three-dimensional distribution of filaments rather than a sharply defined “brush” interface.

This distinction has important implications for how the fimbrial layer is interpreted. Rather than behaving as a classical brush with a well-defined height, the Dr fimbrial coating may be better described as a sparse, flexible fibrous network forming a volumetric layer with a gradually decaying density profile. Referring to the outer boundary of this layer as a “surface” may therefore be conceptually misleading, as no clear physical interface is expected to emerge in the absence of strong collective stretching or packing effects.

Moreover, if steric interactions between fimbriae are indeed limited, the organization of the layer is likely governed primarily by individual filament flexibility and stochastic spatial distribution, rather than cooperative entropic effects characteristic of dense polymer brushes. Clarifying this distinction would help avoid overinterpretation of the polymer brush analogy and better align the model with the expected physical regime of the system.

Reviewer #3: (No Response)

**********

PLOS authors have the option to publish the peer review history of their article (what does this mean?). If published, this will include your full peer review and any attached files.

If you choose “no”, your identity will remain anonymous but your review may still be made public.

**Do you want your identity to be public for this peer review?**  For information about this choice, including consent withdrawal, please see our Privacy Policy.

Reviewer #1: **Yes:**  Charles M. Dozois

Reviewer #2: No

Reviewer #3: **Yes:**  Anton Zavialov

[NOTE: If reviewer comments were submitted as an attachment file, they will be attached to this email and accessible via the submission site. Please log into your account, locate the manuscript record, and check for the action link "View Attachments". If this link does not appear, there are no attachment files.]

**Figure resubmission:**

**Reproducibility:**

?>

# Author comment: Author response to Decision Letter 1

**DOI:** 10.1371/journal.ppat.1014588.r003

5 Aug 2026

Attachment Submitted filename: Response_to_reviewers_auresp_1.docx

# Peer review: Decision Letter 1

**DOI:** 10.1371/journal.ppat.1014588.r004

**Authors:** Matthew C. Wolfgang, Eric Oswald

1 Sep 2026

Dear Dr. Piatek,

We are pleased to inform that your manuscript, "Biophysical and functional characterization of the Escherichia coli Dr fimbrial envelope reveals its importance for pathogenesis and lithogenesis", has been editorially accepted for publication at PLOS Pathogens.

Before your manuscript can be formally accepted and sent to production, you will need to complete our formatting changes, which you will receive by email within a week. Please note that your manuscript will not be scheduled for publication until you have made the required changes.

IMPORTANT NOTES

(1) Copyediting and Proofreading: The corresponding author will receive a typeset proof for review, to ensure errors have not been introduced during production. Please review the PDF proof of your manuscript carefully, as this is the last chance to correct any errors. Please note that major changes, or those which affect the scientific understanding of the work, will likely cause delays to the publication date of your manuscript.

(2) Appropriate Figure Files: Please remove all name and figure # text from your figure files. Please also take this time to check that your figures are of high resolution, which will improve the readbility of your figures and help expedite your manuscript's publication. Please note that figures must have been originally created at 300dpi or higher. Do not manually increase the resolution of your files. For instructions on how to properly obtain high quality images, please review our Figure Guidelines, with examples at: http://journals.plos.org/plospathogens/s/figures.

We encourage you to use PACE (http://pace.apexcovantage.com/), a figure-checking tool which helps authors ensure all figures meet PLOS requirements so that figures can publish in the highest quality. PACE is a digital diagnostic and conversion tool for figure files. It will provide information about any failed check(s) and, if able, will automatically convert the figure into an acceptable file that passes quality checks.

To use PACE, you must first register as a user. Then, login and navigate to the UPLOAD tab, where you will find detailed instructions on how to use the tool.

(3) Striking Image: Please upload a striking still image to accompany your article if one is available (you can include a new image or an existing one from within your manuscript). Should your paper be accepted, this image will be considered for our monthly issue image and may also appear on our website to feature your article. Please upload this as a separate file, selecting "striking image" as the file type upon upload. Please also include a separate "Other" file with a caption, including credits and any potential copyright information. Please do not include the caption in the main article file. If your image is from someone other than yourself, please ensure that the artist has read and agreed to the terms and conditions of the Creative Commons Attribution License at http://journals.plos.org/plospathogens/s/content-license. Please note that PLOS cannot publish copyrighted images.

(4) Press Release or Related Media: If you or your institution will be preparing press materials for this manuscript, please inform our press team in advance at plospathogens@plos.org as soon as possible. We ask that you contact us within one week to plan ahead of our fast Production schedule. If you need to know your paper's publication date for related media purposes, you must coordinate with our press team, and your manuscript will remain under a strict press embargo until the publication date and time.

(5) Update your Profile Information: Now that your manuscript has been provisionally accepted, please log into Editorial Manager and update your profile, if needed. Go to http://www.editorialmanager.com/ppathogens, log in, and click on the "Update My Information" link at the top of the page. Please update your user information to ensure an efficient production and billing process.

(6) LaTeX users only: Our staff will ask you to upload a TEX file in addition to the PDF before the paper can be sent to typesetting, so please carefully review our Latex Guidelines http://journals.plos.org/plospathogens/s/latex in the meantime.

Thank you again for supporting Open Access publishing; we are looking forward to publishing your work in PLOS Pathogens.

Best regards,

Eric Oswald, Ph.D., D.V.M.

Academic Editor

PLOS Pathogens

Matthew Wolfgang

Section Editor

PLOS Pathogens

Dominique Soldati-Favre

Editor-in-Chief

PLOS Pathogens

***********************************************************

Reviewer's Responses to Questions

**Part I - Summary**

Please use this section to discuss strengths/weaknesses of study, novelty/significance, general execution and scholarship.<br/><br/>

Reviewer #1: The paper explores aspects of the phenotypic changes and biophysical properties that production of Dr fimbrial envelope can have on E. coli. The work is pertinent. Although some aspects of the actual properties of the Dr fimbriae in the context of the uropathogenic strains that typically express them transiently or by a sub-group of a population at any time, as that are controlled by phase variation, the use of a non-fimbriated (type 1 fimbriae-negative E. coli K-12 strain) model provides more direct physical data of the individual properties of Dr expression at the surface of E. coli cells.

Reviewer #2: The authors have addressed my previous concerns satisfactorily. The newly added experiments using the clinical UPEC isolate are particularly important, as they convincingly demonstrate that the phenotypes identified in the constitutively expressing model strain are also present in the naturally occurring Dr-positive subpopulation of the wild-type strain. This significantly strengthens the physiological relevance of the study and establishes that the model system is representative of the biological phenomenon under investigation.

Although I still consider some aspects of the computational framework to be more illustrative than mechanistically predictive, its role in the revised manuscript is appropriately balanced and its limitations are acknowledged. I also appreciate the authors' efforts to adopt more physically accurate terminology when discussing the organization of the fimbrial layer.

I was also interested to learn about the authors' ongoing efforts to develop alternative physicochemical approaches for characterizing fimbrial envelopes. In particular, extending such methods to heterogeneous populations may provide valuable future insight into the biological significance of variability in fimbrial expression states and further test predictions arising from the current work.

Taken together, the revisions have substantially strengthened the manuscript, and I support publication without further changes.

Reviewer #3: The authorsIn their study, Nagórka et al. performed a biophysical and functional characterization of the Dr fimbrial "envelope" in E. coli and demonstrated that Dr fimbriae may (1) enhance bacterial protection against phages and (2) mediate binding to non-organic materials such as hydroxyapatite and calcium oxalate crystals. In my view, these two findings are both novel and significant. The authors’ multidisciplinary approach to investigating the biophysical properties of Dr fimbriae is interesting.

--------------------

**Part II – Major Issues: Key Experiments Required for Acceptance**

Please use this section to detail the key new experiments or modifications of existing experiments that should be absolutely required to validate study conclusions.<br/><br/>Generally, there should be no more than 3 such required experiments or major modifications for a "Major Revision" recommendation. If more than 3 experiments are necessary to validate the study conclusions, then you are encouraged to recommend "Reject".<br/><br/>

Reviewer #1: I have verified the revisions and reformulation of the text to "tone down" some of the conclusions of the findings from the study to assert that the K-12 model may not be completely representative of the role of Dr adhesin in certain UPEC strains.

I am overall satisfied with the revisions.

Reviewer #2: (No Response)

Reviewer #3: The authors have addressed my comments, and I do not see any major issues with the paper.

--------------------

**Part III – Minor Issues: Editorial and Data Presentation Modifications**

Please use this section for editorial suggestions as well as relatively minor modifications of existing data that would enhance clarity.<br/><br/>

Reviewer #1: (No Response)

Reviewer #2: (No Response)

Reviewer #3: I just want to comment on the terminology issue raised by another reviewer. I agree that using either the term fimbriae or pili consistently throughout the paper would help improve clarity, but these terms are essentially interchangeable. In my opinion, it is more important to mention the chaperone–usher pathway (CUP) and use the terms CUP fimbriae or CUP pili.

The reviewer also raised some skepticism about the term “classical pili.” In my opinion, the phylogenetic classification of CUP systems into classical, alternative, and archaic CUP families remains very useful, especially in light of recent structural studies. Pakharukova et al. showed that classical and non-classical systems (i.e., alternative and archaic systems) have significant differences in their assembly mechanisms (Pakharukova et al. 2015, PLOS Pathogens; Pakharukova et al. 2018, JBC). Furthermore, archaic systems have a distinct subunit fold (Pakharukova et al. 2018, JBC), pilus architecture (Pakharukova et al. 2022, Nature), and a bifunctional mode of action involving hydrophobic tip-finger adhesion (Pakharukova et al. 2018, PNAS) and bacterial cross-linking (Malmi et al. 2026, Nature Communications).

I suggest sticking to this helpful phylogenetic classification, although, unfortunately, the names themselves may seem somewhat misleading.

--------------------

PLOS authors have the option to publish the peer review history of their article (what does this mean?). If published, this will include your full peer review and any attached files.

If you choose “no”, your identity will remain anonymous but your review may still be made public.

**Do you want your identity to be public for this peer review?**  For information about this choice, including consent withdrawal, please see our Privacy Policy.

Reviewer #1: Yes: Charles M. Dozois

Reviewer #2: No

Reviewer #3: Yes: Anton Zavialov

# Editor report: Acceptance letter

**DOI:** 10.1371/journal.ppat.1014588.r005

**Authors:** Matthew C. Wolfgang, Eric Oswald

Dear Dr. Piatek,

We are delighted to inform you that your manuscript, "Biophysical and functional characterization of the Escherichia coli Dr fimbrial envelope reveals its importance for pathogenesis and lithogenesis," has been formally accepted for publication in PLOS Pathogens.

We have now passed your article onto the PLOS Production Department who will complete the rest of the pre-publication process. All authors will receive a confirmation email upon publication.

Thank you again for supporting open-access publishing; we are looking forward to publishing your work in PLOS Pathogens.

Best regards,

Dominique Soldati-Favre

Editor-in-Chief

PLOS Pathogens
